# Towards the co‐ordination of terrestrial ecosystem protocols across European research infrastructures

**DOI:** 10.1002/ece3.2997

**Published:** 2017-04-23

**Authors:** Les G. Firbank, Chiara Bertora, David Blankman, Gemini Delle Vedove, Mark Frenzel, Carlo Grignani, Elli Groner, Miklós Kertész, Eveline J. Krab, Giorgio Matteucci, Christina Menta, Carsten W. Mueller, Jutta Stadler, William E. Kunin

**Affiliations:** ^1^ School of BiologyUniversity of LeedsLeedsUK; ^2^ Department of Agriculture, Forest and Food ScienceUniversità degli Studi di TorinoTorinoItaly; ^3^ Information Management, Israel LTERBen‐Gurion University of the NegevBeer‐ShevaIsrael; ^4^ Department of Agricultural, Food, Environmental and Animal SciencesUniversity of UdineUdineItaly; ^5^ Department of Community EcologyHelmholtz Centre for Environmental Research ‐ UFZHalle/SaaleGermany; ^6^ Dead Sea and Arava Science CenterMitzpe RamonIsrael; ^7^ MTA Centre for Ecological ResearchInstitute of Ecology and BotanyVácrátótHungary; ^8^ Department of Ecological ScienceVU University AmsterdamAmsterdamThe Netherlands; ^9^ CNR ISAFOMNational Research Council of ItalyInstitute for Agriculture and Forestry Systems in the MediterraneanErcolanoNAItaly; ^10^ Department of ChemistryLife Sciences and Environmental SustainabilityUniversity of ParmaParmaItaly; ^11^ Lehrstuhl für BodenkundeResearch Department Ecology and Ecosystem ManagementTechnische Universitaet MuenchenMunichGermany; ^12^ Present address: Climate Impacts Research CentreAbisko Naturvetenskapliga StationEcology and Environmental SciencesUmeå UniversityAbiskoSweden

**Keywords:** biogeochemical cycles, ecological Integrity, ecological processes, long term ecological research, quality assurance of ecological data

## Abstract

The study of ecosystem processes over multiple scales of space and time is often best achieved using comparable data from multiple sites. Yet, long‐term ecological observatories have often developed their own data collection protocols. Here, we address this problem by proposing a set of ecological protocols suitable for widespread adoption by the ecological community. Scientists from the European ecological research community prioritized terrestrial ecosystem parameters that could benefit from a more consistent approach to data collection within the resources available at most long‐term ecological observatories. Parameters for which standard methods are in widespread use, or for which methods are evolving rapidly, were not selected. Protocols were developed by domain experts, building on existing methods where possible, and refined through a process of field testing and training. They address above‐ground plant biomass; decomposition; land use and management; leaf area index; soil mesofaunal diversity; soil C and N stocks, and greenhouse gas emissions from soils. These complement existing methods to provide a complete assessment of ecological integrity. These protocols offer integrated approaches to ecological data collection that are low cost and are starting to be used across the European Long Term Ecological Research community.

## 1 Introduction

It is now accepted that mankind is manipulating at least some biogeochemical processes at global scales, forcing the Earth outside at least some of its safe operating limits (Rockstrom et al., 2009). In order to seek to keep the Earth system within its planetary boundaries, it is vital to understand the ecological processes involved. Key research questions therefore focus on the response of ecosystems respond to human and natural forcings, including changing climate, land use and species invasions, and quantifying the processes that underpin these responses over multiple scales of space and time (Anderson, Bales, & Duffy, 2008; Heffernan et al., 2014; Keller, Schimel, Hargrove, & Hoffman, 2008). While some of the required information is directly available at multiple scales (e.g., through remote sensing), large scale ecological data are more typically obtained by integrating distributed samples taken at much smaller scales (Borer et al., 2014; Fraser et al., 2013). As with any sampling exercise, the value of such data is increased when they are obtained to common standards (Keller et al., 2008; Osenberg, Sarnelle, Cooper, & Holt, 1999). For example, the well‐resourced US National Ecosystem Observatory Network (NEON) uses very tightly specified data standards (Keller et al., 2008), while the FLUXNET network of GHG flux sites allows more variation in methods according to the resources available locally (Baldocchi et al., 2001).

However, this approach does not exploit the opportunities offered by existing long‐term ecosystem observatories. There are 43 national networks of Long‐Term Ecological Research (LTER) sites around the world, established by local institutions with interests in quantifying temporal ecosystem change in specific ecosystems. Many of these sites have developed impressive time series of data, capable of being used in ways not imagined when data collection started: For example, soils sampled from the Rothamsted classic experiments were used much later to test for signs of atmospheric nuclear tests (Woiwod, 1991). The problem for multiple use of data in comparative studies is that protocols have been developed on a site‐by‐site basis. It is hard to persuade ecologists to adopt new protocols when there has already been considerable investment in collecting long‐term data, and when financial support for making changes is missing.

This study responds to this challenge by building a scientific consensus around a priority set of standardized protocols for measuring parameters that are important indicators of ecosystem state and process, simple enough to be used by nonspecialists with basic training, yet are not already covered by international standard approaches. They are, in principle, appropriate for controlled environments, field experiments and observatories, and can be used in larger‐scale studies if supported by appropriate metadata management.

## 2 Protocol Selection

The approach was to engage with the European research community to prioritize particular terrestrial ecosystem parameters relevant to larger scale research that may benefit from the use of common protocols; to develop these protocols through information review and expert knowledge; and to test and fine tune them using training courses.

Potential parameters were classified into the framework of “ecological integrity” (Muller, Hoffmann‐Kroll, & Wiggering, 2000) which is applied within the LTER community. Ecological integrity is a concept intended to guide decisions so that ecosystem services are safeguarded and the capability for ecological self‐organization is not disrupted. This framework comprises both ecosystem structures, emphasizing biotic diversity, abiotic heterogeneity, and ecosystem processes across scales, using budgets of energy, matter, and water (Table 1). It can be applied across ecosystems and has been used to quantify ecosystem condition at the pan‐European scale (Stoll et al., 2015).

**Table 1:** The components and basic indicators of ecological integrity, noting which were used as the basis for new protocols in this project

Level 1 components	Level 2 components	Ecological integrity indicators	Protocols developed in this study	Example protocols and procedures already widely used	Example techniques being developed
Ecosystem structures	Biotic diversity	Flora diversity		Vegetation classifications (Rodwell, 1991‐2000) Diversity metrics (Butchart et al., 2010; Gotelli & Colwell, 2001)	Molecular methods (Kress, Wurdack, Zimmer, Weigt, & Janzen, 2005)
		Fauna diversity	Soil mesofaunal diversity	Species indicators (Billeter et al., 2008), trophic structure (Cardinale et al., 2006)	Molecular methods (Torsvik & Ovreas, 2002; Yu et al., 2012)
		Within habitat structure		Forest canopy structure (McElhinny, Gibbons, Brack, & Bauhus, 2005)	Fractal dimension (Kamal, Lee, & Warnken, 2014), habitat roughness (de Thoisy et al., 2016)
	Abiotic heterogeneity	Soil		Soil classification (International Union of Soil Scientists 2015)	
		Water		Freshwater habitat classification (Frissell, Liss, Warren, & Hurley, 1986)	
		Atmosphere		Meteorological standard variables (World Meteorological Organization 2014)	
		Habitat	Land use and management	Land use diversity, landscape structure (Tscharntke et al., 2012)	Landscape roughness (McGarigal, Tagil, & Cushman, 2009)
Ecosystem Processes	Energy budget	Input	Leaf area index	Leaf area ((ICP Forests 2016)) Radiation (World Meteorological Organization 2014)	Remote sensing estimates (Running et al., 2004)
		Storage	Above‐ground biomass; Soil organic matter—carbon and nitrogen stocks	Net Primary Production (Danescu, Albrecht, & Bauhus, 2016)	Remote sensing estimates (Ardö, 2015)
		Output	GHG emissions from soils	Respiration (Baldocchi, 2008)	Remote sensing estimates (Ardö, 2015)
		Efficiency measures		Energy balance (Stoy et al., 2013)	
	Matter budget	Input		Deposition of, for example, sulfate, chloride, nitrate (ICP Forests 2016)	
		Storage	Above‐ground biomass	Net Primary Production (Danescu et al., 2016)	Remote sensing estimates (Ardö, 2015)
			Leaf area index	Leaf area (ICP Forests manual version 2016)	Remote sensing estimates (Running et al., 2004)
			Soil organic matter—carbon and nitrogen stocks	Soil carbon stocks (Stolbovoy et al., 2005), Soil organic carbon, soil nitrogen (ICP Forests 2016)	Large scale inventory and modelling approaches (Martin et al., 2016; Wiesmeier et al., 2013)
		Output	GHG emissions from soils	Carbon dioxide fluxes (Baldocchi, 2008)	
		Efficiency measures	Decomposition rate	Soil organic matter (Schmidt et al., 2011)	
	Water budget	Input		Precipitation (World Meteorological Organization 2014)	
		Storage		Soil moisture (World Meteorological Organization 2014)	Remote sensing (Nichols, Zhang, & Ahmad, 2011)
		Output	Leaf area index	Potential evapotranspiration (World Meteorological Organization 2014)	Remote sensing (Nouri, Beecham, Anderson, Hassanli, & Kazemi, 2015)
		Efficiency measures		Ratio transpiration/evaporation	

See text for details.

The European LTER community had already collated a suite of metrics, each assigned to ecosystem structures and processes according to this framework (Frenzel et al., 2012). Of this suite, seven parameters were selected by consulting with the research community, using a questionnaire followed by a workshop. They cover a broad range of ecological integrity indicators for which the development of standardized protocols was regarded as having scientific value and broad acceptability. They were proposed for use by scientists across large numbers of sites without top‐down funding or direction. These were simple to apply without specialist training or equipment, unambiguous and addressed ecologically meaningful parameters. Protocols for which standard methods are already very widely used (e.g., meteorological data) or for which methods are still under rapid development (e.g., soil metagenomics (Hirsch, Mauchline, & Clark, 2010)) were not selected. Feedback from training courses was used to further refine the protocols.

Each of the seven parameters corresponds to at least one of the five level 2 components of ecological integrity (Table 1); they are to be complemented by other protocols already widely used in a standardized format to obtain a more complete assessment of ecological integrity. The selected protocols are based on methods already used, but not applied consistently. They do not detail when, where or how many samples are required. This is because they are generic in nature and are not yet integrated into a formal integrated sampling program. It is recommended that metadata are managed using Dynamic Ecological Information Management System (DEIMS; https://data.lter-europe.net/deims/), the research site and dataset registry for long‐term ecological observatories and experimental platforms.

Outlines of the protocols are given here; the handbook with details is available online (http://www.expeeronline.eu/outputs/expeer-protocols.html) and as supporting information (Appendix S1).

## 3 The Protocols

### 3.1 Land use and management

Land use and management data are needed to define the ecological integrity indicators of biotic diversity and abiotic heterogeneity; to inform energy, water and matter budgets, and provide important metadata for ecological studies. This protocol was included to ensure that contextual information about study sites would be routinely collected to common standards, supporting the requirements for metadata. Data are required for each spatial unit of the site with consistent management, which could be a field, an area of grassland managed as a unit (e.g., Rodwell, 1991‐2000), a plot within an experiment (Steinbeiss et al., 2008), or a chamber within an Ecotron (e.g., Bradford et al., 2002; Milcu et al., 2014)). The use of standardized definitions allows upscaling of results to larger areas (Bunce, Barr, Clarke, Howard, & Lane, 1996). The protocol involves recording

- the description of the spatial unit, its location and area;
- for field sites, vegetation cover as defined using Level 3 of the European Nature Information System (EUNIS) Habitats Classification (De Graaf, Bobbink, Smits, Van Diggelen, & Roelofs, 2009). EUNIS is widely used across Europe, and Level 3 requires no specialist knowledge;
- data on ecosystem manipulations, including inputs, outputs, agricultural and forestry management, land use history (if known).

### 3.2 Soil meso‐faunal diversity

Soil faunal diversity relates to the ecological integrity element “Biotic diversity” and is an indicator of soil quality, and hence of the long‐term sustainability of an ecosystem (Schoenholtz, Van Miegroet, & Burger, 2000). Soil fauna mediate C and N dynamics, and changes in soil faunal diversity and food web complexity have been linked to alterations in ecosystem functioning (Bardgett & Cook, 1998) and resilience to environmental disturbances.

The QBS‐ar index (Soil Biological Quality) is a recently developed biodiversity index (Parisi, Menta, Gardi, Jacomini, & Mozzanica, 2005) that is simple and robust enough for soil quality assessment over very large numbers of highly contrasting sites, complementing more traditional approaches based on the use of physical, chemical, and microbiological indicators. QBS‐ar indicates the degree of naturalness and degradation. The concept is that the higher the soil quality, the higher the number of morphologically distinct microarthropod groups (Parisi et al., 2005), each of which has its own score (Table 2); values are combined to give the overall QBS‐ar index. Generally, woodlands have the highest values, followed by uncultivated lands and meadows. Degraded soils are in the middle, followed by cropped lands. The QBS‐ar index has been used successfully to test for the effects of forest cutting, grazing, trampling, industrial activities, emission, agriculture, heavy metals, and other anthropogenic effects (Gardi, Menta, & Leoni, 2008; Menta, Conti, Pinto, Leoni, & Lozano‐Fondoon, 2014).

**Table 2:** EMI values for the computation of the QBS‐ar soil biodiversity index. See text for details

Taxa		EMI
Pseudoscorpiones		20
Scorpions	Juvenile	10
Palpigradi		20
Opiliones		10
Araneae	Forms >5 mm	1
	Small forms, scarcely pigmented	5
Mites		20
Isopoda		10
Diplopoda	Forms >5 mm	10
	Forms <5 mm	20
Pauropoda		20
Symphyla		20
Chilopoda	Forms > 5 mm, well‐developed legs	10
	Other forms (Geofilomorfi)	20
Protura		20
Diplura		20
Collembola	Clearly epigeous forms: middle to large size, complex pigmentation present, long, well‐developed appendages, well‐developed visual apparatus (eye spot and eyes)	1
	Epigeous forms not related with grass, shrubs or trees, well‐developed appendages, (possible) well‐developed setae or protective cover of scales, well‐developed visual apparatus	2
	Small size—although not necessarily—forms, usually limited to litter, with modest pigmentation, average length of appendages, developed visual apparatus	4
	Hemi‐edaphic forms with visual apparatus still developed, not elongated appendages, cuticle with pigmentation	6
	Hemi‐edaphic forms with reduced number of ommatidio, scarcely developed appendages, often short or absent furca, pigmentation present	8
	Eu‐edaphic forms with no pigmentation, reduction or absence of ommatidia, furca present—but reduced	10
	Clearly eu‐edaphic forms: no pigmentation, absent furca, short appendages, presence of typical structures such as pseudo‐oculi, developed postantennal organs (character not necessarily present), apomorphic sensorial structures	20
Microcoryphia		10
Zygentomata		10
Dermaptera		1
Orthoptera	In general	1
	Grillidae family	20
Embioptera		10
Phasmids		1
Mantoidei		1
Mecoptera		1
Isoptera		10
Blattaria		5
Psocoptera		1
Hemiptera	In general, mostly epigeous (above‐ground) or root‐feeding forms	1
	Cicada larvae	10
Raphidioptera		1
Thysanoptera		1
Coleoptera	Clearly epigeous forms	1
	Dimensions <2 mm	+4
	Thin integument, often testaceous (tan‐brown) colour	+5
	Hind wings highly reduced or absent	+5
	Microphtalmy or anophtalmy	+5
	Edaphic forms	20
Hymenoptera	In general	1
	Formicidae	5
Diptera	Adults	1
Rafidiotteri		10
Planipenni		1
Mecoptera (larve)		10
Coleoptera (larve)		10
Diptera (larve)		10
Hymenoptera (larve)		10
Lepidoptera (larve)		10
Other holometabolous	Adults	1

The protocol (Gardi et al., 2008) should be completed annually in stable soils (at the same time during the year, normally in the spring or autumn) and more frequently in arable systems. Soil is taken with a standard soil corer (10 cm diameter and 10 cm deep) at the selected location after removing the litter layer. Microarthropods are extracted from soil cores using a Berlese‐Tullgren funnel in which heat from a lamp causes the arthropods to escape and eventually fall into a solution of 75% alcohol and 25% glycerine by volume. The microarthropods are identified by class for miriapods (Diplopoda, Chilopoda, Symphyla, Pauropoda) and order for insects, Chelicerata and Crustacea. The specimens belonging to each taxon are then counted and separated into biological forms (Table 2). Each form is associated with a score (EMI—Eco‐Morphological Index), which ranges from 1 to 20 in proportion to its degree of adaptation to soil. The QBS‐ar index is obtained by the EMI sum of all collected groups.

### 3.3 Soil organic matter—carbon and nitrogen stocks

Stocks of nitrogen and carbon stocks in soils relate to the ecological integrity elements of energy and matter budgets. The data are needed for biogeochemical and earth system modelling, especially when combined with protocols for land use and management, decomposition, and GHG emissions. Standard methods are already available yet are not widely used among the LTER community. This protocol follows Stolbovoy, Montanarella, Filippi, Selvaradjou, and Gallego (2005).

A composite soil sample is taken from several spots around a central soil pit, either by soil horizons or by fixed depth intervals of 10–30 cm, ideally down to the parent material (C horizons). In mineral soils, steel rings of 100 cm^3^ are usually used to sample a known volume. The soil samples are promptly air dried, sieved over a sieve of 2 mm mesh size and homogenized. Soil aliquots must be dried at 105°C for 24 hr. The most common method to analyze C and N concentrations is laboratory‐based dry combustion using an elemental analyzer. When the pH exceeds 7, a parallel carbonate destruction and inorganic carbon quantification is required, either by combustion of the organic C at 550° for at least 4 hours, or by acid treatment using, for example, HCl. Bulk density is crucial for all determinations of element stocks and is measured by drying a known soil volume at 105°C for at least 24 hr to constant weight. Any larger particles will have been removed by sieving; these are weighed and assumed to have a density of 2.65 g cm^3^. The organic layer within a “counting frame” (e.g., square frame 20 × 20 cm) can be removed, dried, and weighed if a separate determination is required.

### 3.4 Decomposition

Decomposition is relevant to ecological integrity elements “matter budget” and “energy budget” and indicates matter loss and nutrient cycling. It is influenced by both abiotic parameters (including soil chemistry, temperature, and moisture) and biotic factors (e.g., litter substrate quality and the range of decomposer organisms) (Cornelissen, 1996). Litter bags and bait lamina are appropriate for assessing decomposition in both terrestrial and aquatic ecosystems, with litter bags more sensitive to microbial activity and bait lamina to soil fauna; they can therefore be used together (van Gestel et al., 2009).

Litter bags, 10 × 10 cm, are filled with a 2‐g dried standard litter substrate and placed randomly in the litter layer in the field. The substrate can be monospecific or polyspecific, contain local, natural, cultivated, invasive, or nonlocal species. Mesh size of litter bags determines the decomposition process being measured; mesh sizes below 100 μm enable only fungi and bacteria to colonize, while bags with a mesh size of 1 mm or wider also enable access by invertebrates. The hypothesis to be tested therefore determines substrate and mesh size. After several weeks or months, the litterbags are re‐weighed; the weight loss is the measure of decomposition (e.g., Chen et al., 2017).

The bait lamina method indicates the feeding activity of the soil fauna and is little influenced by microbiological activity. The bait lamina strip is a made from PVC and is about 15 cm length, with up to 16 conical holes, each filled with a bait mixture that contains fine ground cellulose (70%) and bran (30%) powder together with a small amount of activated charcoal (Kratz, 1998). Bait lamina strips are placed in the soil with the uppermost hole positioned just beneath the soil surface. The bait lamina strips are removed when more than 40% of the bait is eaten. When comparing the feeding activity at different study sites, the bait laminas need to be removed at exactly the same time span of exposure at all sites. The metric is the proportion of bait eaten on each stick (e.g., Griffiths et al., 2016).

### 3.5 Above‐ground biomass

The increase and/or harvest of above‐ground plant biomass over a year corresponds to the ecological integrity element “matter budget”, and relates to the “energy budget” because of its relation to photosynthetic capacity and autotrophic respiration. Aboveground biomass is an appropriate estimator of annual net primary production, important in many ecosystem models.

Data are generated in terms of dry mass per unit area by species. For forests, the approach (following Scarascia‐Mugnozza, Oswald, Piussi, and Radoglou 2000) is to build an allometric relationship between shoot biomass and a parameter easily measured in living trees, for example, tree diameter and then estimate total above‐ground biomass by applying the allometric relationship across all trees in the required area. Estimating the allometric relationship involves harvesting and weighing a sample of trees that represent the range of sizes (and, if required, species) in the stand. For grasslands and crops, the protocol (based on Milner and Hughes (1968)) involves removing, drying, and weighing all above‐ground plant material from sample areas. Harvesting should take place at the yearly maximum of above‐ground biomass, preceding any agricultural harvesting, and may often need to be multi‐annual.

### 3.6 Leaf area index

Leaf area index (LAI) is defined as the total one‐sided foliage area per unit ground surface area. It relates to the ecological integrity element “energy budget,’ indicating energy capture through photosynthesis, and also “Water Budget,” relating to evapotranspiration. It is a key variable in various models of Net Primary Production.

LAI from deciduous trees can be measured using standard forestry procedures (ICP Forests 2016), by collecting leaves falling into in at least 10 litter traps (funnels), weighing them and assessing the ratio leaf area to weight on a subsample of collected leaves. Litterfall should be collected monthly, and more frequently in periods of heavy fall (e.g., after heavy rain in autumn). Dry leaves may need to be soaked before taking area measurements using an LAI meter or scanner; wet leaves may need to be cleaned and flattened. In evergreen forests, falling needles do not equal standing leaf area and LAI should be made using allometric relationships with a more easily measured parameter, such as tree diameter. Indirect methods of assessing LAI involve assessment of light interception, either using the analysis of hemispherical photographs or instruments detecting the fraction of light intercepted by the canopy. Multiple measurements should be taken during the season, to account for phenology, ensuring that LAI is assessed at its maximum (normally in the centre of the growing season). In grasslands and croplands, LAI can be determined by harvesting small parcels of vegetation. The material should be weighed to determine the specific leaf area (SLA) (the leaf area to unit of weight) for each species present or on subsamples of collected material. SLA varies greatly by growth form, species and phenological stage.

### 3.7 Greenhouse gas emissions (GHGs) from soils

Soil organic carbon (SOC) and soil organic nitrogen are major sources of the three main GHGs, namely CO_2_, N_2_O, and CH_4_. GHG emissions from soils are therefore central to models of ecosystem processes and GHG inventories. They relate to the ecological integrity elements “energy budget” and “matter budget.” Significant efforts have already been made toward standardization of techniques, mainly by USDA program GRACEnet and by the Global Research Alliance on Agricultural Greenhouse Gases (e.g., Collier, Ruark, Oates, Jokela, & Dell, 2014).

This protocol measures the efflux of greenhouse gases CO_2_, N_2_O, and CH_4_ from soils using chambers that rest on the soil surface. These have been widely used for decades and are reliable and simple to use. Currently, the two most widely used methods are the Non‐Steady‐State Through‐Flow System (NSS_TFS, also called the closed dynamic chamber) and the Non‐Steady‐State Non‐Through‐Flow closed system (NSS_NTFS, or closed static chamber). Both types have a lid and are open to the soil surface, located on a collar fixed into the soil to maintain an airtight seal. The dynamic chamber is capable of higher frequency as air is circulated constantly between the chamber headspace and the analyzer; however, it requires an operator and a power supply. Static chambers are preferred when only occasional measurements are required. N_2_O and CH_4_ fluxes are normally measured by collecting gas samples to be analyzed later in the laboratory through gas‐chromatography, while CO_2_ is often measured using an IRGA (Infrared Gas Analyser). If only heterotrophic soil respiration is to be measured, autotrophic fluxes from roots and the rhizosphere must be excluded by inserting a cylinder deep into the soil well before sampling starts. Chambers need regular maintenance and calibration. Weather, fertilization, tillage, soil poaching, and harvest all influence emission levels, and when resources are limited, sampling should be more frequent around potential flux peaks, for example, rainfall, snow melt, litterfall, and agricultural activities.

## 4 Discussion

Large‐scale ecosystem research requires data that can be linked across sites in order to better understand earth system processes (Guo & Lin, 2016). First of all, the data must be findable and freely available (if necessary, after an embargo time). Thus Open Science, Open Data and Open Access initiatives are being promoted and supported by the European Community, national funding agencies and research organizations like PEER (http://www.peer.eu/). Then, either data are aggregated up to the level until they become comparable, usually resulting in a loss of much of the original information, or data are acquired following the same protocols. This can be achieved most easily where the protocols are developed using a top‐down approach, for example, the ICP Forest community regularly revise and disseminate protocol manuals (ICP Forests 2016) or the highly formalized protocols of NEON (NEON 2015). However, this top‐down approach works best if there is a central funding for data collection, otherwise many scientists would rather maintain a high quality time series than change methods for the sake of improved comparability across space.

These protocols offer an alternative approach that is lower cost, user‐led, and linked to the potential scientific benefits of data integration. Six LTER sites in Israel started using these protocols in 2016, and their use is being encouraged by the European LTER network, while the European H2020 project eLTER includes training sessions for scientists from LTER stations. The scientific benefits from this integration are starting to appear; there are several examples of these protocols being used across multiple sites to elucidate particular ecosystem processes (Cornelissen, 1996; Gardi et al., 2008), and more integrated methods of assessing ecological integrity have been developed (Capmourteres & Anand, 2016). We believe that these protocols will help create an ecological database that will enable much richer ecosystem models, able to support global, regional and site‐based decision making.

## Authors’ Contribution

Firbank is lead author and led on land use; Bertora, Grignani, and Delle Vedove led on GHG emissions; Blankman led metadata procedures; Frenzel led protocol prioritization; Groner and Menta co‐led on soil mesofauna; Matteucci and Kertész co‐led biomass and LAI protocols; Stadler and Frenzel co‐led on decomposition. Krab and Matteucci led training courses. Kunin was overall project leader. All co‐authors contributed to the writing; Krab co‐wrote Introduction and Discussion.

## Conflict of Interest

We report no conflicts of interest.
