# Supplementary material for: Towards the co‐ordination of terrestrial ecosystem protocols across European research infrastructures
Source: Ecol Evol. 2017 Apr 23;7(11):3967–75. doi: 10.1002/ece3.2997 (PMC5468142; doi:10.1002/ece3.2997)
Supplement: Supplementary file 1 [file ECE3-7-3967-s001.pdf]

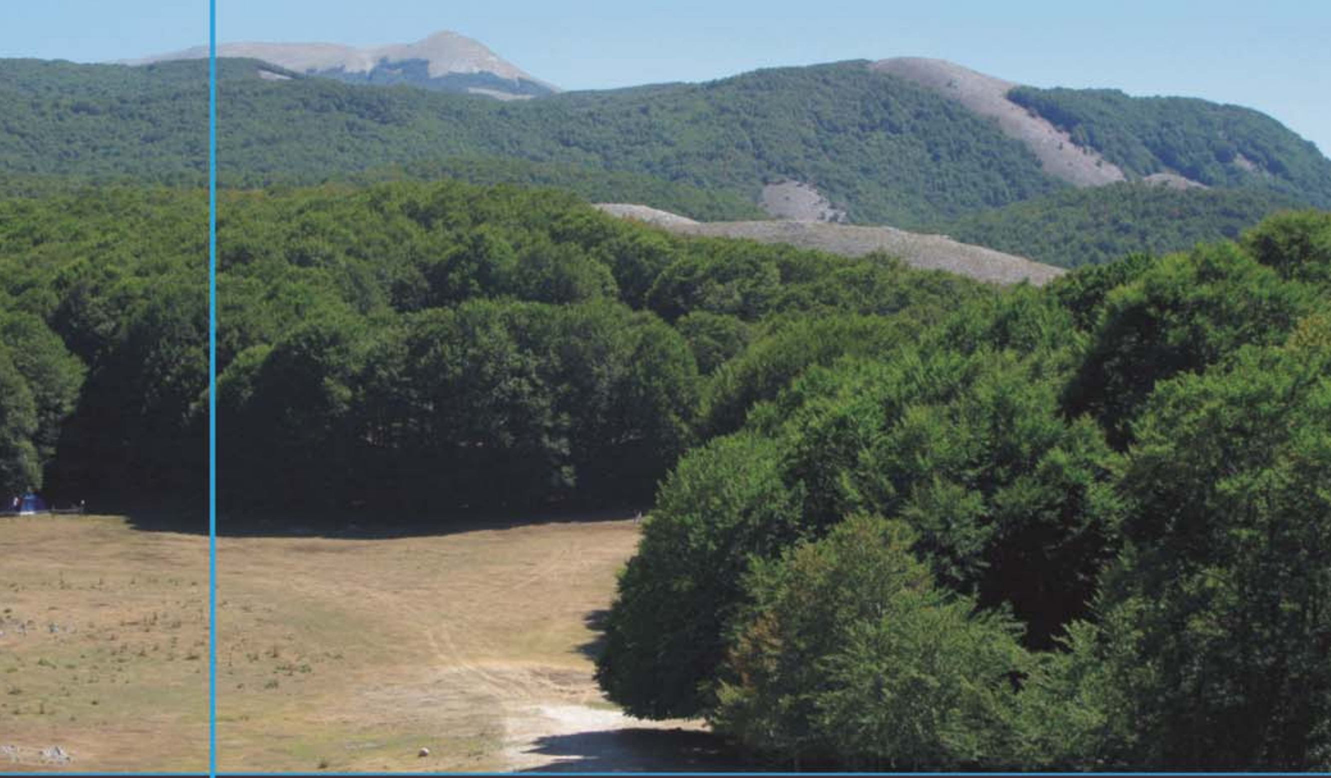

Handbook of standardised ecosystem protocols

Infrastructures for Experimentation in Ecosystem Research

ExpeER

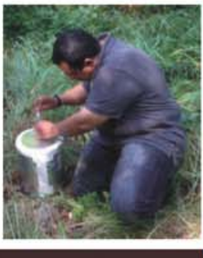
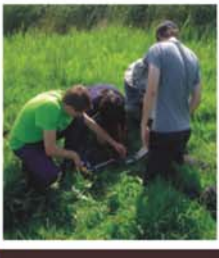
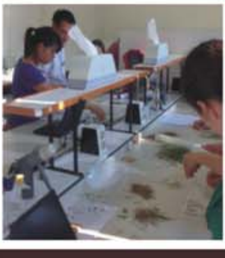
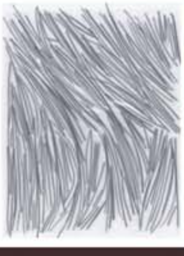
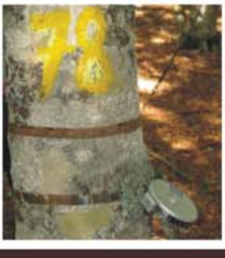

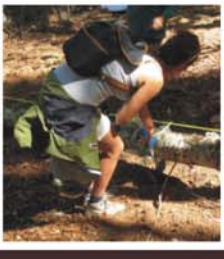

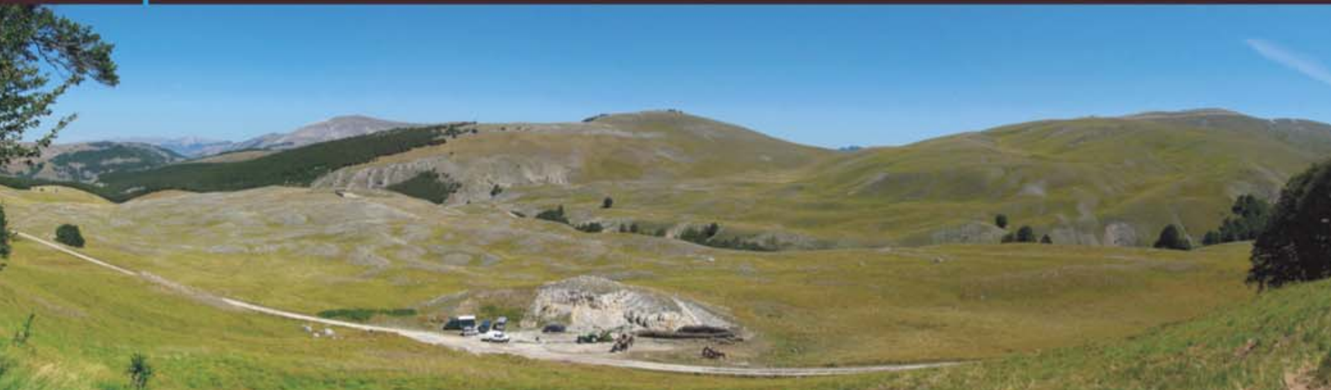

Handbook of standardised ecosystem protocols

ExpeER

Infrastructures for Experimentation in Ecosystem Research

The Manual: "Handbook for Standarised Ecosystem Protocols"
is the deliverable No D.2.3 of the FP 7 Project:

Distributed Infrastructure for Experimentation in Ecosystem Research (ExpeER)

Grant Agreement Number: 262060

Graphic design: Kinga Krauze; Layout: Krzysztof Kardasiński

TABLE OF CONTENTS

LIST OF AUTHORS 5

INTRODUCTION 7
Les Firbank, Mark Frenzel, David Blankman and Bill Kunin

ABOVE-GROUND PLANT BIOMASS 13
Giorgio Matteucci and Miklós Kertész

DECOMPOSITION 21
Jutta Stadler and Mark Frenzel

LAND USE AND MANAGEMENT 29
Les Firbank

LEAF AREA INDEX 37
Giorgio Matteucci and Miklós Kertész

SOIL MACROFAUNAL DIVERSITY 53
Amélie Joseph, Elli Groner and Cristina Menta

SOIL ORGANIC MATTER – CARBON AND NITROGEN STOCKS 61
Carsten W. Müller

GREENHOUSE GASES EMISSIONS FROM SOILS 65
Gemini Delle Vedove, Carlo Grignani and Chiara Bertora

ExpeER course on standarised ecosystem protocols,
Italy 2012

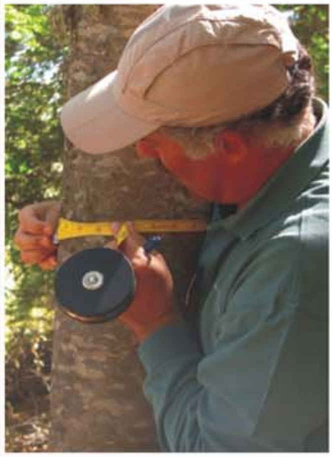

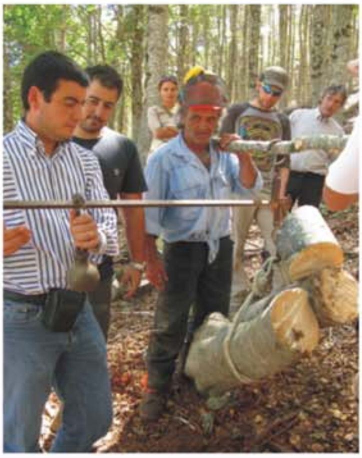

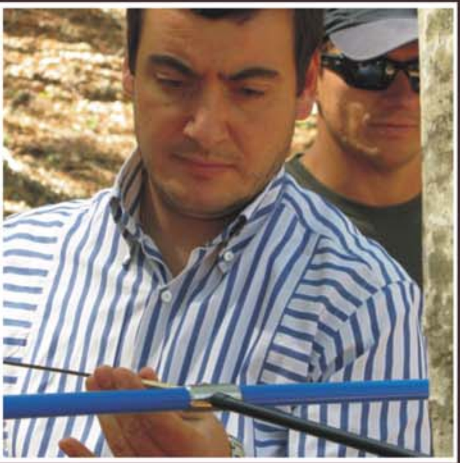

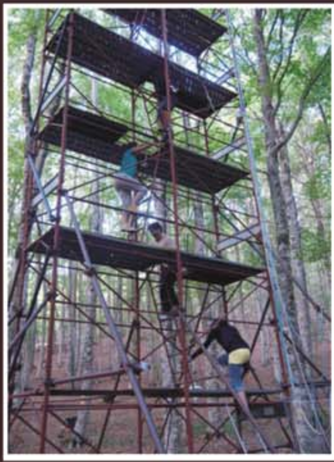

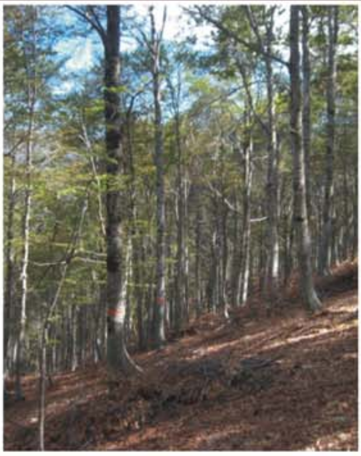

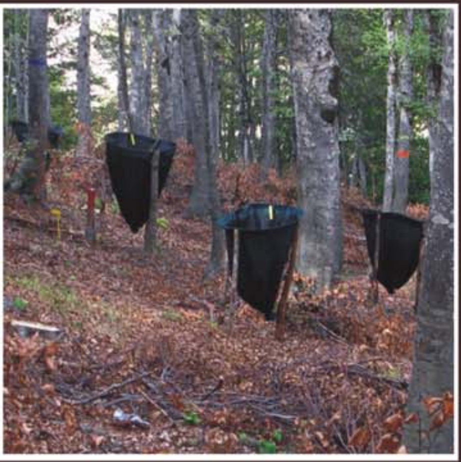

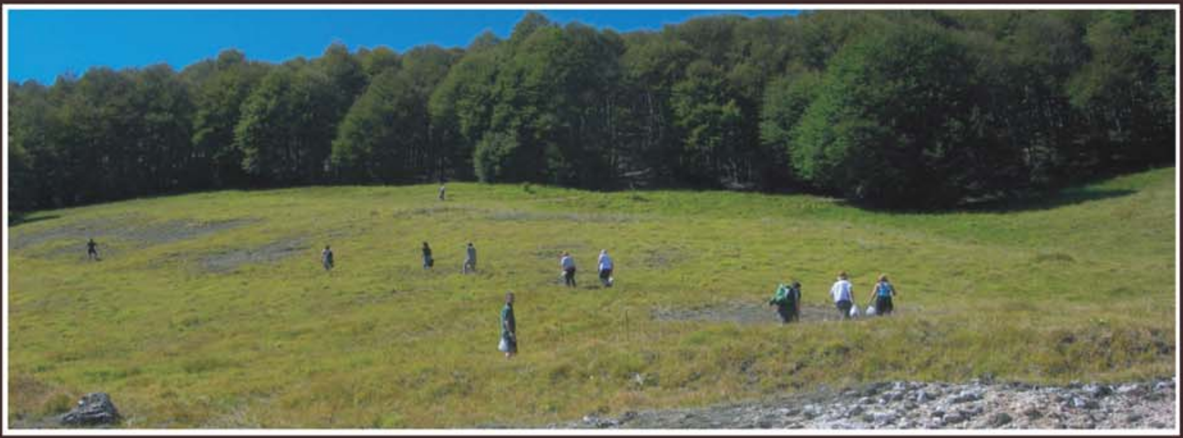

ExpeER

LIST OF AUTHORS

**Chiara Bertora**	Department of Agriculture, Forestry and Food Science,<br>Università degli Studi di Torino, Italy<br>chiara.bertora@unito.it
**David Blankman**	Director, Information Management, Israel LTER<br>Ben-Gurion University of the Negev, Beersheba,<br>Israel<br>dblankman1@gmail.com
**Gemini Delle Vedove**	Universita' degli Studi di Udine,<br>Dip.to di Scienze Agrarie ed Ambientali<br>Via delle Scienze 208, 33100 Udine, Italy<br>gemini@uniud.it
**Les Firbank**	School of Biology, University of Leeds, LS2 9RT,<br>Leeds, UK<br>L.firbank@leeds.ac.uk
**Mark Frenzel**	Helmholtz Centre for Environmental Research – UFZ,<br>Dept. Community Ecology, Theodor-Lieser-Str. 4,<br>D-06120 Halle/Saale, Germany<br>mark.frenzel@ufz.de
**Carlo Grignani**	Department of Agriculture, Forestry and Food Science,<br>Università degli Studi di Torino, Italy<br>carlo.grignani@unito.it
**Elli Groner**	Director of the Arava Center for Long Term<br>Socio-Ecological Research, The Arava Institute<br>for Environmental Studies, Kibbutz Ketura,<br>D.N. Hevel Eilot 88840, Israel<br>egroner@bgu.ac.il

**Amélie Joseph**	The Arava Institute for Environmental Studies,<br>Kibbutz Ketura, D.N. Hevel Eilot 88840, Israel<br>amelie.joseph.2@gmail.com
**Miklós Kertész**	MTA Centre for Ecological Research, Institute of Ecology and Botany, Alkotmány u. 2-4, Vácrátót, 2163 Hungary<br>kertesz.miklos@okologia.mta.hu
**Bill Kunin**	School of Biology, University of Leeds, LS2 9RT,<br>Leeds, UK<br>w.e.kunin@leeds.ac.uk
**Giorgio Matteucci**	CNR IBAF, National Research Council of Italy<br>Institute of Agroenvironmental and Forest Biology<br>Via Salaria, I-00015 Monterotondo Scalo (RM), Italy<br>giorgio.matteucci@cnr.it
**Cristina Menta**	Department of Life Science, Parma University,<br>via Farini 90-43121 Parma, Italy<br>cristina.menta@unipr.it
**Carsten W. Müller**	Lehrstuhl fuer Bodenkunde, Research Department Ecology and Ecosystem Management, Technische Universitaet Muenchen, Germany<br>carsten.mueller@wzw.tum.de
**Jutta Stadler**	Helmholtz Centre for Environmental Research,<br>Dept. Community Ecology, Theodor-Lieser-Str. 4,<br>D-06120 Halle/Saale, Germany<br>jutta.stadler@ufz.de

INTRODUCTION

LES FIRBANK, MARK FRENZEL, DAVID BLANKMAN AND BILL KUNIN

THE EXPEER PROJECT[1]

ExpeER (Experimentation in Ecosystem Research) is a European infrastructure project (2010-2014), which aims to bring together the major observational, experimental, analytical and modeling facilities in ecosystem science in Europe. By uniting these highly instrumented ecosystem research facilities under the same umbrella, and with a common vision, ExpeER is beginning to structure the very fragmented research community on terrestrial ecosystems within the European research area, improving the quality and the performance of these infrastructure components in a durable and sustainable manner. The ecosystem infrastructure within ExpeER will enable integrated studies to forecast the impacts of climate change, land use change and biodiversity loss on terrestrial ecosystem processes. The infrastructure will also help to integrate research and monitoring from Europe with that in the rest of the world. This integration involves two major steps:

- Building the ExpeER Integrated Infrastructure enabling collaboration and integration of observational, experimental and modelling approaches in ecosystem research;
- Using the ExpeER integrated infrastructure to structure the existing network of ecosystem observational and monitoring sites across Europe.

ExpeER provides the European research community on terrestrial ecosystems with state-of-the art, highly instrumented experimental (HIES) and highly-instrumented observational (HIOS) sites, where:

- the relevant ecosystem processes will be analysed simultaneously;
- their coupling within ecosystem functions through cascades of interactions and feed-back loops will be studied;
- the integration of ecosystem functions and determining ecosystem services will be achieve through a system biology approach, and
- the relation of ecosystem functions and services to biodiversity can be studied.

[1] http://www.expeeronline.eu/

ExpeER brings together long-term integrated experimental facilities allowing simultaneous measurements of key ecosystem variables and parameters through a multidisciplinary approach (biogeochemistry, soil microbiology, atmospheric chemistry, hydrology, agronomy, forestry etc.), to develop a more comprehensive approach to understanding terrestrial ecosystem functions and services.

THE STANDARDIZATION AND DISSEMINATION OF CORE VARIABLES AND PROTOCOLS FOR EUROPEAN ECOSYSTEM RESEARCH

The compilation and comparison of research findings across European ecosystem research facilities is often hampered by non-harmonised and non-standard measurement protocols that limit the comparability of datasets. Currently, while each research site may be able to show strong evidence of ecosystem process shifts at local level, it is difficult to draw conclusions about the generality of such shifts, or to sum effects across space. ExpeER takes major steps towards addressing this problem, both within the ExpeER network and ultimately among the broader ecosystem research community.

As an ecosystem develops, its ability to self-organize improves, and certain characteristics of the ecosystem evolve, such as primary productivity, foodweb connectivity and species diversity. To represent the ecosystem, a set of parameters is needed that describe both the ecosystem structures (biodiversity and heterogeneity) and the ecosystem processes (energy, water and matter fluxes). This set is termed the ‘ecological integrity framework’. This framework has been used to guide the choice of protocols within two closely related European projects, ExpeER and EnvEurope[2].

One of the primary objectives of ExpeER is to harmonize measurement and sampling methods for a core set of environmental and ecosystem variables across the focal network of participating research sites, so as to allow findings to be compared and generalised. The history of the search for a standardized list of ecosystem parameters goes back for almost a decade. The initial idea was to use a bottom-up approach that examines what parameters are commonly monitored in research sites across Europe. This has now been replaced by an approach to come up with a list of parameters that are important to indicate the state of ecosystems. The requirements for indicators are that they are easily measurable, indicative, clear, sensitive and provide useful early warning of damaging change. There are many projects examining indices of biodiversity, sustainability and environment in attempts to cluster a few parameters to one that indicates the state of ecosystems. EnvEurope has developed a long list of recommended parameters to be measured across Europe, along with methodologies[3] (http://www.enveurope.eu/). Sets of parameters are also being developed in other parts of world, notably within the US National Ecosystem Observation Network (NEON)[4].

---

2 http://www.enveurope.eu/

3 http://www.enveurope.eu/misc/PD_A2.1.2ab_Frenzel_et_al-ManualHarmonisedMethods_Rev2_0.pdf

4 http://www.neoninc.org/documents/513

A first set of ecosystem protocols for use across Europe is detailed within this handbook. Its development has involved:

- Selection and standardisation of a set of core parameters/variables;
- Development of a hierarchical set of common measurement protocols and standards intended for *in vitro* ecotrons, *in situ* experimental platforms and *in situ* highly instrumented monitoring observational sites, as well as recommendations for less instrumented sites in order to secure high quality reference data;
- Training, internal communication and dissemination activities to promote these common measurement methods (and other research protocols) and standards across sites;
- Maintaining communication with ongoing case study projects and using feedback to further optimise measurement protocols before disseminating outside the ExpeER network.

The approach has been iterative and hierarchical. Draft parameter lists and measurement protocols were generated, using a hierarchical logical structure and a nested set of technical requirements. These draft protocols were tested at a training course intended for ExpeER staff, before being refined and used for two externally-facing training courses during 2013.

HOW THE PARAMETERS WERE CHOSEN

For ExpeER, the goal was to choose a list that could serve as a pilot for establishing a set of parameters with standardized protocols, to be applied across many ecosystem research infrastructures across Europe. If this set of protocols wins support by contributing to our understanding of ecological change at the continental scale, it will encourage site managers to adopt more parameters with standardized protocols and more initiatives like this would be launched.

The parameters were chosen according to the following criteria:

- Considered important to ecosystem integrity;
- Common to many ecosystem research sites;
- Protocols are of an intermediate complication level;
- Protocols are easily executed and not too expensive;
- The parameters cover a variety of areas within terrestrial ecosystems.

We did not focus on parameters which are already highly standardised. For example, developing a protocol for measuring precipitation would be a waste of time, consistent methods are already generally used.

The search for the parameters started with the launch of ExpeER in January 2011, taking into account information about which parameters were already being measured at ExpeER sites. ExpeER members were consulted by email to develop first a long list of parameters, and then this list was prioritised. This list was brought to the ExpeER meeting at Leipzig in February 2012, which considered the list against the above criteria and chose a list of 10 parameters. Between March – June 2012 the person in charge of each

parameter developed the first draft protocol, using additional expertise as required. These draft protocols were trialled during a training programme, “TEsting and Refining SAmpling Protocols for Ecosystem Research” TERESA-PER, held at CNR, Rome on 27-31 August 2012. This course was aimed to protocol development, and so was targeted at ExpeER staff. In 2013, two training courses were held, which were targeted at non-ExpeER staff, at CNR – Rome, 20-24 May, and VU University – Amsterdam, 26-30 August.

The set of protocols was revised between these courses in the light of feedback from both members of ExpeER and delegates at the training courses.

Table 1. Final set of protocols

Ecological integrity indicators	Protocol
Matter storage	Above ground biomass
Matter loss, nutrient cycling	Decomposition
Habitat diversity, habitat management	Land use and management
Energy capture	Leaf area index
Faunal diversity	Soil macrofaunal diversity
Matter storage, element concentration	Soil organic matter – C & N stocks
Metabolic efficiency	Greenhouse gas emissions from soils

Use of the protocols is voluntary. Some include suggestions as to the frequency, timing and location of sampling, but these are not mandatory. Moreover, some of the protocols offer choices of methods, according to local situation and resources. The use of the protocols is very much driven by local needs and opportunities, along with larger scale projects and programmes.

RECORDING METADATA

In order to be used by other scientists, both now and in the future, it is essential that ExpeER datasets are documented according to appropriate metadata standards. These have been developed by the EnvEurope and ExpeER projects, and must be entered in the DEIMS website, at:

Production site: http://data.lter-europe.net/deims/

DEIMS provides several types of metadata. The three most important are: dataset metadata, site information and person. While a dataset can be considered to be one or more data entities (text file, spreadsheet, database view, or data service), it is most commonly a description of a single data entity. Required fields are marked with: *

1. Log in to DEIMS;
2. Select CREATE DATASET METADATA from the METADATA editor menu;
3. Enter appropriate TITLE for data – the title should be descriptive and meaningful to a broad audience. A good title would be similar to a title for a journal article.

4. Site name – begin typing in a site name. A list of possibilities will appear. Choose the correct one. If you are doing research at a location that is not connected to an ExpeER or LTER-Europe site, then a site metadata record will need to be created. The expeer.trainee user has the permission to do this on the training site, but not on the production site. If you are going to be entering information on the production site, contact David Blankman (dblankman1@gmail.com) for assistance.
5. Dataset contact names, owner etc. (these are references to PERSONs, who have already been entered. Begin by typing the last name. A list will appear, select the person from that list. If the person is not in the list, you will need to create a PERSON using the METADATA EDITOR/Create PERSON metadata menu item. When creating a person, if they are part of an ILTER network, choose the appropriate network. If the person is not part of an ILTER network, then choose the appropriate network from the Networks (in addition to ILTER) item.
6. Metadata date (this date defaults to the date of original entry).
7. Dataset Publication Date – this is a date, when the data was made available to a particular project, such as ExpeER or EnvEurope, or published in some other context. This is an optional field.
8. Dataset language – usually English.
9. Dataset abstract – description of the research. This entry is similar to an abstract in a journal article.
10. Keywords – select, at least, one keyword from the EnvThes Keyword field. Enter as many keywords as are appropriate for the dataset. They include EUNIS habitat lists.
11. Dataset access and use constraints – metadata are expected to be public, read, data owner, all. Use this field to indicate access to the data that is being documented. There are several categories: Administrative/Governmental, Education, Research, Public, LTER-Europe, ExpeER.
12. Intellectual Rights, for example, right to review any results based on the data. Choose one or more as appropriate.
13. Dataset online distribution – how can people get at the raw data. Please choose whether the web page referenced contains the actual data or provides instructions on how to access the data. This is a required field. If there is no online resource for this data, write NONE.
14. Dataset location – one can input location from the map, including a bounding box.
15. Altitudes – upper and lower.
16. Temporal extent – start and end dates.
17. Taxonomic coverage – if relevant. Provides information about the taxonomic classification of the organisms represented in the dataset. This element has two components: class, family, order, etc., and mammalia, carnivora, Felidae. This field is applicable only for biotic data. Depending on the content of the dataset, provide information about the most common level of taxonomy aggregation (plants: family, marine invertebrates: phylum or class, etc.) Recommendation is to use common catalogue of species, for example, Catalogue of Life, GBIF, or EUNIS.

18. Dataset methods description – reference a published protocol, if a web address (URL) is available. In addition provide a description of the method used. Please pay special attention to indicating any differences from the published protocol.
19. Instrumentation – where required. Provides information about any instruments used in the data collection or quality control and quality assurance. The description should include vendor, model number, and optional equipment.
20. Sampling description – where required. Allows for a text-based/human readable description of the actual sampling procedures used within the dataset collection. This element shall include information about dataset lineage – general explanation of the data producer's knowledge about the lineage of a dataset. This element shall also provide a description or geographical definition of the representative area of sampling.
21. Legal obligation reporting – provides information whether a dataset has been reported to the local, or regional, or national bodies to fulfil the obligations from particular legal regulations. Probably will not be applicable. Choose the directive if it is applicable.

This represents a basic set of instructions for providing dataset metadata using the Community profile. For further details, see the video tutorial on:

http://vimeo.com/60479680

It is highly recommended that additional metadata be provided using the EML Data File and EML Variable forms.

ABOVE-GROUND PLANT BIOMASS

Giorgio Matteucci and Miklós Kertész

ECOLOGICAL INTEGRITY INDICATOR

Biomass – Energy storage (in biomass).

MEASURABLE

Above-ground biomass of terrestrial vegetation.

PROTOCOL SUMMARY

This protocol is related to the assessment of biomass of vegetation in forests and grasslands/croplands, perhaps the most important "state" variable for characterising terrestrial ecosystems. Biomass is here defined as the total amount of living organic material (excluding litter, soil organic matter, deadwood) standing over a unit ground surface area (m^2, ha). Biomass has two main fractions: aboveground (stem, branches, foliage) and belowground (fine and coarse roots). In forests, tree stumps are considered at the "edge" between aboveground and belowground biomass. Biomass of annual plant species is equal to Net Primary Production (NPP), excluding possible loss of mass between plant emergence and the date of sampling. Assessing belowground biomass is difficult in forests, while it is easier in grasslands and croplands, where root systems can be harvested together with aboveground part, albeit with some soil disturbance.

Plant biomass can only be measured directly destructively. Therefore, for trees and shrubs, biomass is typically estimated indirectly from an allometric relationship obtained from a sample of plants (trees or shrubs), relating a parameter that is measurable on living plants (diameter at a certain height, e.g. breast height for trees, or diameter and plant height) and biomass. Afterwards, total biomass can be estimated by applying the allometric relationship on the diameter distribution of plants assessed in the field. Such relationships are species- and often site-specific. Biomass estimations from surveys repeated, e.g. every 3-5 years will provide trends in biomass growth and hence mean NPP. Direct measurements of biomass give opportunities to measure leaf area index.

Biomass estimates for grasses and herbs are best sampled directly using clipping at the time of yearly maximum above-ground plant material.

KEYWORDS

Allometric relationships, biomass, destructive sampling, net ecosystem production, net primary production.

SCIENCE BACKGROUND

Knowledge on the key parameters of carbon cycle is essential in understanding of the function of ecosystems. However, the measurement of those parameters, especially in terrestrial ecosystems, is very cost and labour intensive, thus, a combination of different measurements, including direct measurements, measurements on proxy variables, and calibrations are applied for estimating of the key variables. In a given terrestrial biome, the living plant biomass is strongly correlated with leaf-area index (LAI). LAI is extensively estimated by means remote sensing. The Annual Net Primary Production (ANPP) can be estimated using the yearly pattern of LAI. Thus, direct measurements of yearly maximum living plant biomass and LAI, give opportunity to apply a broad spectrum of known correlations, as well as crop production simulation results, for estimating ANPP. In the case of the temperate steppe biome, under certain conditions (unimodal yearly growth pattern of living plant biomass, regular removal of plant biomass after the yearly peak), maximum yearly plant biomass is an appropriate estimator of annual net primary production (ANPP).

METHODOLOGIES

Two methodologies are given, one for trees in forests, based upon a protocol developed within the EU project CANIF (Scarascia Mugnozza et al. 2000), and one for biomass of grasslands, based on Milner and Elfyn Hughes (1968). Both have been widely used (e.g. Scarascia Mugnozza et al. 2000; Bascietto, 2004). In forests and shrublands, biomass estimations from surveys repeated time by time (e.g. every 3-5 years) will provide trend in biomass growth, and differences will provide mean Net Primary Production (NPP).

Aboveground biomass in forests

The measureable is the total amount of living organic material (excluding litter, soil organic matter, deadwood, that are usually assessed separately) standing over a unit ground surface area (m^2, ha). Biomass has two main fractions: aboveground (stem, branches, foliage) and belowground (fine and coarse roots). In forest, stump is considered at the “edge” between aboveground and belowground biomass.

In forests, the approach presented within ExpeER is two stage:

1. Building allometric relationships between biomass and a parameter that is easily measured on living trees in the field (tree diameter or diameter and height).
2. Estimating total biomass by applying the allometric relationships on the parameter distribution assessed in the field.

The protocol includes:
1. How to select trees within a population;
2. Harvesting and weighing trees;
3. Building an allometric relationship.

Sampling of tree aboveground biomass

1. Sample frequency:
   a) An allometric relationship can be determined even with just one set of data. The relationship(s) can be expanded with sampling in following years, to make it (them) more and more precise. Data of both biomass of single components, along with diameter, and other measured variables of the sampled trees are useful to allow calculation in the future.
2. Selection of trees within the population:
   a) One large (> 2700 m^2) or more, preferably at least three smaller (~ 1200 m^2) sample area is surveyed for tree diameters, tree species, tree height (the latter even on a subsample of trees);
   b) The size of the survey area is to be selected in relation to the stand characteristics (generally smaller for denser and more uniform forest). Diameter of the area can range from 25 to 40 m;
   c) Data are analysed and a diameter vs. height curve is produced;
   d) Trees to be harvested to build the allometric relationship are selected within the population so that they represent the range of variability of the stand.
3. Harvesting of the selected tree(s):
   a) Measurement to be taken before felling the selected tree:
      – diameter at breast height (1.3 m), crown projection (4 radius from the stem, N-E-S-W), general crown shape, height;
   b) Measurement to be taken when the tree has been felled:
      – Length of the tree (=height); height of crown insertion (the first "important" green branch) and diameter at that point.
4. Assessing biomass:
   As a general rule, it is better to weigh all that is possible (the whole tree and crown, separated in section/portion).
   In case of very large trees, sub-sampling may be required:
   a) Crown:
      – Crown can be sampled "all together" or according to three portion of approx. equal length, starting from the height of crown insertion. Usually in small trees crown is sampled all together, in larger trees can be sampled by cutting the trunk into portions:
         • if sampled in portion, record the following: diameter at the base and at the top of the portion, length of the portion;
         • branches of each portion are collected separately and weighed. In case of very large crown, sample branches can be used instead of weighing all the branches;

– After that, start the "cut and weight" operations from the crown and leave the stem at last, as the former is more subject to possible water losses (particularly for leaves).
  For the whole crown or for each of the crown portion:
  - cut and collect all branches, dividing them into diameter classes if they are a large number;
  - weigh branches all together or in diameter class;
  - if there are a lot of branches, select a number of sample branches in each diameter class. The number of sample branches can vary according to the total number of branches in the diameter class (normally 3-5). The sample branches will be used to calculate allometric relations that will be used to estimate total leaves, twigs and branches biomass of each class;
  - if foliage is present, separate foliage and twigs from the branches and weigh all the components;
  - prepare a sample to determine the dry/fresh weight ratio to calculate dry biomass of branches, twigs and leaves; the sample can be used also for nutrient analysis;
  - on some of the sample branches, a cross section can be cut at the base. The section can be used to calculate branch NPP in the lab;
  - if Leaf Area is also of interest, a sub-sample of fresh foliage can be used to Specific Leaf Area ($m^2 g^{-1}$) or Leaf Mass per Area ($g m^{-2}$). This parameter, multiplied by the total weight of foliage will result in the total leaf area of that tree. See Leaf Area Index protocol.

b) Stem:
– After the crown is sampled, the remaining part is a "clean" stem; the suggestion is to consider it as "stem" from the tree base to the real top, without separating it according to diameter in classical forester "commercial" classes; the latter log will be then the "top log".
  - If the stem is longer (= higher) that 10-12 m, separate it into 2-m-long logs, if it is shorter, the logs can be 1-m-long;
  - for each log: measure base, central and top diameter;
  - ideally, weigh all logs or, if it is too demanding, weigh one log out of two, alternating even and odd logs (butt log = 1) in successive sampled trees (tree #1, log 1-3-5-7-...; tree #2, log 2-4-6-8-...);
  - cut a 2-3 cm thick cross section at the base of each log and mark its bottom face; these sections will be brought back to the lab for dendrological analysis and for calculating the biomass/volume ratios; some of the cross sections will also be used to determine the bark to stem ratio;
  - always cut and bring back to the lab the cross sections at 1.3 m and at the base of the crown.

Notes:
– Trees in the lower diameter classes (up to 5-10 cm) can be sampled complete, without forming sub-samples.

The trees selected for harvest should be measured, felled and weighed tree by tree, otherwise there is the possibility of introducing errors (weight loss, possible of confounding samples, etc.).

c) Laboratory operation:
   - Volume of stem (and branches if any) cross sections (disks) has to be evaluated on fresh samples;
   - Subsamples must be weighed again fresh and then dried to assess the dry/fresh weight ratio;
   - Dry biomass should be assessed at 105°C. Check for constant weight during drying. Nutrient content should be determined on samples that have been dried at 65-70°C, to prevent nitrogen loss and then referred to dry weight at 105°C.

   Notes:
   That the volume of cross sections can be also determined with sufficient precision by submerging just under the (distilled) water surface, at room temperature (i.e. around 20°C) a section on a container placed on a balance. At room temperature, the weight of displaced water will be equal to the section volume (i.e. 1 cm^3 = 1 g).

Assessing biomass at stand level

1. Sampling size:
   a) As usual, the larger the better. An affordable sampling size to start with could be from 5 to 10 trees, according to population variability. The trees have to be selected according to diameter classes (average, can change according to forest structure); some more trees can be sampled in the lower diameter classes (up to 5-7 cm), if these are relevant in the forest structure.
   b) Consider that the effort can then be "enlarged" in other years, given the fact that an allometric relationship would then hold for the stand/site almost forever (if species does not change).
2. Calculating the allometric relationship:
   a) The measured basic variable (usually the diameter) is then correlated to the biomass of different components (branches, stem, foliage) and, if useful and needed, to the total biomass.
   b) Usually the equation is in the following form:

$$\mathrm{Biom}_{(\mathrm{component})} = a \bullet \mathrm{Diam}_{(\mathrm{tree})}{}^{b}$$

   With a and b parameters assessed by statistics/fitting.

Aboveground biomass in grassland and cropland

The metadata should consist of geographical coordinates, elevation, exposition, habitat type, and sampling scheme, including number, size, and arrangement of sampling units. The specific measurables are as follows:

Yearly maximum of aboveground biomass of vascular plant

Measurements of the aboveground biomass of mosses and lichens are optional. In case of woody plants (shrubs, dwarf shrubs, vines), the biomass of the offshoots of the last growing season should be measured. Yearly maximum aboveground biomass data for each vascular species separately are optional.

Site selection

The criteria of site selection are as follows:

- *Vegetation.* The vegetation should be dominated by soft-stemmed vascular plants of less than 2 m height.
- *Long-term security.* The treatments of the sites should be as permanent and predictable at long term at possible.
- *Homogeneity.* As the sampling units are relatively small (0.5 × 0.5 to 1 × 1 m^2), the biomass and LAI of the studied plots should be homogeneous enough to be reliably sampled by a few sampling units.
- *Representativity.* It is desirable that the sites represent areas which are characteristic to the region and consist of patches large enough to provide reliable remotely sensed data.

Method

- *Sampling units and design*. Square shaped sampling units are used, from 0.5 × 0.5 m^2 to 1 by 1 m^2. The number and arrangement of sampling units have to be reliable estimation of biomass and LAI of the plot. Depending on the heterogeneity of the grassland, application of one to five sampling units is suggested per plots.
- *Timing*. In grassland mown once a year, sampling should be made right before mowing. Otherwise, or in case of multiple mowing, the sampling should be made once a year, at the time of the maximum LAI.
- *Clipping*. As much as possible, clipping should be made right at the level of the ground. The old bunches of grasses may form small mounds of dead plant material and soil; that part should be left on the ground. In case of wetlands, the ground can be a soft net of mosses and roots. There the ground could be determined by the lowest level of seemingly green plant material. The woody parts of plants, which are seemingly older than one year, may be left in the sampling units.
- *Drying*. All plant material should be dry at 60°C until constant weight. If this is impossible to achieve, drying at room temperature in well ventilated dry room until constant weight is satisfactory.
- *Separation into fractions*. If it is possible, separation must be done while the plant material is still wet. It is often impossible, thus the dry plant material is separated; in this case, the status of plant material should be extrapolated back to the time of clipping. Three fractions should be formed: (1) plant material, which was dead in the time of clipping, (2) non-photosynthesising living material, and (3) photosynthesising material. The woody parts of the pants, which had apparently grown in

previous seasons, should be counted as dead material. If it is not clear whether a part of the material were photosynthesising or not at the time of clipping, it is better to count as photosynthesising part. Typically, only leaves are counted as photosynthesising parts, the otherwise green stems and inflorescences not. However, in case of certain plants, the stems provide the photosynthesising surfaces (e.g. the stems of *Equisetum* species); in these cases, the stems are also counted as photosynthesising parts. The plant material can also be separated by species or other groups.

- *Biomass*. Biomass is calculated by summing up the dry weight of all living material (fractions 2 and 3), and expressed in grams/m^2.

Data capture

The measurements should provide the following data for each sampling unit:

- Aboveground living vascular plant biomass, dry material grams/m^2;
- Leaf Area Index, m^2/m^2.

REFERENCES

Bascietto, M. 2004. *Direct Assessment of Aboveground Carbon Sequestration in Forests along a Latitudinal Gradient in Europe*. PhD Thesis, Università degliStudi della Tuscia (Viterbo, Italy).

Milner, C., Elfyn Hughes, R. 1968. *Methods of the Measurement of the Primary Production of Grasslands. IBP Handbook No. 6*. Blackwell, Oxford and Edinburgh.

Scarascia Mugnozza, G., Bauer, G., Persson, H., Matteucci, G., Masci, A. 2000. Tree biomass, growth and nutrient pools. In: E.-D. Schulze (ed.), *Carbon and Nitrogen Cycling in European forest Ecosystems*, *Ecological Studies 142*. Springer Verlag, Heidelberg. Pp. 49-62. ISBN 3-540-67239-7.

DECOMPOSITION

Jutta Stadler and Mark Frenzel

ECOLOGICAL INTEGRITY INDICATOR

Matter budget.

MEASURABLES

- Mass loss of standard litter substrates.
- Mass loss of bait.

PROTOCOL SUMMARY

Decomposition is among the most important biological drivers of the biogeochemical cycling of nutrients and carbon sequestration. Decomposition is influenced by many abiotic and biotic factors as e.g. soil temperature and moisture, soil chemistry, litter substrate quality, and soil fauna community composition. As soil faunal structure is very complex, the soil fauna feeding activity can be used as an indicator of the biological status of the soil. Both litter bags and bait lamina provide simple measures of the soil fauna feeding activity.

Litter bags filled with a standard litter substrate are a good choice, when comparing decomposer activities of different ecosystems or biogeographic regions. It is simplest to use a standard litter consisting of leaves of wheat, barley or maize leaves, as these species are easy to grow at all sites. Leaves of tree species are often used as standard litter, especially for experiments in forest ecosystems. Leaf litter can be from a single variety, monospecific or polyspecific and/or contain local natural or cultivated species or invasive/non-local species.

Litter bags, size 10 × 10 cm are put randomly in the field for several weeks or months to allow decomposition. A bag size of 10 by 10 cm gives a good balance between a reasonable amount of litter and decomposition turnover rate. Litter bags are filled with 2 g dried standard substrate. After removing from the field, litter bags are re-weighed.

The bait lamina method is a quick and inexpensive way of screening soil biotic activity. The consumption of bait by soil organisms is a proxy for the feeding activity of the soil fauna, complementing cumulative parameters such as decomposition rate or mass loss of standard litter. The bait lamina strip is a PVC-strip of 15 cm length, which has 16 conical holes at the lower 8 cm part. The conical holes are filled with a standard substrate mixture of fine ground cellulose powder, bran flakes and traces of active coal (ratio 70:27:3). Bait lamina strips are plunged in the soil with the uppermost located bait hole positioned short beneath the soil surface. The bait lamina strips are removed after exactly 14 days and each hole is classified in "bait eaten" or "bait not eaten". Soil invertebrates and, to a smaller extent, microorganisms progressively degrade the bait placed in the soil substrate in a very short time span.

KEYWORDS

Bait lamina, decomposition, litter bag, soil biodiversity, soil invertebrates, soil microorganisms.

SCIENCE BACKGROUND

Decomposition is among the most important biological drivers of carbon sequestration and nutrient cycling and an important ecosystem service. Although decomposition is influenced by climate, litter quality and decomposer species, it is a useful tool for a quick and general overview of soil biotic activity. Decomposers are of particular importance in climates, where moisture and temperature are not limiting. Furthermore, an increase in nutrient availability will increase decomposition rate. The set-up of a standardized protocol will help to investigate and compare decomposer activities of different biomes, ecosystems or ecoregions.

Litter bags as well as bait lamina, are a widely and long since used technique for receiving information about soil feeding activity. Nevertheless, the great variability in technical details makes a comparison of single studies impossible. The protocol ensures that data are collected with the same routine, and therefore enables a comparative monitoring within the EXPEER infrastructure.

Standard litter is a good choice to investigate decomposer activities of different ecosystem or biogeographic regions. Standard litter can be monospecific as well as polyspecific. The simplest way is to use grain leaves (wheat, maize or barley) as this is easy to cultivate at almost all sites. Nevertheless, to minimize site specific differences, standard substrate should be cultivated at a single site and distributed. Another advantage of grain as standard litter is, that litter quality can be varied easily by specific fertilizer treatment.

Bait lamina strips are a quick test for soil biotic activity (von Törne, 1990; Kratz, 1998). Plastic sticks with defined holes are filled with a bait material (finely ground grain and cellulose). The bait material can be adjusted to specific research questions by changing

the bait components (e.g. replacing part of the grain with finely ground plant material). The use of litter bags and/or bait lamina strip is a question of time of exposure and experimental design. The use of litter bags enables to test in more detail (e.g. by varying mesh size or litter quality), but it is more time consuming than using bait lamina strips. The hypothesis, which should be tested, determines the method to be used.

METHODOLOGY

Measurable

- Mass loss of standard litter substrates [g].
- Mass loss of bait [%].

Site selection

The standard litter as well as the bait lamina technique is applicable in all ecosystems (also aquatic ecosystems).

Experimental plots must be randomly distributed within a site. If different treatments as e.g. nutrient addition are tested, a random block design is recommended.

A plot size of 1 × 1 m^2 is a reasonable size to place a sufficient number of litter bags within a homogenous area. For each experimental treatment, a repeat of 5 plots is recommended to minimize variability. Litter bags are randomly distributed within one plot with at least 20 cm distance between bags.

The distance between plots is at minimum 1 m to avoid edge effects. The distance of this corridor may vary a bit (to a higher distance) to allow for compensation for unsuitable ground.

Bait-lamina strips (5 strips) for measuring the feeding activity of soil animals are placed randomly within each plot with at least 20 cm distance between the strips.

Litter bags as well as bait lamina test methods are a measure of general activity of soil biotic organisms. Their activity depends on numerous abiotic conditions (temperature, moisture, nutrients) and varies considerably throughout the vegetation period. Therefore, a reasonable amount of repeats is recommended. During the decomposition process, litter quality changes resulting in a change in litter decomposing organisms. A sampling at different times during the vegetation period allows, e.g. to test for changes in enzyme activity of the substrate.

General time schedule in short:

- Production of standard litter in the field (e.g. barley) in spring/early summer.
- Filling litter bags until mid of June; preparing bait lamina.
- Bringing out all litter bags and bait lamina until end of June.
- Sampling of bait lamina exactly after 14 days exposure.
- Sampling date of litter bags earliest 6 weeks after bringing them out. If several sampling dates are chosen, time between single sampling dates should be 6 to 8 weeks.

Materials and Methods

Litter bags

Litter bags with a different mesh size allow testing for activities of either macro-invertebrates or fungi and bacteria. Mesh sizes lower than 100 µm enable fungi and bacteria only to colonize the bag, while litter bags with a mesh size of 1mm and beyond allow also invertebrates to act as decomposers (Pye et al. 2012). A mesh size of 5 × 5 mm is permeable for microbes, meso- and macrofauna. A mesh size of 20 × 20 µm is small enough to allow access by bacteria, fungal hyphae, most nematodes and protozoa, while restricting access of meso- and macrofauna. A bag size of 10 by 10 cm turned out to be good balance between a reasonable amount of litter and decomposition turnover rate.

For best standardization, pre-manufactured bags shall be used. Usually bags have to be ordered in time (ideally ordered centrally), because often they are not in stock and manufacturing needs some time. Litter bags are filled with 2 g dried substrate. Leaves should not be damaged or already be colonized by fungi or pathogens. This amount of substrate is sufficient for reasonable results within a comparatively short time span. Litter bags are labelled inside by a piece of plastic, containing plot number, treatment, repeat, running number and initial dry weight. It is strongly recommended to label with a graphite pencil and add the label inside the bag before sealing. You may additionally label the bag outside on the adhesive tape, but be aware that this labelling often disappears during the exposure.

Litter bags are sealed with a strong adhesive tape. Only the coarse mesh can be additionally sealed with a stapler.

When litter bags are removed from the field, they need to be dried for 5 days at 60°C. After opening (be careful with the fine mesh as it easily breaks), the remaining substrate needs to be cleaned from dirt, moss, needles or any other parts which are not standard substrate and weighed. The remaining substrate will be stored in paper bags for further investigations (e.g. chemical analyses).

Placement of litter bags in the field:

- *Forest*: Try to avoid herbaceous cover as this makes it difficult to place the substrate bag. If you can´t avoid, place your bags between bulks of herbaceous species directly on the ground. It is not necessary to remove old and remaining litter as well as moss cover. Simply place your litter bag on the bottom and fix it with a wire cramp without pushing it through the fine mesh. If you puncture the fine mesh, macro-invertebrates can enter the bag and falsify the result!
- *Grassland:* If you place the substrate bag in a grassland site, which is regularly mown, please make sure that it is mown before you put out your bags. Mowing of the experimental plot before exposure is not mandatory, but can be done if this makes it easier for you to place the bags. Try to put litter bags between the bulbs and rhizomes of the plant species so that they have maximum contact to the soil. Fix the bag with a wire cramp but without pushing through the (fine) mesh. Instead of fixing each bag separately with a cramp, you may cover the whole plot with wire mesh to keep your bags tight to the ground.

Standard substrate

It is recommended to use standard substrate only. Sampling of site specific substrate has some constraints. It is very time consuming to gain the requested amount of site specific substrate. Furthermore, the varying quality of the substrate of different sites may interfere with other treatments and overlay results.

Barley is proposed as a standard substrate as this is easy to cultivate and collect, and avoids site specific constraints. It is not the species itself that is of interest, but the decomposition process between different sites and ecosystems (e.g. along a biogeographic gradient). Barley can be cultivated in sufficient amounts at one site and be distributed amongst partners with a very low financial or temporal investment.

Bait lamina strips

The bait lamina test method is a quick and inexpensive screening of soil biotic activity. Soil invertebrates and microorganisms progressively degrade the bait placed in the soil substrate in a very short time span. Although it is difficult to disentangle the effects of fauna and micro-organisms on feeding activity completely, recent studies have shown that the macro-organism are the main feeders on bait lamina (Simpson et al. 2012). Therefore it is assumed that the disappearance of the bait material reflects the feeding activity of soil invertebrates and only to negligible extent microbial processes. The standardization of the bait-lamina test allows comparing the feeding activity of soil organisms in e.g. different ecosystems or under different management treatment. Nevertheless, the catchment area of a bait lamina strip is very small. Therefore, a minimum number of 10 repeats per plot are required.

The bait lamina strip is a PVC-strip of 15 cm length, which has 16 conical holes at the lower 8 cm part. The conical holes are filled with a standard substrate mixture of fine ground cellulose powder, bran flakes and traces of active coal (ratio 70 : 27 : 3). This fine-grained powder is mixed with water to a paste and then filled into the conical holes. After filling strips are dried for 3 hours at 60°C. The filling and drying must be repeated 1-4 times, unless the holes are filled properly.

To compare different soil feeding activities, an evaluation of the activity in the upper soil sections seems sufficient. If the depth of the soil profile is rather low (e.g. in flat A-C-soils), feeding activity can be assessed by shorter bait-lamina strips. In any case, the uppermost located bait hole is positioned short beneath the soil surface. The bait lamina strip is a PVC-strip of 15 cm length, which has 16 conical holes at the lower 8 cm part. The conical holes are filled with a standard substrate mixture of fine ground cellulose powder, bran flakes and traces of active coal (ratio 70 : 27 : 3). This fine-grained powder is mixed with water to a paste and then filled into the conical holes. The strips get less damaged if they are inserted into prefabricated slits done, e.g. with a screw driver or knife. The tool used for this prefabrication should have the same size as the bait lamina strip to avoid a loose contact to the surrounding soil.

The bait-lamina strips are left in the soil/substrate until about 10-40% of the baits are perforated. Since the necessary exposure time depends on the site and on the moisture

content of the soil, feeding activity assessment can require between 7 (in soils with good moisture conditions) and 20 days (dryer soils) exposure. However, it is recommended to remove the bait laminas exactly after 14 days. This short exposure time of the bait lamina keeps the influence of micro-organisms small (Gongalskyet et al. 2004). This enables each site partner to adjust the sampling to his personal working schedule.

After retrieval, the strips are stored in PE-foil or PE-bags to preserve the baits from drying out and to prevent formation of cracks that could be interpreted as feeding holes.

The evaluation of the exposed baits is achieved after removing adhesive soil particles very carefully (e.g. with a soft brush). Afterwards the strips are examined on a lighted bench. Differentiation is made only between “bait eaten” (1) and “bait not eaten”(0). Feeding is rated only when light crosses at least punctually the bait, transparency alone is not sufficient. It needs to be discussed, whether the evaluation should be done by one person only to minimize variation in the estimates.

Bait-lamina tests can be performed at any given time, except in periods of ongoing dryness and/or ongoing soil frost. Lowest thermal limit for faunal activities in soils is approx. 4°C. Nevertheless, all sites should perform the bait lamina test in more or less the same time span during the vegetation period, as the feeding activity varies with abiotic conditions.

REFERENCES

Gongalsky, K., Pokarzhevskii, A., Filimonova, Z, Savin, F. 2004. Stratification and dynamics of bait-lamina perforation in three forest soils along a north-south gradient in Russia. *Applied Soil Ecology* 25(2): 111-122.

Kratz, W. 1998. The bait-lamina test: general aspects, applications and perspectives. *Environmental Science and Pollution Research* 5(2): 94-96.

Pye, M., Vaughan, I., Ormerod, S. 2012. Episodic acidification affects the breakdown and invertebrate colonization of oak litter. *Freshwater Biology* 57(11), 23-18-2329.

Simpson, J., Slade, E., Riutta, T., Taylor, M. 2012. Factors affecting soil fauna feeding activity in a fragmented lowland temperate deciduous woodland. PLoS ONE 7(1): e29616. Doi: 10.1371/journal.pone.0029616.

Von Törne, E. 1990. Asessing feeding activities of soil-living animals. 1. Bait-lamina-tests. *Pedobiologia* 34, 89-101.

SUMMARY INSTRUCTIONS

- Ecosystem: Terrestrial or aquatic.
- Location in plot: random.
- Litter substrate: 2 g dries standard litter substrate (e.g. barley, wheat) produced centrally in spring.
- Litter bags: Size 10 × 10 cm; mesh size 5 × 5 mm; 20 × 20 µm.
- Bags sealed with adhesive tape.

- Bags fixed in the field with cramps or wire.
- Removal of bags after 1.5, 3 and 6 month.
- Removed litter bags dried for 5 days/60°C, remaining litter weighed.

Bait lamina:
- Bait component: cellulose, bran flakes, and active coal in a ratio of 70 : 25 : 5; mixed with water to a paste; holes filled with paste several times to ensure a correct filling. Dry between the single filling procedures.
- Bait lamina strips are plunged in the soil with the uppermost located bait hole positioned short beneath the soil surface. Pre-drill the soil with an appropriate tool.
- Bait lamina are removed after 14 days exposure in the field.
- Classify in “bait eaten” or “bait not eaten”.

LAND USE AND MANAGEMENT

Les Firbank

ECOLOGICAL INTEGRITY INDICATOR

Habitat.

MEASURABLES

Land cover and habitat management.

PROTOCOL SUMMARY

This protocol captures the nature of the land and habitat that is present on an ecological study site, the purpose of management, and management records. This protocol therefore ensures that the site is considered appropriately in multi-site classifications, and that records of operations such as cultivation, sowing, thinning and harvesting are captured.

The site should be categorised into spatial units that are managed in the same way, e.g. an agricultural field, an even-aged stand of forest, a plot in a field experiment, a chamber in an ECOTRON. This protocol ensures that appropriate records for each spatial unit are kept in a consistent way, for access through the ExpeER metadatabase, to enable the appropriate analyses of data within and between sites.

KEYWORDS

Agricultural management, ecological context, ecological experiments, ecological metadata, forest management, habitat classification, land use, landscape, land management, vegetation classification.

SCIENCE BACKGROUND

The need to collect data on land use and management is recognised by all major ecosystem monitoring activities. The major elements are location (which links to other

data on topography, climate etc); land cover (in terms of vegetation, allowing linkage to remotely sensed and other data); manipulations by land managers and scientists (to enable the interpretation of ecological change to external drivers). The NEON data products[5] include a set of high level land use products that record from external databases site position, topography, soil type, land cover, agricultural management, climate, built infrastructure etc. The UK Environmental Change Network comprises smaller sites, and includes protocols for collating data on land use, vegetation and soils *in situ*[6]. These protocols are very flexible, reflecting the different circumstances of the individual sites.

This protocol ensures that essential contextual data are routinely collected for every spatial unit that is being monitored within the ExpeER infrastructure. It gives a description of the site, and also to provide contextual data on land operations to help interpret (and even help model) ecosystem changes. The protocol ensures that essential data are collected to a basic level. Local protocols may exceed these standards (e.g. by collecting Level 3 EUNIS data). It is of most value for those sites that are managed by farmers and foresters, as it ensures that data are collected in their activities in a timely and consistent way. Data are required for each spatial unit on the site that has consistent management (this may be a field, an area of forest managed as a unit, or a plot within an experiment, or chamber within an ECOTRON). The data are typically collected as part of forest, agricultural or experimental record keeping.

The protocol requires that each spatial unit with consistent management that is being observed within the ExpeER infrastructure is identified; this could be field, a homogenous or even-aged forest patch, an experimental plot, an ECOTRON chamber. These units should change only rarely. Data should be collected for each unit.

METHODOLOGY

Measurables

This protocol involves the precise location and description of land units within ExpeER. These land units are normally all of those from which additional data are being collected. The first two sets of data characterise the site: they need to be checked once a year, but will range change. The rest of the data ensure that unique records are kept on all human interventions on the site by farmers and land managers, and should be accurate to the nearest day; the units will vary and often may not be available directly. Interventions by scientists must be accessible using this protocol, either because the data are collected as part of this protocol, or because a link is created to data held elsewhere (e.g. in an experimental protocol for an ECOTRON study).

- *Site description.* Metadata are collected for integration with the ExpeER metadatabase, but at the level of the individual parcel of land or experimental plot. These include location, ExpeER site identification, and a local code to give a unique iden-

5 http://www.neoninc.org/sites/default/files/NEON%20high%20level%20data%20products%20catalog%20Spring%202010.pdf

6 http://data.ecn.ac.uk/Data_discovery/search.asp#Keyword_Search

tifier to the field, experiment, experimental plot, ECOTRON facility, ECOTRON dome, mesocosm etc. The units need to be sufficiently accurate to discriminate between the unique land parcels, plots or experimental unit.

- *Land cover.* The EUNIS Level 2 vegetation type is recorded for each land unit. This is a simple descriptor of vegetation cover taken on the ground, it is therefore more precise in both space and time than the CORINE land cover map. This code is high level, requires little training, and only needs to be recorded annually.
- *Biotic inputs and removals.* Information about deliberate introductions and removals of organisms or plant parts from the individual land parcel, including sowing and harvesting of crops or trees, removal of weeds or forestry thinning, introduction or removal of grazing animals, additional of compost.
- *Abiotic inputs and removals.* Here data are collected on inputs of fertilisers, pesticides, water for irrigation. Removals of abiotic materials from an ExpeER site are likely to be much rarer.
- *Land management*. This information is intended to provide a formal record of experimental and land management operations. These include disturbance (ploughing, cultivation, cutting of grass without removing it) and manipulation (climate manipulation, CO_2 elevation, etc.).

Frequency

Data on site location should change only rarely, for example by redefining the land parcel size, and data on land cover may show gradual change or sudden change associated with major changes in land use. These data need to be collected once a year, on a date that can be set locally. Data on inputs, removals and land management should be recorded to the day, with date recorded. Such data should be recorded within 7 days of the event.

Site selection

In this protocol, sites are arranged hierarchically, from ExpeER infrastructure down to individual plots or replicates within a controlled environment experiment. This protocol is to be applied to each spatial unit with consistent management that is being observed within the ExpeER infrastructure.

Site description

Data on location will be held at the site level. This protocol ensures that location data are collected at the smallest scale used for observation within ExpeER, and can be used to cross reference with databases on topography, climate, CORINE land cover etc. This information needs to be updated every time a new experiment or observation is initiated within ExpeER, and checked annually and updated if required.

ExpeER Site ID

This unique site code is fixed by ExpeER. It is a constant.

Land parcel/facility ID

This is a locally-fixed code for the land parcel within the site being considered. The boundaries of the land parcel must be fixed over a period of years (e.g. field, forest). Natural boundaries may be appropriate in semi-natural areas, but they must be visible from the ground and by fixed. It may apply to a site geodatabase. The code is established locally, e.g. for the site geodatabase. In a controlled environment, it may apply to a particular set of ECOTRON chambers that comprise an experiment etc. It is a constant.

Description of the land parcel/facility

This explains the nature of the land parcel or spatial unit and why it is used in ExpeER. If the site contains an experiment, the purpose of the experiment should be given. Likewise if it is a bank of ECOTRONS, they must be described. This a text field, that needs checking annually.

Single treatment/field experiment/controlled environment

It is important to establish the nature of the land parcel. If the parcel contains a single treatment, then the following data are recorded for the whole parcel. If the area contains a field experiment, then the following data are recorded for each plot (e.g. a line for every replicate of every treatment). If the data is a bank of ECOTRONS, each line corresponds with each chamber. There is no need to complete a separate line for every plant container within each chamber.

Grid reference

This locates a particular fixed point within the land parcel or facility, ideally coinciding with a major sampling point (e.g. flux tower), not close to one edge. The grid reference is recorded using the INSPIRE grid reference system. It is a constant.

Area

This relates to the area of the parcel, not the whole site. It should be recorded using GIS to the nearest 1 m^2, though in practice the accuracy will be rather less. It is a constant. Areas of plots and chambers must be provided. This is a constant.

Designations

This identifies whether the land parcel has a national (e.g. English Site of Special Scientific Interest) or internal (e.g. Natura 2000) designation, what the designation is, the reason or feature underlying the designation, and date of designation. It is a constant for any parcel and any year. It needs to be checked annually using GIS and international databases or whole site records, in case the parcel becomes newly designated. Some sites may have land parcels with different designations. This will not apply to small plot or controlled environment experiments.

Soil and vegetation in situ, or enclosed/imported

For field-scale sites, the soil and vegetation is always likely to be *in situ*, but for controlled studies the soil are sometimes imported or enclosed, e.g. in rhizotrons, and the plants often sown from other sources. Give a description. For field sites this is a constant; for experimental facilities it may change between experiments. The date of any change must be recorded.

Land Cover

EUNIS habitat classification

The vegetation cover on the unit of land is classified using the EUNIS Level 2 classification every year by the site science team. See for details[7], which includes a simple key to support the identification of habitat classes.

Biotic inputs and removals

These data should be held by the person managing the site, which could be a scientist, forester or farmer. It is essential that the data are recorded from the site manager each year at least, to ensure data quality. The level of detail available will vary from situation to situation. Timings should be given to the nearest day.

Biotic inputs

All species introductions should be recorded. These include crop plants and animals, plants sown into experimental plots, and biocontrol agents. Data should include date, species, variety (if relevant), how introduced, density/numbers, etc.

Thinning and removal

All removal of biotic material not for harvest must be recorded. This includes weeding, thinning of forests, trapping of animals. This includes mechanical weeding, and burning. Date and nature of removal must be recorded. Ideally, the biomass and species of organisms removed from the site should be noted, e.g. thinning or hand-weeding.

Harvesting

All harvesting must be recorded, including removal of livestock from a field, harvesting plant material from an experiment, as well as commercial scale operations such as hay cutting, forest felling. Date and nature of harvest must be recorded, along with biomass removed and, ideally, species composition.

---

7 http://eunis.eea.europa.eu/upload/EUNIS_2004_report.pdf

Abiotic inputs and removals

These data should be held by the person managing the site, which could be a scientist, forester or farmer. It is essential that the data are recorded from the site manager each year at least, to ensure data quality. The level of detail available will vary from situation to situation. Timings should be given to the nearest day. The major two categories are given below, there may be others.

Nutrient inputs

All nutrient inputs must be recorded, giving amounts of N, P, K and S for all inputs, including organic manures and slurries. Micronutrients should also be recorded if data are available. Give method of application (e.g. spraying, soil injection, liquid plant feed).

Pesticide use

All pesticide inputs must be recorded, including chemical pesticides, slug pellets, etc. Data should include date, active ingredient and adjuvants, method of application.

Land management

These data should be held by the person managing the site, which could be a scientist, forester or farmer. It is essential that the data are recorded from the site manager each year at least, to ensure data quality. The level of detail available will vary from situation to situation. Timings should be given to the nearest day. The major two categories are given below, there may be others.

Soil operations

All soil operations (tillage, drainage, etc.) must be recorded.

Experimental manipulations

These include climate manipulations, additions of gases, elevated levels of UV, etc.

Data capture

The vast majority of data needs to be captured from other sources. These can include electronic cross-reference to an experimental protocol and records or site description, and transcription of farmer and forestry records. The only data that will be collected *de novo* will be the EUNIS classification, which can be entered in the field as a single record for each unique spatial unit. This should be recorded in the field, and entered into the site database asap afterwards.

Date and time of measurement

Data should be updated at least annually, and should be accurate to the nearest day.

Quality assurance

The recoding of EUNIS should be by an experienced vegetation surveyor or should follow training. The protocol does not have other training requirements.

Once a year, when the data are updated, a scientist not involved in completing the protocol should check the data and sign them off as complete. This will involve cross-checking against any source data that are used.

SUMMARY INSTRUCTIONS

1. This protocol must be completed at least once a year. As the protocol is not field based, timing is not critical. Some data will not change between years, but should always be checked.
2. Identify the major spatial units of the time with homogenous management – e.g. fields, even-aged forest stands, experimental plots, chambers in an ecotron. These land parcels/facilities should rarely change from year to year, and have unique codes to identify them.
3. Data about each land parcel or facility must be recorded. These data should change only rarely between years. The data are:
   a) description of the spatial unit: a text field, to describe its nature and purpose;
   b) is the land parcel or facility a whole or part single field, a field experiment or controlled environment facility?;
   c) location (grid reference) using GPS or existing database;
   d) area, using GIS;
   e) is the land parcel or facility designated for biodiversity or landscape reasons? Use online database and GIS, if data not already held at site level;
   f) is the soil and vegetation on the land parcel/facility the pre-existing one, or has it been imported? Has the vegetation cover been sown or imported? Describe.
4. Use the attached key to give a unique EUNIS level 2 classification of plant cover for each land parcel/facility. This is sufficiently broad that precise timing of recording does not matter.
5. Data on inputs of pesticides and fertilisers, other abiotic inputs and removals, should be recorded to the nearest day, and to the available level of accuracy about quantity. Such data may come from the farmer or forester. More precise data will be available for formal experiments, in which case it is probably preferable to refer to the database of the experiment itself using appropriate links.
6. Data on biotic inputs, thinning and removal, and harvesting, should be recorded to the nearest day, and to the available level of accuracy about quantity. Such data may

come from the farmer or forester. More precise data will be available for formal experiments, in which case it is probably preferable to refer to the database of the experiment itself using appropriate links.

7. Data on soil operations, experimental manipulations and other forms of land disturbance and manipulation not covered above, should be recorded to the nearest day, and to the available level of accuracy about quantity. Such data may come from the farmer or forester. More precise data will be available for formal experiments, in which case it is probably preferable to refer to the database of the experiment itself using appropriate links.
8. Once complete, the data should be signed off by an independent scientist, and maintained and archived according to local data management practices.

LEAF AREA INDEX

Giorgio Matteucci and Miklós Kertész

ECOLOGICAL INTEGRITY INDICATOR

Energy input.

MEASURABLE

Leaf Area Index.

PROTOCOL SUMMARY

This protocol is related to the measurement of Leaf Area Index (LAI), which represents a basic structural and functional variable of terrestrial ecosystems. Leaf Area Index is defined as the total one-sided foliage area per unit ground surface area. LAI has relevance for radiation interception by the ecosystem, and is usually closely connected to its Net Primary Production. LAI can be assessed by direct (e.g. collection of falling leaf litter; harvesting of grass, herb or crop; allometry for assessing foliage biomass) or indirect methods (all based on the interception of incoming radiation by the canopy). The latter are usually more suitable for ecosystem of a certain height (e.g. forests, shrublands) or a certain spatial arrangements (e.g. croplands, tree orchards). For those systems where it is applicable/feasible, it is advisable to assess LAI at least once with both methods (direct, indirect). Leaf Area Index can be used in connection with remote sensing derived indexes (e.g. NDVI, Normalised Difference Vegetation Index), and has hence a potential for upscaling and continuous monitoring of ecosystem features.

KEYWORDS

Leaf area index direct measurements, leaf area index indirect measurements, leaf litter, leaf mass per area, specific leaf area.

SCIENCE BACKGROUND

Leaves are the active interface of energy, carbon and water exchanges between vegetation canopies and the atmosphere. The leaf component of a canopy may be quantified

by its structural attribute Leaf Area Index (LAI, one sided projected leaf area per unit of ground area). This important parameter regulates a number of ecophysiological processes, such as evapotranspiration and photosynthesis, and is related to stand productivity. Furthermore, LAI is a key variable in various stand- and regional-scale models and it is a variable with very close connection to remote sensing (Cutini et al. 1998). Hence LAI is a very important structural and functional index for ecosystem characterisation in ecological research and monitoring.

LAI can be measured directly by collecting leaves or tree allometric relationships in forests (see protocol on above-ground biomass) or harvesting small parcels of vegetation in grassland and cropland. As direct measurements of LAI are usually difficult and time-consuming, indirect procedures based on the measure of light transmission through plant canopies have been developed.

Knowledge of the key parameters of carbon cycle is essential in understanding of the function of ecosystems. However, the measurement of those parameters, especially in terrestrial ecosystems, is very costly and labour intensive, thus, a combination of different measurement, including direct measurements, measurements on proxy variables, and calibrations are applied for estimating of the key variables.

In case of temperate steppe biome, under certain conditions (unimodal yearly growth pattern of living plant biomass, regular removal of plant biomass after the yearly peak), maximum yearly plant biomass is an appropriate estimator of annual net primary production (ANPP). In a given terrestrial biome, the living plant biomass is strongly correlated with leaf-area index (LAI). LAI is extensively estimated by means remote sensing. The ANPP can be estimated using the yearly pattern of LAI. Thus, direct measurements of yearly maximum living plant biomass and LAI give opportunity to apply a broad spectrum of known correlations, as well as crop production simulation results, for estimating ANPP.

METHODOLOGY

Leaf area index in forests.

Measurables

Leaf Area Index

Leaf Area Index (LAI) is defined as the total one-sided foliage area per unit ground surface area and represent a basic structural and functional variable of terrestrial ecosystems. LAI can be assessed by direct (e.g. collection of falling leaf litter; harvesting of grass, herb or crop; allometry for assessing foliage biomass) or indirect methods (all based on the interception of incoming radiation by the canopy). The latter are usually more suitable for ecosystem of a certain height (e.g. forests, shrublands) or a certain spatial arrangement (e.g. croplands, tree orchard). For those systems, where it is applicable/feasible to increase reliability of LAI measurements, it is advisable to assess LAI at least once with both methods (direct, indirect).

Methods

Direct methods

In deciduous forests, Leaf Area Index can be assessed directly by collecting falling leaves by a number of litter traps (funnels), weighing them and assessing the ratio leaf area to weight on a subsample of collected leaves. In evergreen forests, falling needles do not equal standing leaf area but are, in the medium-long term and on the average, equal to annual foliage production. Hence, in evergreen forests, direct measurement of LAI can be made using allometric relationships developed to assess tree components biomass (see protocol on biomass).

In grassland and croplands, direct measurements of LAI can be made by harvesting small parcels of vegetation, weighing the harvested biomass and measuring the ratio leaf area to weight on a subsample of collected material.

LAI in forest[8]

LAI for a plot for each year is computed from total leaf litter dry biomass of that species in that year (Jan–Jan) per m^2 multiplied by a ratio to convert dry weight to leaf area.

Sampling and traps design

Leaf litter is collected by litter traps (collectors). It is recommended to sample litterfall from at least 10 collectors per plot under uniform forest canopy, and up to 20 or 30 collectors under mixed species or in larger plots with uneven topography. Leaves from deciduous trees are more susceptible to turbulent air movement than conifer needles. This effect may be mitigated either by increasing the number of litterfall traps (e.g. 10 traps for coniferous species and 20 traps for deciduous species) or by increasing the collecting area of each trap (especially for species with large leaves).

Litter traps should be distributed all over the plot area. The traps are fixed and may be placed randomly or systematically, e.g. at regular intervals and in sufficient number to represent the whole plot and not only the dominant tree species. Figure 1 gives examples of two litterfall trap designs.

It is recommended that the litterfall traps are not fixed too close to the ground, to ensure adequate water drainage. The opening area of the collectors must be horizontal.

Canopy leaves (and other litterfall) inputs are collected in nets or litter bags. These nets/bags are attached to a frame of durable material, with a known area of minimum 0.18 m^2, preferably 0.25 m^2, but larger area can ensure more reliable results (e.g. 0.5 m^2). The sampling area must be sufficiently large to be able to determine litterfall amount (10 to 20 traps per plot). For tree species with very large individual leaves, the collecting area of individual traps must be increased (i.e. up to 0.5 m^2).

[8] Protocol based ICP Forests procedures as modified within LIFE+ FutMon) (ICP Forests manual are available at http://icp-forests.net/page/icp-forests-manual

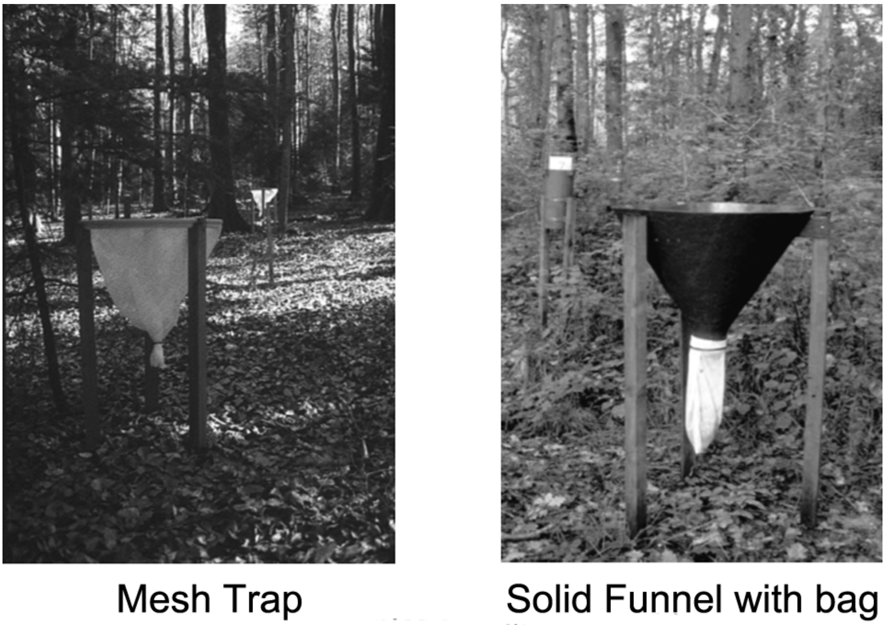

Figure 1. Examples of litter traps design

It is recommended that the litter bags or collecting funnels are at least 0.5 m deep to prevent litter from blowing out of the traps. Deposition of litter into these traps due to lateral movements by wind is assumed to be minimal. The material of the mesh must not interact with the litterfall sample. The mesh size of the bags must be large enough to allow for easy drainage of water. It is recommended to adapt mesh size to the dimension of smallest elements, i.e. for needles from coniferous species up to 0.5 mm (but if there is interest in other litter input such as finest "frass" material (caterpillar droppings), then the texture needs to be much smaller). During the winter season in areas of heavy snowfall, traps may lowered on to the ground to avoid breakage of the collector structures.

Sampling frequency

It is recommended that litterfall be collected at least monthly and even bi-weekly in periods of heavy fall, which may be co-incident with heavy rainfall. This is to avoid pre-collection decomposition in the bags. The samples may be pooled to periodic or annual totals, once the monthly variations in amount (and quality) have been investigated. In regions with snow in the winter or which are very remote, it may be impossible to empty the traps at regular intervals. Litterfall may then be collected once before the winter period and once after snowmelt, as frost will limit both drainage and litter decomposition. Total values for this period should then be subdivided proportionally to the months passed since the first collection.

Converting dry weight to leaf area

The ratio leaf area/dry mass is named Specific Leaf Area (SLA) and its alternative expression is as LMA (leaf mass per area):

$$SLA = area/wt\ (m^2\ g^{-1})$$

$$LMA = wt/area\ (g\ m^{-2})$$

SLA can be recorded on both fresh weight and dry weight bases, but the latter gives better standardisation between sites. It has to be determined for each main canopy species on a random subsample of litter leaves (at least 100 leaves from different traps). Preferentially, five replicates from one year leaf litter total should be analysed to obtain a measure of the variability of the material from the site accruing through the year. SLA can be measured leaf by leaf or in bulk as an annual value smoothing out the variations of the individuals. After measurement leaves should be oven dried for 48 hours at max 80°C, and then allowed to cool in a dry place before being weighed.

Canopy Leaf Area (LAI m^2/m^2) can be calculated from these weight/area values (SLA) combined with the litterfall leaf biomass accrued throughout the year.

If litterfall leaves are dry, either through storage or oven treatment, they will be more fragile than green leaves. If they are taken wet, they are likely to be more dirty than freshly fallen leaves, and may need to be cleaned and flattened before leaf area measurement. If canopy representative measures of SLA are needed, leaves as complete as possible need to be measured, or at least have a balance of mid rib and petiole. For dried litter leaves either folded or curled, a soaking technique may be required to ensure sufficient flexibility for measurement. This is possible for most broadleaves. Occasionally, for very thin leaves (e.g. *Fraxinus excelsior*), area losses may also occur. Test on each species collected should be conducted to establish a standard treatment with a known effect. In the case of dehisced *Fagus sylvatica* leaves, which dry folded into a concertina, a brief soaking in hot water (60-70°C) has been found to flatten leaves sufficiently for measurement, but weight losses of 5% have been recorded after longer overnight soaking. However, for *Quercus robur* and *Quercus petraea* leaves weight loss is minimal over the same time period. For thinner leaves such as *Corylus avellana*, or *Fraxinus* spp., a soak of an hour or so will be sufficient, as weight losses of up to 15% weight have been recorded after long soaking.

Any weight loss due to a soaking procedure should be incorporated into the SLA calculation as a correction factor before LAI is calculated from the litterfall weights.

For short conifer needles, which have dried (e.g. spruce), area measurement is often obtainable after cleaning, as they do not change area. However, finer needles (e.g. *Larix*) are difficult to prepare, and twist on drying. These would need a short soak and would be best measured on a hand swipe machine, where they can be laid flat. Longer needles (e.g. some *Pinus* species) also twist on drying, and are very difficult to soak out, as they then break up. Area/width/length measurements are best made from these, if they can be kept damp from abscission.

All samples should then be dried at max 80°C for 48 hours before weighing for calculation of SLA and LAI.

Leaf area measurement

Leaf area can be measured with suitable commercial equipment. As an example, samples can then be passed through the rollers of, for example, the Cl-203 Laser area or the Delta –T Leaf Area machines. Nowadays it is also possible to use image-scanning techniques. Software are available also on the web to evaluate the area of samples against a background of known area (e.g. leaves over a A4 paper or over a A4 scanner).

Indirect methods[9]

Beneath the direct determination of LAI from the litterfall samples (direct method) it is possible to make respective assessments from various radiation measurement methods (indirect methods). Indirect LAI measurement with optical/light absorption techniques are usually more suitable for ecosystem of a certain height (e.g. forests, shrublands) or a certain spatial arrangement (e.g. croplands, tree orchard). In addition to LAI, some of the indirect methods produce additional parameters, such as gap fraction, biomass indices and photo documentation of the site.

Sampling design

In order to get an estimate of the variation of the leaf area, and so of the crown structure and light conditions under crown, a systematic sampling design can be recommended. The sampling design will depend on the size of the plot and on canopy height (see Quality Assurance). In ICP Forests, where the minimum plot size is 0.25 ha, a grid net of at least 10 × 10 m resolution is proposed.

If a measurement point is lying within a distance less than 2 m from an obstacle (e.g. tree or bolder) the measuring point is moved so that it is at least 2 m away from all obstacles. Each point must be marked permanently in order to allow for repetition in following seasons/years.

A measurement height of 1.5 m is defined in order to avoid disturbances by lower shrubs or installed litterfall, or other samplers which could disturb the radiation measurements. It could be wise to perform measurements in the proximity (photograph) or above (optical devices such as LiCor LAI-2000) the traps for litterfall collection. The location of each measurement point should be documented.

Frequency of sampling

Time frame for LAI determination (field survey):

All measurements are made in the following time frame:

- summer measurements: during full crown development, depending on tree species (ICP Forests: 16th July to 15th August);

9 Based on experimental protocol developed within LIFE+ FutMon, http://www.futmon.org/

- winter measurements on deciduous tree species: during time without leaves;
- it is possible also to evaluate the seasonal course of LAI development by measuring LAI in different time of the year.

Using Canopy analyzers

Instrument Name: Li-Cor LAI-2000 Plant Canopy Analyzer (Li-Cor Inc., Nebraska, United States).

Pre-defined settings of the equipment during field work:
- preferably uniform overcast sky without any direct radiation contribution; alternatively on sunny days around sunrise and sunset (ideally);
- 30 seconds logging on clearing or above canopy measurement; use closest-in-time measurement for linkage with below canopy measurements;
- clumping factors, shoot/needle index need to be retrieved from hemispherical photographs, TRAC measurements, direct methods, and/or from literature;
- follow strictly the advices given in the manual:

  ftp://ftp.licor.com/perm/env/LAI-2000/Manual/LAI-2000_Manual.pdf

- date and time as well as the weather conditions have to be specified as precise as possible.

If view caps are used, it should be recorded which one and towards what direction the measurement was performed.

Data management and Parameter Outcome

Clumping factors, shoot/needle index need to be retrieved from hemispherical photographs, TRAC measurements, direct methods, and/or from literature. FV2000 Data File Viewer (new Windows program) is recommended and should be preferably used instead of older versions under DOS.
- A is Above
  In general, the horizontal canopy model should be used (default), unless the stand is too small or has a very heterogeneous upper surface. The instrument optic measures trasmittance using five "rings", viewing different portion of the sky/canopy.
  In deciduous forest canopies, comparison of LiCor LAI 2000 LAI measurements have shown that a recalculation of data based on four rings, with the omission of the reading of the 5th lower ring provides much better match with direct LAI (Cutini et al. 1998).
  In case of recomputation of data collected in the field for the above or other purposes, use following set-up (and follow manual):
  Recompute Transmittance:
  – use the Closest in time above (A) record,
  – transmittance data larger than 1 should be "forced" to 1,
  – omit reading of the lower ring.

TRAC (Chen)

Another indirect method is the Tracing Radiation and Architecture of Canopies (TRAC). A handheld instrument is used in the forest, the latest software version at moment of Protocol preparation is TRACWin (2.3.4, 11.2007)[10]. Predefined conditions:

- clear sky,
- optimal solar zenith angle: 30-60° (best – close to 57°),
- position of transect perpendicular to sunbeams.

For the calculations and settings of the instrument, following information must be known and documented:

- Mean element width (mm),
- Needle-to-shoot ratio,
- Woody to total area ratio,
- Spacing between markers of the transect (m),
- Light above canopy,
- Zenith angle,
- Coordinates (geographical) Latitude Longitude,
- Time longitude reference,
- Computer clock.

In addition to gap fraction (i.e. the share of caps in a crown at a given solar zenith angle), gap size distribution is determined (i.e. the physical dimension of a gap in the crown). This allows for the direct determination of the clumping factor and the integration of it during LAI determination, which is underestimated if the clumping effect is neglected.

Data output:

- photosynthetic flux density (PPFD) along a transect as way to obtain the mean value of the transmitted light through the canopy; used to quantify the fraction of photosynthetically active radiation (FPAR) absorbed by the canopy (Chen, 1996);
- gap size distribution (Chen and Cihlar, 1996).

Main Parameters outcome (summary):

- Mean Gap fraction,
- PAIe (Plant Area Index),
- Mean PAIe,
- Mean LAI,
- OMEGA.

Hemispherical images analysis

For all photos it is essential that the direction of the top of the photograph is directly to north measured with magnetic compass.

- Ideal conditions: uniform overcast sky Alternatives: before sunrise and at/after sunset.
- Use aperture of 5.0 to 5.5.

[10] http://faculty.geog.utoronto.ca/Chen/Chen's%20homepage/res_trac.htm

- Start with measurements/photography with the darkest measurement point.
- Use -2 underexposure to the automatically exposure under canopy; alternatively use +2 overexposure to the automatically exposure over canopy or at open area can be chosen and fixed for photography under canopy; this presumes constant weather and light conditions during the field observation; the use of a Notebook during field observation for direct control of photo quality is recommended. Also a set of photographs from -2 under exposure to +2 overexposure in the stand could be taken, in order to select afterwards the best image from each measurement point for further operating.
- Image format standard: .jpg (high image quality settings).
- Filter usage: in their standard setting, many digital cameras apply a software filter to sharpen the picture. This filter should be turned off in order to avoid small errors and increase reliability of photos.
- Diffusion model must be documented with each photo evaluation settings.

General guidelines for data processing:
- Use automatic mode to determine threshold values; underestimation is accepted here, comparability of outcome of higher importance?;
- Use colour mode instead of black/white if any available.

For photo analysis three methods are recommended:
- **WinScanopy, HemiView, and Gap Light Analyzer.** Those methods for interpretation of hemispherical photographs are briefly described below. Any method used has to be documented, in order to allow for a linkage to each resulting LAI value and photo document. If settings have to be changed from one photo to the other or, e.g. from one site to the other, they have to be documented in addition.
- **WinScanopy**[11]. The WinScanopy system concludes all instruments, which are needed for the determination of LAI starting with the camera, lens, and specific tripod up to the evaluation programme. All recommendations made in the manual and in this field protocol should be followed during field work.
  The recommended version of the Software is WinScanopy pro 2003 d pro version, which makes possible to evaluate photos in batch mode. Some screenshots of the programme showing settings for data processing:
- **HemiView**[12]. Camera systems which are recommended: Nikon, Canon, Minolta used by respective expert in project: Nikon Coolpix 4500 with FC-E8 (Zhang et al. 2005 protocol):
  - Common Lenses: predefined – FC-E8/Coolpix 4500 (990 series), FC-E9/Coolpix 8400 series, Sigma/Canon SLR; possible to add any hemi-lens, based on few parameters.

Useful additional devices:
  - Self Leveling Mount System: SLM6-UM-1, Delta-T Devices Ltd.
  - Tripod: Manfrotto 681B.

[11] http://www.regentinstruments.com/products/Scanopy/Scanopy.html
[12] link to manual and webpage http://www.delta-t.co.uk/groups.html?group2005092332185

– Software: Hemiview 2.1
  A description of the system can be downloaded from:

  http://www.delta-t.co.uk/groups.html?group2005092332185

  Manual:

  ftp://ftp.dynamax.com/Manuals/HemiView_Manual.pdf

– Aperature: preferred 5.3 or similar

  Data processing:
  Software: Hemiview 2.1 (Delta-T Devices Ltd.):

  http://www.delta-t.co.uk/groups.html?group2005092332185

  Manual:

  ftp://ftp.dynamax.com/Manuals/HemiView_Manual.pdf

  Output:
  - LAI in Skymap Sectors (LAI)
  - LAI by Angle Class (LAD).

Alternative photo devices and Gap Light Analyser (Freeware)

In principle many cameras and lenses may be used in order to get hemispherical photographs. These may be evaluated using specific software as the proposed systems above do or using available Freeware. In this chapter an additional system is proposed, in order to underline that alternative devices may be used, and in order to give an example for a respective documentation of the devices and methods, which are applied in the field. Overcast sky conditions are recommended to avoid reflections on the lens and also to avoid blooming effect, i.e. when there is uniform cloudiness or in the hour before sunrise or after sunset, when no direct solar radiation is present.

Example:
Nikon CoolPix E8700[13] *with the Nikon Fisheye Converter FC-E9*[14]

- Basic camera settings.
- Aperture: 5,0.
- *Exposure:* -2 steps or follow up from -2 steps to automatic exposure under canopy.
- In their standard setting, many digital cameras apply a software filter to sharpen the picture. This filter should be turned off, because it can also introduce small errors.
- “Fish-eye setting” of the used camera means the zoom is fixed at widest angle and focus is fixed at infinity.

13 http://www.nikonusa.com/pdf/manuals/coolpix/CP8700_en.pdf
14 http://www.nikonusa.com/pdf/manuals/tnirp/Coolpix_Accessories/Coolpix_Converter_lenses/FCE9.pdf

Software:

- Gap Light Analyzer 2.0 (GLA)[15] (freely available).
- Software Manual: included in the installation (GLAV2UsersManual.pdf).

Outcome:

- Initial and final point of evaluation have to be documented with each LAI value or photo interpretation, respectively.
- Total Openess, Gap Fraction, LAI 2000G.

Direct leaf-area index measurement in grasslands and crops

The direct measurement based on harvesting of the aboveground part of the vegetation (see section **Above-ground biomass in grasslands and forests in chapter Above-ground plant biomass**). Thus, aboveground biomass measurement is always part of direct LAI measurement in case of grasslands and crops (Milner and Elfyn Hughes, 1968).

The metadata should consist of geographical coordinates, elevation, exposition, habitat type, and sampling scheme, including number, size, and arrangement of sampling units. The specific measurables are as follows:

- *Yearly maximum of aboveground biomass of vascular plant.* Measurements of the aboveground biomass of mosses and lichens are optional. In case of woody plants (shrubs, dwarf shrubs, vines), the biomass of the offshoots of the last growing season should be measured. Yearly maximum aboveground biomass data for each vascular species separately are optional.
- *Specific leaf area (SLA).* SLA should be separately measured for either each species or for each seemingly different leaf types. SLA data for each vascular species separately are optional.
- *Yearly maximum of leaf-area index (LAI).* LAI should be separately measured for either each species or for each seemingly different leaf types. Yearly maximum LAI data for each vascular species separately are optional.

Site selection

The criteria of site selection are as follows:

- *Vegetation.* The vegetation should be dominated by soft-stemmed vascular plants.
- *Long-term security.* The treatments of the sites should be as permanent and predictable at long term at possible.
- *Homogeneity.* As the sampling units are relatively small (0.5×0.5 to 1×1 m^2), the biomass and LAI of the studied plots should be homogeneous enough to be reliably sampled by a few sampling units.

[15] http://www.ecostudies.org/gla/

- *Representativity.* It is desirable that the sites represent areas which are characteristic to the region and consist of patches large enough to provide reliable remotely sensed data.

Method

- *Sampling units and design*. Square shaped sampling units are used, from 0.5×0.5 m^2 to 1 by 1 m^2. The number and arrangement of sampling units have to be reliable estimation of biomass and LAI of the plot. Depending on the heterogeneity of the grassland, application of one to five sampling units is suggested per plots.
- *Timing*. In grassland mown once a year, sampling should be made right before mowing. Otherwise, or in case of multiple mowing, the sampling should be made once a year, at the time of the maximum LAI.
- *Clipping*. As much as possible, clipping should be made right at the level of the ground. The old bunches of grasses may form small mounds of dead plant material and soil; that part should be left on the ground. In case of wetlands, the ground can be a soft net of mosses and roots. There the ground could be determined by the lowest level of seemingly green plant material. The woody parts of plants which are seemingly older than one year, may be left in the sampling units.
- *Drying*. All plant material should be dried at 60°C until constant weight. If this were impossible to achieve, dry at room temperature in well ventilated dry room until constant weight is satisfying.
- *Separation into fractions*. If it is possible, separation must be done while the plant material is still wet. It is often impossible, thus the dry plant material is separated. In this case, the status of plant material should be extrapolated back to the time of clipping. Three fractions should be form: (1) plant material which was dead in the time of clipping, (2) non-photosynthesising living material, and (3) photosynthesising material. The woody parts of the pants which were seemingly grown in previous seasons should be counted as dead material. If it could not be decided whether a part of the material were photosynthesising or not at the time of clipping, it better to count as photosynthesising part. Theoretically, only leafs are counted as photosynthesising parts, the otherwise green stems and inflorescences not. However, in case of certain plants, the stems provide the photosynthesising surfaces (e.g. the stems of *Equisetum* species). In these cases, the stems are also counted as photosynthesising parts. The plant material can also be separated by species or other groups.
- *Biomass*. Biomass is calculated by summing up the dry weight of all living material (fractions 2 and 3), and expressed in g/m^2.
- *Preparation for area measurement*. The photosynthesising material should be separated into groups consisting parts of seemingly similar morphology, and therefore SLA. Then, the groups are weighted, and subsamples of known weights are taken for area measurement.
- *Area measurement*. A few firms offer special leaf area lab meter with conveyor belt, e.g. LI-COR's LI-3100C Area Meter. However, any high resolution scanner can be used for area measurement applying simple image analysis. Preparing the

plant material for scanning, small pieces and varyingly rigid dry plant materials can be effectively slick down by sticking them to adhesive transparent sheets, and then cover them with another transparent sheet, i.e. filming them. High resolution monochrome picture can be produced by scanning with suitably selected threshold of brightness, and then the number of black pixels stands for the total leaf area on the picture. The scanning parameters could be set by trial and error method using narrow (2-3 mm) stripes of papers of varying thickness, up to at least 1 mm.

- *Calculation of SLA and LAI.* The subsamples of known weight and area provide the SLA in m^2/g for the morphology groups; the total area of the groups can be calculated by multiplying their SLA and weight. Sum of the areas of the morphology groups divided by the area of sampling unit provides LAI in m^2/m^2.

Data capture

Data from direct measurements (litterfall; harvesting of grass, herbs, crops; SLA or LMA) can be organised in spreadsheet. It is important to always report measurement date along with the data line. When using a spreadsheet, it is informative to maintain the underlying data for final LAI calculation (e.g. foliage biomass, Specific Leaf Area, etc.) that can be then used and compare with past and future measurements.

Data collected using indirect methods are usually organised according to the instrument data handling and downloading set-up and can then organised in spreadsheets or tables. Software for the calculation of LAI from hemispherical or similar images usually calculates LAI and several accompanying variables (e.g. gap fraction, percentage of intercepted radiation, mean angle of foliage, etc.).

In case of grasslands and croplands, above-ground biomass and specific leaf area of leaf morphology classes (or species) are inherent parts of the collected datasets.

Quality assurance

Leaf Area Index is subject to canopy phenology. For ecosystem characterisation it is important to assess LAI at least at its maximum (normally in the centre of the growing season). As LAI changes with canopy phenology, it is important to always report measurement date along with notes on the phenological status of the ecosystem. For ecosystems with different canopy layers (e.g. trees, shrubs, herbs), it is important to relate LAI values to the correct layer (e.g. whole ecosystem, main canopy, etc.), while in systems characterised by species with different seasonal phenology (e.g grassland with spring and summer species), LAI should be assessed in different seasons.

The number of samples collected (direct methods) or measurements taken (indirect methods) has to be sufficient to assess LAI of the site, at the scale relevant for the analysis (e.g. experimental site or ecosystem characterisation; primary production studies; footprint area of flux measurements; mapping; connection with remote sensing; etc.). As an example, in relatively homogenous forest, 10 to 15 points over a grid covering 0.25 to 0.50 ha can be sufficient to derive forest LAI.

In the case of indirect methods, the coverage of each single measurement can be calculated in advance according to ecosystem structural features (e.g height of trees), and/or the characteristics of camera optics (field and angle of view). Hence, when using these methods, sampling points should be spatially organised in order to have a limited overlap, with a proper coverage of ecosystem variability.

In grasslands (and croplands) the selected sites for clapping should be representative for the above-ground biomass, as well as for the distribution of the weight and area of leaf morphology classes. The area measurement by means of scanner should be calibrated by paper stripes and shapes.

REFERENCES

Chen, J.M. 1996. Optically-based methods for measuring seasonal variation of leaf area index in boreal conifer stands. *Agricultural and Forest Meteorology* 80: 135-163.

Chen, J.M., Cilhar, J. 1996. Retrieving leaf area index of boreal conifer forests using Landsat TM images. *Remote Sensing of Environment* 55: 153-162.

Cutini, A., Matteucci, G., ScarasciaMugnozza, G. 1998. Estimation of leaf area index with the Li-Cor LAI 2000 in deciduous forests. *Forest Ecology and Management* 105: 55-65.

Milner, C., Elfyn Hughes, R. 1968. *Methods of the Measurement of the Primary Production of Grasslands. IBP Handbook No. 6*. Blackwell, Oxford and Edinburgh.

Zhang, Y., Chen, J.M., Miller, J.R. 2005. Determining digital hemispherical photograph exposure for leaf area index estimation. *Agricultural and Forest Meteorology* 133: 166-181.

SUMMARY INSTRUCTIONS

Forests

Habitat: forest, dominated by trees that can reach, at maturity, more than 10 m.

Sampling units/measurement points:

- for indirect measurements: random or gridded points distributed within the target ecosystem and spaced apart so that overlap among measurements performed at adjacent points is minimised. The number of points should allow estimation of LAI of the ecosystem (usually 10 or more).
- for direct measurements: litter traps, to be placed at at least 1 m above the soil. Traps can be distributed randomly or in grid within the target ecosystem. The number of points should allow estimation of LAI of the ecosystem (usually 10 or more).

Measurement method: direct: litter traps or biomass collection; indirect: optical devices, such as LiCor LAI 2000 or digital photograph.

Data: Leaf Area Index ($m_{foliage}^{2}$ m_{soil}^{-2}). In case of direct measurements: leaf biomass in the litter traps (g m^{-2}), specific leaf area (m^2 g^{-1}).

Grasslands and crops

Habitat: grasslands and croplands, dominated by soft-stemmed vascular plants of less than 2 m height.

Sampling units: 0.5 × 0.5 to 1 × 1 m^2 quadrats; the number of units and the spatial arrangements should provide reliable estimation of average above-ground plant biomass, specific leaf area, and LAI.

Measurement method: clipping, drying and weighing, separation leafs into leaf morphology classes (or species), leaf area measurement by scanning.

Data: dry above-ground plant biomass, g m^{-2}, biomass (g m^{-2}), specific leaf area (m^2 g^{-1}) and LAI ($m_{foliage}^{2}$ m_{soil}^{-2}) of leaf morphology classes, total LAI ($m_{foliage}^{2}$ m_{soil}^{-2}).

SOIL MACROFAUNAL DIVERSITY

Amélie Joseph, Elli Groner and Cristina Menta

ECOLOGICAL INTEGRITY INDICATOR

Biotic diversity.

MEASURABLES

Soil biodiversity, Index of soil biological quality.

PROTOCOL SUMMARY

This protocol is intended for the assessment of the soil macrofauna present in a studied site through a simple index, which doesn't involve species identification skills. A high index represents a high diversity of fauna adapted to life in the soil and corresponds to a good biological quality of the soil.

Soil samples are collected *in situ*, and the soil fauna are extracted by a dynamic extraction method (Berlese Tüllgren funnel). The fauna is identified at the level of Recognizable Taxonomic Unit (RTU), which means order or class depending on the specialization of the order. Each RTU found in the sample receives a score from 1 to 20 according to its adaptation to soil environment, following a score grid. The final index sums up these scores. Some orders are consistent in their performance and all the order gets the same score and so identification or RTU is to the level of order. Some orders are heterogeneous in their performance and there is a need to identify to family, which is the RTU.

The data include:

- *Location, area, soil cover* These are the site metadata, plus data for every spatial unit inside the site if necessary.
- *Soil fauna diversity.* For each type of soil fauna, information about presence/absence and score of adaptation to edaphic life, according to the scoring grid supplied.
- *Soil quality index.* Aggregation of the score of each fauna type in a single index.

KEYWORDS

Arthropods, invertebrates, soil biodiversity, soil macrofauna, soil mesofauna, soil quality.

SCIENCE BACKGROUND

There is a need for an index that describes the soil quality, based on the community composition. Species richness, abundance and single species measures may not represent the soil quality. Several indices have been developed.

The simplest approach is using a single species as an indicator (e.g. Hogervorst et al. 1993). However, this method not only requires finding such a species, it is also not likely that a single species could be an indicator of many different drivers and pressures. Also for such a large scale project, it is less likely that a single species would be suitable. Diversity indices such as species richness, Simpson or Shannon-Wiener react in a very unspecific way, because so many parameters can affect them (Van Straalen, 1998). Multivariate analyses have been shown to be good methods of showing different effects, but are descriptive and lack the bio-indication quality. The index that is based on the ratio of termites to earthworms, has the potential to be a suitable one, but lacks validation. The maturity index (Bongers, 1990; Ruf, 1997) is based on a soil quality classification using biological criteria. It is based on known preferences for each taxon. A high level of maturity indicates a low level of disturbance. The "acidity index" (Van Straalen, 1998) shares the same qualities and problems as the maturity index, in synthesising different characteristics into one index, and is designed to specifically test the effect of acidity.

Soil quality monitoring is often inaccessible to land managers because the measurement systems are too complex, too expensive or both (Herrick, 2000), despite its utility as an indicator of environmentally friendly use of natural resources. The application of biological indicators is often limited by the difficulties in classifying the soil fauna. Therefore, we use a simplified eco-morphological index that does not require the classification of organisms to species level: which allows a wide application without specific technical skills.

Here, we propose an efficient and low-cost biological index of soil quality. The QBS-ar index (Qualità Biologica del Suolo) is based on the following concept: the higher is the soil quality, the higher is the number of microarthropod groups morphologically well adapted to this soil habitat. This protocol, through the study of the soil macrofauna, provides information on the soil biological quality – which is an indicator of land degradation. The fluctuation of the soil quality can be related to direct human inputs (including land management practices) or to long-term processes such as climate change. It is applied to the soil microarthropod community, separated according to the biological form approach with the intention of evaluating the microarthropods' level of adaptation to the soil environment life, and overcoming the well-known difficulties of taxonomic analysis to species level for edaphic mesofauna.

Focusing on the presence of some characters of adaptation to soil, and not requiring the complex taxonomic identification to the species level, means that non-specialists can

use QBS-ar analysis also. In a short period of time (5 days) one trainee should be able to apply QBS-ar in all the protocol's phases. It is a good candidate index for continuous biomonitoring of soil communities to describe patterns and processes in the microarthropod biodiversity across the landscape. A deeper knowledge of soil biodiversity in response to landscape use will provide guidance in effective management planning for sustainable renewable resource use and nature conservation.

QBS-ar has been developed by an Italian team (Parisi et al. 2005) and has been tested in several sites across Italy (e.g. Blasi et al. 2008, Hartley et al. 2008, Menta et al. 2008, Madej et al. 2011) for testing the effects of forest cutting, grazing, trampling, industrial activities, emission, agriculture, heavy metals and other anthropogenic affects. It does not require identifying the fauna to the species level.

METHODOLOGY

Soil organisms are separated into biological forms according to their morphological adaptation to soil environments. Each of these forms is associated with a score named the EMI (eco-morphological index), which ranges from 1 to 20 in proportion to the degree of adaptation. The QBS-ar index value is obtained from the sum of the EMI of all collected groups. If in a group, biological forms with different EMI scores are present, the higher value (more adapted to the soil form) is selected to represent the group in the QBS-ar calculation. This choice is based on the consideration that the examined soil is able to support well adapted and consequently more vulnerable biological form. Parisi et al. (2005) provides tables to easily calculate the index.

Frequency

In natural and semi-natural conditions the protocol should be completed every year, at the same period of the year, since the composition of the soil fauna partially varies with the seasons. The winter should be avoided, cold temperatures reducing the activity/presence of the soil fauna. In stable conditions it is adequate to collect the soil sample once a year (e.g. in the woods, grasslands). When the soil conditions change during the year (e.g. in agriculture ecosystems), the protocol can be completed for every season. In agricultural ecosystems, soil fauna composition and density vary in relation to tillage, crop rotation, organic matter management. In these cases, it is more desirable to collect the soil samples during the last period of cultivation (when the soil fauna community is less disturbed and the organic matter content is higher).

Data should be entered on for each identified spatial unit.

Measurables

- Order or class level of soil fauna collected (Recognizable Taxonomic Unit).
- Abundance per RTU.
- Scoring of each fauna group depending on its adaptation level to life in the soil.
- Soil biological quality index – calculated by adding the scores.

Materials

- For sample: soil corer, plastic bags, and labelling equipment.
- For extraction (Berlese-Tüllgren funnel): spotlight 40 W, large funnel, mesh (size 2 mm), and collecting vessel with preservative liquid (e.g. 2 parts 75% ethanol and 1 part glycerol).
- For identification: microscope, petri dishes and other vessels, pliers... and identification key.

Data capture

In each site for each unit, three soil cores, 100 cm^2 and 10 cm deep are collected in spring or in autumn, with a standard soil corer. Only soil is taken, the litter layer is removed before sampling.

The soil samples are sealed in polyethylene bags and are transported to the laboratory within 48 hours. A Berlese-Tüllgren funnel is used for microarthropod extraction, the specimens are collected in a solution of 75% alcohol and 25% glycerine by volume.

The extracted specimens are observed under a stereomicroscope and identified at different taxonomical levels: classes for miriapoda (*Diplopoda*, *Chilopoda*, *Symphyla*, *Pauropoda*) and order for insects, chelicerata and crustacea. The organisms belonging to each biological taxon are counted in order to estimate their density at the sampled depth (0-10 cm), and to relate the number of individuals and the sample area to 1 m^2 of the surface (ind/m^2).

According to the QBS-ar grid, each taxonomic unit is given a score named the EMI (eco-morphological index), which ranges from 1 to 20 in proportion to the degree of adaptation. The QBS-ar index value is obtained from the sum of the EMI of all collected groups. If in a group, biological forms with different EMI scores are present, the higher value (more adapted to the soil form) is selected to represent the group in the QBS-ar calculation.

QUALITY ASSURANCE

The operation requires trained personnel for the 3 steps of the protocol: soil sample, animal extraction, identification of groups and scoring.

EQUIPMENT

The extraction of fauna from the soil sample by a dynamic method requires an extraction funnel: the Berlese-Tüllgren extractor (Berlese, 1905; Tullgren, 1918) can be built easily according to a common protocol (Southwood, 1994). The respect of the protocol for the extraction stage is particularly important for the quality assurance of the indicator.

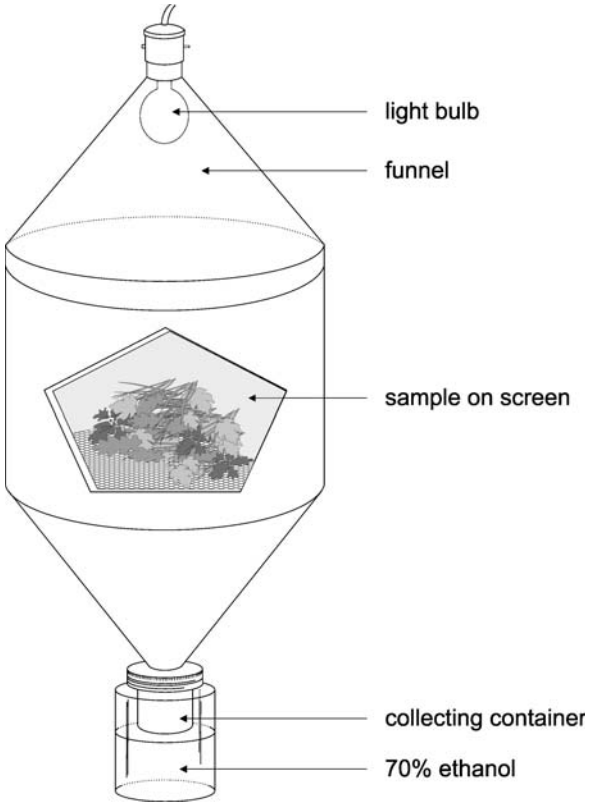

Figure 2. Design of the extractor
http://www.ars.usda.gov/main/docs.htm?docid=10141&page=2

A simple scheme to calculate collembolan's EMI:

1. Clearly epigeous forms: middle to large size, complex pigmentation present, long, well developed appendages, well developed visual apparatus (eye spot and eyes).
2. Epigeous forms not related with grass, shrub or trees well developed appendages (possible), well developed setae or protective cover of scales, well developed visual apparatus.
3. Small size – though not necessarily – forms, usually limited to litter, with modest pigmentation, average length appendages, developed visual apparatus.
4. Hemi-edaphic forms with reduced number of ommatidia, scarcely developed appendages, cuticle with pigmentation.
5. Hemi-edaphic forms with reduced number of ommatidia, scarcely developed appendages, often short or absent furca, pigmentation present.
6. Eu-adephic forms with no pigmentation reduction or absence of ommatidia, furca present – but reduced.
7. Clearly eu-edaphic forms, no pigmentation, absent furca, short appendages, presence of typical structures such as pseudo-oculi, developed postrantenal organs (character not necessarily present), apormorphic sensorial structures.

Table 2. Scoring tables (from Parisi et al. 2005)
Eco-morphologic indices (EMIs) of edaphic microarthropod groups

Group	EMI score	Group	EMI score
Protura	20	Diptera (larvae)	10
Diplura	20	Other holometabolous insects (larvae)	10
Collembola	1-20	Other holometabolous insects (adults)	1
Microcoryphia	10	Acari	20
Zygentomata	10	Araneae	1-5
Dermaptera	1	Opilioes	10
Orthoptera	1-20	Palpigradi	20
Embioptera	10	Pseudoscorpiones	20
Blattaria	5	Isopoda	10
Psocoptera	1	Chilopoda	10-20
Hemiptera	1-10	Diplopoda	10-20
Thysanoptera	1	Pauropoda	20
Coleoptera	1-20	Symphyla	20
Hymenoptera	1-5		

Table 3. A Simplified scheme to calculate collembolan's EMI

Order	Description	EMI
Orthoptera	in general	1
	Grillidae	20
Hemiptera	mostly epigeous or root feeding forms	1
	Cicada larvae	10
Hymenoptera	in general	1
	Formicidae	5
Araneae	small forms, scarcely pigmented	5
	forms > 5 mm	1
Diplopoda	forms > 5 mm	5
	forms < 5 mm	20
Chilopoda	forms > 5 mm well developed legs	10
	forms < 5 mm	20

REFERENCES

Berlese, A. 1905. Apparecchio per raccogliere presto ed in gran numero piccoli Artropodi. *Redia* 2: 85-90.

Blasi, S., Balducci, L., Di Filippo, A., Menta, C., Leoni, A. 1998. QBS-ar: an index for the evaluation of human impact on the soil microarthropod community in forests of Central Italy. *http://www.efi.int/files/attachments/events/ac2008/poster blasi_balducci_difilippo_menta_leoni.pdf*

Bongers, T. 1990. The maturity index: an ecological measure of environmental disturbance based on nematodes species composition. *Oecologia* 83: 14-19.

Hartley, W., Uffindell, L.A., Plumb, A.N., Rawlinson, H.A., Putwain, P., Dickinson, N.M. 2008. Assessing biological indicators for remediated anthropogenic urban soils. *Science of the Total Environment* 405.

Herrick, J.E. 2000. Soil quality: an indicator of sustainable land management? *Applied Soil Ecology* 15: 75-83.

Hogervorst, R.F., Verhoef, H.A., van Straalen, N.M. 1993. Five-year trends in soil arthropod densities in pine forests with various levels of vitality. *Biol Fertil Soils* 15: 189-195.

Madej, G., Barczyk, G., Gdawiec, M. 2011. Evaluation of Soil Biological Quality Index (QBS-ar): Its Sensitivity and Usefulness in the Post-Mining chronosequence – Preliminary Research *Pol. J. Environ. Stud.* 20(5): 1367-1372.

Menta, C., Leoni, A., Bardini, M., Gardi, C., Gatti, F. 2008. Nematode and Microarthropod Communities: Comparative Use of Soil Quality Bioindicators in Covered Dump and Natural Soils. *Environmental Bioindicators* 3.

Parisi, V., Menta, C., Gardi, C., Jacomini, C., Mozzanica, E. 2005. Microarthropod communities as a tool to assess soil quality and biodiversity: a new approach in Italy. *Agriculture, Ecosystems and Environment* 105: 323-333.

Ruf, A. 1998. A maturity index for predatory soil mites (Mesostigmata: Gamasina) as an indicator of environmental impacts of pollution on forest soils. *Applied Soil Ecoogy* 9: 447-452.

Southwood, T.R.E. 1994. *Ecological methods*. Chapman & Hall. ISBN 0-412-30710-3.

Tullgren, A. 1918. Ein sehr einfacher Ausleseapparat für terricole Tierfaunen. *Zeitschrift für angewandte Entomologie* 4: 149-150.

van Straalen, N.M. 1998. Evaluation of bioindicator systems derived from soil arthropod communities. *Applied Soil Ecology* 9: 429-437.

SUMMARY INSTRUCTIONS

1. If the protocol has to be repeated on each site: it must be completed once a year at the same period of the year. The respect of the same sampling period is particularly important.
2. The protocol should be repeated for each spatial unit identified in the site with homogeneous management practices and soil type.
3. Soil sample: on each spatial unit, 3 soil samples are collected, with a square soil corer (surface 10 × 10 cm, depth 10 cm). Each sample is placed in a labeled plastic bag.

4. Extraction of fauna: each sample is processed in a Berlese-Tüllgren extractor, with 7 days of extraction (see below).
5 Identification of fauna: the soil fauna is sorted by RTU (order or class level). The absence/presence of the different groups must be recorded in the spreadsheet, as well as the number of individual per group.
6. Scoring: Each group is given its adequate scoring between 0 and 20, following the grid provided (see scoring tables below).
7. Final index: it is calculated in the spreadsheet by summing the scores.

Simplified Berlese Tüllgren funnel

- The soil sample is placed on a 2 mm mesh, in a large funnel.
- Under the funnel is the collection vessel, filled with preservative liquid.
- A 40 W spotlight is placed on the top of the installation.
- After 7 days, collection of the vessel containing the soil fauna.

Location in plot: random; GPS record.

Timing

Timing of sampling: time of maximum QBS – a year of working, finding the month of max. QBS, and after that it could be decided.

Soil sampling

- Depth of sampling: 0-10 cm.
- Device for sampling: square cylinder, from metal.
- Size of cylinder: 20 cm diameter, 10 cm high. If the cylinder is too big for sampling, then combine small samples (of 10 cm diameter).

Number of replicates: 3 per plot, in most representative ones.

Animal extraction

- Length of extraction: 10 days.

Storage

- Soil storage before extraction: maximum time – 28 hours.
- Storage temperature: 25 degrees, in a plastic bag with air.

SOIL ORGANIC MATTER – CARBON AND NITROGEN STOCKS

CARSTEN W. MÜLLER

ECOLOGICAL INTEGRITY INDICATOR

Matter storage.

MEASURABLES

Soil bulk density, carbon and nitrogen content.

PROTOCOL SUMMARY

This protocol captures a basic soil feature on site specific carbon and nitrogen cycling, the stocks of carbon and nitrogen in the soil. Thus, the obtained data is crucial for any kind of carbon and nitrogen balancing on a plot or field scale. There are a number of ways to select sample spots for sampling frequency and number of replications. Those always depend on the ecosystem and the specific research question.

The following recommendations are based on the comprehensive work given by Stolboyoy et al. (2005) “Soil sampling protocol to certify the changes of organic carbon stock in mineral soils of the European Union”, where the authors give recommendations for the certification of organic carbon stocks in mineral soils of the European Union. The sampling of organic layers is added to the protocol. These soil parameters together with soil moisture and nutrient availability deliver a crucial background for site evaluation.

KEYWORDS

Soil bulk density, carbon content, nitrogen content, total carbon, total nitrogen.

SCIENCE BACKGROUND

This protocol aims to explain the main requirements when collecting samples for soil C and N stock information. By obtaining this data, it will be possible to relate stand biomass, soil microbial biomass, root mass, etc. to reliable information about C and N con-

tent and especially C and N stocks in soil. The protocol includes the evaluation of the soil C and N content and the determination of the soil bulk density. A general overview on soil methods including soil organic matter is given in Pansu and Gautheyrou (2003) and Petersen and Calvin (1996). Principles of chemical carbon analyses are given in Swift (1996).

METHODOLOGY

Measurables, Site Selection and Frequency

C and N content

Principles of chemical carbon analyses are given in Swift (1996). The most used method to analyse C and N concentrations, and thus organic matter content is the lab based dry combustion. The content of total carbon and nitrogen is measured in bulk soil samples of the soil depth and or horizon of interest. Preferably, both top- and sub-soil horizons are included in the measurement, as the sub-soil comprises an important reservoir for organic matter mostly derived from root input. Furthermore, if applicable also the organic layer material (especially in forest ecosystems) will be analysed. The total C and N is analysed in duplicate of air dried samples via dry combustion using an elemental analyzer. The samples for C and N content analyses are taken as disturbed samples, air dried, sieved over a sieve of 2 mm mesh size and homogenized. For referencing the obtained C and N contents, a drying of soil aliquots at 105°C for 24 h is crucial.

When carbonates are present, e.g. pH over 7, a parallel carbonate destruction and inorganic carbon quantification has to be done. This can either be done by combusting the organic C at 450° for at least 4 hours, or by acid treatment using e.g. HCl.

Bulk density

Bulk density is crucial for all determinations of element stocks, either C or N or soil nutrients, etc. For bulk density measurements a known soil volume is taken to the lab and dried at 105°C for at least 24 h until constant weight. From the dry weight and the volume, the bulk density is calculated. For the determination of the volume and weight of the organic layer a "counting frame" (e.g. square frame 20 × 20 cm) is used to remove the total organic layer material within the frame. This material is taken to the lab in order to determine the weight of the total organic layer material after drying at 105°C to constant weight.

In mineral soils with low skeleton content, steel rings of 100 cm^3 are usually used to sample a known volume. At least 3 replicates per soil depth / horizon should be taken. Bulk density accounts by definition only for the fine earth (< 2 mm), thus after the weighing of the dry soil, the soil has to be sieved over a sieve of 2 mm mesh size. The skeleton (> 2 mm) has to be weighed. By assuming a medium density of 2.65 g cm^3 for the skeleton, this can be subtracted from the bulk density.

Site selection

On every site, bulk density and C and N stocks have to be seen as standard values. The spot at a research site, which has to be sampled depends on the heterogeneity of the ecosystem, whereas croplands are either homogeneous, mountainous forests are very heterogeneous. A rough estimate would be: for heterogeneous sites a lasting pattern for sampling (e.g. comparable rock content, slope inclination, distance to trees) can be chosen which is maintained in the future, but at least 3 replicated spots should be analysed.

Stolbovoy and co-authors (2005) recommended a grid sampling approach, using a template with 100 sampling points that have to be layed over a map of the sampling site. For sites with an area of less than 5 ha, 3 composite samples are recommended. The sampling points consist of a soil pit for soil morphology and bulk density evaluations, whereas the composite sample (C and N content, pH or CEC, etc.) is taken from 8 spots around the central soil pit. The sampling spots should be fixed and kept for re-sampling.

If a broader approach is envisaged, geostatistical approaches can be used. Thus, sampling on a grid with different sampling distances (nested sampling) with subsequent geostatistical evaluation is possible and would enhance information on site specific heterogeneity, which may also drive plant growth, etc. For geostatistical approaches high numbers of samples (> 100) can be necessary (Steffens et al. 2008).

Frequency

At the absence of human management, the bulk density is a slow changing variable. Thus, the measurement of bulk density can be done once every 5 years. But, if management takes place (e.g. heavy machines on cropland, water regulation in peatlands) and changing vegetation also is of interest, an annual determination of bulk density and especially C and N might be advisable. In fast changing environments a yearly measurement is envisaged, around the same time in the year (same month), in agricultural croplands at the end of winter and not directly after harvest. In agricultural treatments, especially tillage system trials, manure treatments, but also energy crop trials, the yearly determination is crucial to detect slight changes on the long term. Especially for croplands, a fixed sampling scheme with a date before soil amendments (compost or biochar addition) is crucial.

Sample handling

The samples for C and N analysis can be sampled as disturbed samples using a shovel or spatula, just ensuring to not mix soil material of different horizons or layers. A prompt air drying of the samples is suggested in order to slow down any mineralization processes. If there are no additional chemical analyses planned for soil organic matter composition, the soil can be oven dried at 105° for 24 h. The samples need than to be sieved and homogenized over a mesh of 2 mm size. Air dried samples are worth to store at a dry and cool place for future determinations of for instance chemical properties or the determination of 13C and 14C contents. The undisturbed soil samples from the steel rings/bulk density determination.

QUALITY ASSURANCE

The sampling has to be done in a cautious way with respect to the right soil depth/horizon and an accurate sampling of the known volume samples. The more replicates are taken, the better outliers can be avoided resulting from inaccurate sampling.

REFERENCES

Pansu, M., Gautheyrou, J. 2003. *Handbook of soil analysis – mineralogical, organic and inorganic methods*, Berlin–Heidelberg, Springer.

Petersen, R.G., Calvin, L.D. 1996. Sampling. In: D.L. Sparks, A.L. Page, P.A. Helmke, Rh. Loeppert (eds.), *Methods of Soil Analysis Part 3 Chemical Methods* 1-19. Madison, Soil Science Society of America.

Steffens, M., Kölbl, A., Totsche, K.U., Kögel-Knabner, I. 2008. Grazing effects on soil chemical and physical properties in a semiarid steppe of inner Mongolia (P.R. China). *Geoderma* 143: 63-72.

Stolbovoy, V., Montanarella, L., Filippi, N., Selvaradjou, S., Gallego, J. 2005. Soil Sampling Protocol to Certify the Changes of Organic Carbon Stock in Mineral Soils of European Union. 1-56, Luxembourg, Office for Official Publications of the European Communities.

Swift, R.S. 1996. Organic Matter Characterization. In: D.L. Sparks, A.L. Page, P.A. Helmke, Rh. Loeppert (eds.), *Methods of Soil Analysis Part 3 Chemical Methods*. Madison, Soil Science Society of America, pp. 1011-1071.

GREENHOUSE GASES EMISSIONS FROM SOILS

Gemini Delle Vedove, Carlo Grignani and Chiara Bertora

ECOLOGICAL INTEGRITY INDICATOR:

Matter output, Global Warming Potential.

MEASURABLES:

Soil emissions of GHGs: CO_2, N_2O and CH_4.

PROTOCOL SUMMARY

This protocol addresses the measurement of greenhouse gases (GHGs) soil efflux by soil chamber methodology. The GHGs considered are CO_2, N_2O or CH_4.

The two most used chamber based methods are presented: the Non-Steady-State Through-Flow System (NSS_TFS, also referred as closed dynamic chamber) and the Non-Steady-State Non-Through-Flow closed system (NSS_NTFS, also called closed static chamber). The difference between the two methods is related only to the presence (Through-Flow), or not presence (Non-Through-Flow), of an in-situ analyzer connected to the chamber by a closed pneumatic circuit. The NSS_TFS has the best performance in term of precision and accuracy, but the availability of in-field operated analyzer limits its use. At present time, only CO_2 soil efflux is normally measured using the closed dynamic system, thanks to the availability of low cost CO_2 IRGAs (Infrared Gas Analyzer).

N_2O and CH_4 fluxes are normally measured using closed static systems by collecting gas samples to be analyzed later in the laboratory. It is expected that all three gases will be measured routinely in-field (e.g. using NSS_TFS) once non dispersive close-path GHG analyzers become more affordable (Venterea et al. 2009).

The protocol considers the most important precautions to guarantee accurate measurements of soil GHGs efflux. These include:

- Chamber design and deployment.
- Sampling frequency and intensity.
- Data collection and quality check of flux calculations.

KEYWORDS

Closed-Chamber technique, static and dynamic chamber, CO_2, N_2O, CH_4, GHG soil fluxes, Soil Respiration, Trace Gas Emissions.

SCIENCE BACKGROUND

An increasing research effort into climate change and related effects on ecosystem responses has taken place in the last two decades. Moreover increasing research is deployed to evaluate mitigation and adaptation strategies both in forest, wetlands, grasslands and croplands. The three main trace gases responsible for positive radiative forcing (i.e. greenhouse gases GHG) are CO_2, N_2O and CH_4. Their balance in the atmosphere is related to burning carbon-based fuels (CO_2 from oil, coal, natural gas and wood), and also from a combination of land use and land use change imposed to terrestrial managed or natural ecosystems in recent centuries. C and N cycles are interrelated in the terrestrial ecosystems, and the warming climate could result a positive feedback effect on the net emission of C and N derived GHGs.

To understand ecosystem responses to climate changes, a major consideration is required of soil processes. Soil acts either as a potential sink or a source of GHG into the atmosphere. Soils store in the Soil Organic Matter, the largest C and N pools of terrestrial ecosystems, and the fate of such pools depends ultimately on the balance between processes controlling soil C and N inputs (i.e. primary production, belowground biomass allocation, littering, biological and industrial N fixation) and output (i.e. soil CO_2 efflux, N gaseous losses, erosion and leaching) (Chapin et al. 2012).

Stock change, flux measurement and modeling are all independent methods useful to measure and understand trace GHGs contribution to global warming (Smith et al. 2012).

C and N Soil balance could be measured in terms of stock changes in a long time interval (from years to centuries). Such measurement requires a high number of samples to satisfy accuracy and detection limits (see the related protocol in this document: **Soil Organic Matter – Carbon and Nitrogen Stocks**). This measurement gives superior estimates of net flux of CO_2 between soil and atmosphere, but doesn't allow for the contribution to global warming of the other soil emitted GHGs (i.e. N_2O and CH_4).

Flux methods measure out-flux of all trace GHGs from soils; the sum of fluxes in a time interval gives, in theory, the equivalent value of stock change. These methods have their own uncertainties and inaccuracies, but are the unique way to compute the short or long-term emissions balance for N_2O and CH_4. The flux methodologies allow to study in more detail undergoing processes and the effect of pedoclimatic and ecological drivers. Flux methods rely on chamber techniques (like those presented in this protocol) and in micrometeorological techniques (i.e. Eddy Covariance).

Modelling is the third approach used to estimate GHG emissions at territorial level and for climate or management scenario analysis. It requires measured data with appropriate calibration and validation. These data come from stock change assessment and or from flux data.

In this protocol we will analyse the flux method in detail considering in particular the closed chambers method. The closed chamber is a top-closed and base-open box placed on the soil surface. The chamber method relies on the measurement of increasing, or decreasing, concentration of trace gases of interest inside the chamber's headspace atmosphere. The time series data concentrations can be done in the field with an in-situ operated analyser, or with an analyser in the lab. In the first case, a trough-flow pneumatic close circuit (NSS-TFS) circulate continuously the air from the chamber headspace to the analyser and back to the chamber. In the second case, there is not a through flow (NSS-NTFS), rather a limited number of chamber's air samples is collected manually in the field and analysed later in the laboratory.

The soil trace gas effluxes display high spatial and temporal heterogeneity. As an example, the soil CO_2 efflux (called also Soil Respiration) varies in response to soil temperature, soil water content and photosynthetic C input. Also soil variables, like soil texture, bulk density or soil organic matter quantity and quality could affect Soil Respiration in both short and long terms (Conant et al. 2011). Fast responses of all GHGs soil effluxes are associated with soil or ecosystem disturbance: snow melt, fire, tillage, fertilization, flooding, irrigation, harvest are some examples of natural or human induced disturbances.

In order to handle spatial and temporal heterogeneity, the best option involves either a portable system or a long-term, multi-chamber, automatic measurement system. The portable system is suggested to cover spatial variability amongst many ecosystems, and the automatic one is used to follow temporal variations of fluxes. Both are based on closed dynamic chambers (i.e NSS_TFS) (see Savage et al. 2003). The NSS-TFS are labour saving and are in practice more precise than closed static chambers systems due to: lower error associated with sample handling, and higher number of data points per measurement and a shorter deployment time (DT) (see Heinemeyer et al. 2011, Rochette et al. 2008, Livingston et al. 2006).

The GHG fluxes can be measured quickly (5-20 min per chamber measurement) by using closed dynamic chambers. Reducing the time during which the chamber is closed over the soil (i.e. DT), minimizes the unavoidable alteration of diffusion path caused by the increasing concentration of trace gas in the closed chamber's headspace. Another advantage of using NSS_TFS, i.e. using in-situ analyzers, is that many data of trace gas concentrations can be logged for each measurement, which can be used to fit appropriate regression functions with increased precision.

NSS_TFS are normally used only for Soil Respiration (CO_2 efflux), measurements, due to the availability of low cost and reliable IRGAs.

The uncertainties are high for dynamic chambers measuring N_2O and CH_4 effluxes, when in-situ operated analyzers do not guarantee a sufficient precision and detection limits (Livingston, 2006; Parkin et al. 2012). For these trace gases closed static chambers (NSS_NTFS), is still the suitable method in practice (Rochette, 2011; Heinmeyer, 2011, Parkin and Venterea, 2010; Venterea et al. 2009). In this system, as noted above, air samples are taken manually from the chambers' headspace to be analysed later in

the laboratory. The manual collection of air samples limits their number and influences the duration of chamber closure (Deployment Time DT). The choice of DT depends mostly on analyzer precision. In this context Parkin et al. (2012) gives a useful rationale to identify the best DT, especially with CH_4 and N_2O flux measurements.

METHODOLOGY REVIEW

Livingston and Hutchinson (1995) distinguished three different chamber techniques to measure soil trace gas effluxes: closed static system (Non Steady-State Non-Trough Flow System NSS_NTFS), closed dynamic system (Non Steady-State Trough Flow System NSS_TFS) and open dynamic system (Steady-State Trough Flow System SS_TFS). In the NSS types, the soil trace gas efflux is estimated measuring the initial concentration change rate (dx_0/dt) inside the headspace of the chamber (a top-closed and bottom-open box), when placed on the soil.

In the Steady-State chambers the flux is calculated from the difference in trace gas concentration between the air flowing, at a known mass rate, through the chamber inlet and outlet after the chamber headspace stay at steady state concentration close to external air concentration. But also in this case it's difficult to attain conditions not disturbing the diffusion/advection process at the soil-atmosphere interface.

At the moment, no single method has been established as a standard (Pumpanen et al. 2004), but the closed chamber systems are the most used.

The Non-Steady State chamber based systems are all potentially biased by altered soil trace gas diffusion gradients, which lower the rate of diffusion process from soil to closed chamber headspace. Concern are also for pressure differentials between chamber and outside air induced by wind or by any advective flux perturbation (i.e. change in chamber temperature, the pump circulating the sample air to analyzer, fan or other mixing device inside the chamber not well positioned or operated too fast, wind effect).

The altered diffusion gradient problem can be described by the first Fick's low, which states that flux is dependent on the concentration gradient and the diffusivity (related mainly to the air-filled porosity) of the soil. Therefore, as the trace gas concentration (x_c) within the chamber headspace increases, the diffusion gradient decreases, the tracing of headspace concentration reaches an asymptote and the dx_c/dt begins to decline. If one considers constant dx_c/dt (i.e. using linear regression) over the measurement period, he is negating the feed-back of gas concentration increase and diffusion in the closed chamber. This results in an underestimation of the real flux (i.e. before chamber deployment over the soil). This underestimation is high (up to 40%) and proportional to the "true" pre-deployment efflux (see Livingston et al. 2006; Venterea, 2010; Parkin et al. 2012).

In order to show the process of diffusion in the non-steady state chambers, let consider an example of operation and data collection of concentration with a NSS_TFS; it is representative also for NSS_NFT chambers. The measurement time in NSS_FTS is the time interval during which GHG concentration data (and related data on air temperature pressure and water vapour) are measured and recorded. This measuring/recording

interval starts from 10 sec before chamber closure and ends when the chamber reopens. After the lid or the chamber seals (i.e. closure time) the air volume above the soil area covered by the chamber (sealed on a base or collar inserted to the soil), there is a time interval allowing steady mixing of the air flowing in a pneumatic closed circuit, from the chamber headspace to the analyzer and back to the chamber. The mixing interval has to be experimentally defined and depends on the pump flow rate, on the total volume and on site characteristics. The mixing interval ranges from 10 to 30 seconds using a volume of 2 L and a flow rate of 800 ml/min and a tubing length of 10 m connecting chamber and analyzer. Note that mixing time is present only in Trough Flow Systems (Dynamic Chambers).

After the mixing interval, the time series (interpolation interval) of GHG headspace concentrations is used for flux computation. To be able to compute fluxes using different approaches, it is mandatory to collect all the following data from before chamber closure, to the end of interpolation time. Because our aim is to estimate the flux before chamber closure time (i.e. avoiding the disturbance of chamber on diffusion process), the initial pre-deployment conditions (before chamber closure) of trace gas concentrations must be assessed. Moreover initial temperature, water vapour and pressure are used to compute dry air molar density ρ_0 (mol·m^{-3}) and the pre-deployment rate of change dx_0/dt (see computation below).

There are different approaches to derive the so called pre-deployment fluxes F_c. The following eq. 5 takes into account the time delay required to have steady mixing conditions in a closed chamber (Welles et al. 2005).

$$F_c = \rho_0 \cdot (1 - x_{v0}) \cdot \frac{V}{S} \cdot \frac{dx_{c0}}{dt} \qquad \text{eq.1}$$

$$\rho_0 = \frac{P_0}{R \cdot T_0} \qquad \text{eq.2}$$

$$x_c = \frac{x_{analyzer}}{1 - x_v} \text{ water vapor diluition correction} \qquad \text{eq.3}$$

$$\frac{dx_{c0}}{dt} = A \cdot (C_x - C_{ini}) \text{ derivativeat time } t = t_0 \text{ from} \qquad \text{eq.4}$$

$$x_c(t) = C_x - (C_x - C_{ini}) * e^{(-A \cdot (t - t_0))} \qquad \text{eq.5}$$

$$x_c(t) = b \cdot t + q \text{ Linear regression} \qquad \text{eq.6}$$

$$\text{where } \frac{dx_{c0}}{dt} = b$$

where F_c is the pre-deployment flux of the element x (mol · m^{-2} · s^{-1}) computed at time t equal to t_0. Parameter t_0 (units in seconds) is estimated by non-linear fitting procedure; it represents the delay during mixing time starting from chamber closure to time steady increase of concentration inside chamber. ρ_0 is the bulk air density (mol · m^{-3}), x_{v0} is molar fraction of water vapor (mole · mole^{-1}) so $\rho_0 \cdot (1 - x_{v0})$ is dry air molar density (mol · m^{-3}) at t_0 time; P_0 (Pa) and T_0 (K°) are atmospheric pressure and chamber headspace temperature at time t_0, R is the molar gas constant (8.31 m^3·Pa·K^{-1} · mol^{-1}). V and S are total system volume (m^3) and area (m^2) of soil covered by the chamber. So V/S is the apparent chamber height ($h_{chamber}$ (m)). The derivative dx_{c0}/dt (mol · mol^{-1}·s^{-1}) (eq. 4), is the rate of change of the element (trace gas) water-corrected for dilution into the chamber at the instant before the analyte's concentration rise after chamber close ($t = t_0$). This slope can be computed by fitting empirical model using measured subsequent concentration values, $x_c(t)$ as dependent variable and time (t) as independent variable (eq. 5). Note $x_c(t)$ are the water-corrected molar fraction in dry air of element x computed as in eq. 3.

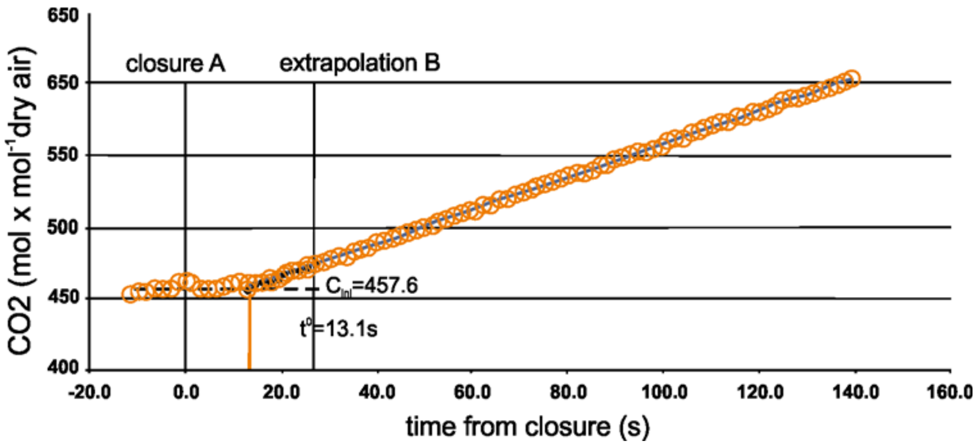

Figure 3. Principle of the measurement applied to the soil respiration (CO_2 efflux) data. Chamber start closing 140 s before closure time (time 0 = Closure A in the graph) and stays closed for 140 s. The initial [CO_2] Cini is the average of the 16 s closure time. The mixing interval is 27 s (B-A) and allows establishment of a steady mixing in the closed circuit between chamber headspace and analyzer. After mixing time interval, the [CO_2] data shows an increasing trend. This increase has a small non-evident asymptotic trend due to the 'chamber effect' on the diffusion process governing trace gas soil emissions. The non-linear regression is done over the interpolation interval lasting, in this case, 106 s. The total time interval required to get a flux in this example sums 280 s from closure start to the chamber reopening. Open symbols are measured CO_2 concentrations; blue line are values from the computed non-linear regression: CO_2_dry = $C_t = C_x - (C_x - C_{ini})*exp(-A*(t-t_0))$. The regression parameters are C_x= 6942, A = 18.20 · 10^{-5} and t_0 = 13.1. CO_2-Efflux=3.2 $\mu mol \cdot m^{-2} \cdot s^{-1}$. Linear regression on the same data set results in a CO_2-Efflux = 3.18 $\mu mol \cdot m^{-2} \cdot s^{-1}$ (-1.4%). Red closed circles are extrapolated data points of the non-linear fitting into the mixing interval up to $t+t_0$ time.

The fitting parameters of eq. 5 are C_x, A and t_0. C_{ini} is the initial molar fraction computed as the intercept of the $x_c(t)$ or as concentration mean at chamber closure (10-15 seconds around chamber closure time). Cx is the asymptote and represents the concentration at the soil-air interface. A is the parameter which defines the curvature: positive if $C_x > C_{ini}$ or negative otherwise. t_0 is the third non-linear regression parameter, it represents the time when $x_c(t)$ is equal to C_{ini}.

Using the same data coming from one interpolation time interval, it is possible to compute flux with different non-linear approaches (like NDFE suggested by Livingston et al. 2006) or using linear regression (e.g. eq 6) or quadratic parabolic function.

The error (measured vs. true flux) could derive from the corruption of the assumptions made by the model used to compute the real dx_{c0}/dt. One important assumption is that no leaks, radial leaks in particular, are occurring. Area/perimeter ratio, insertion depth, total Volume/Area and deployment time influence in different ways the radial leaks beneath the chamber base (Healy et al. 1996). The area/perimeter of a circular chamber is equal to radius/2 and the circular chambers have an area/perimeter ratio higher than rectangular chambers by a factor of 1.13 = 2· (pigreco)$^{-0.5}$. In theory, assuming the same area, circular chambers have less radial diffusion beneath the chamber/collar perimeter than

rectangular one. The value of area/perimeter >10 suggested by Rochette et al. (2008), implies for NSS chambers a diameter of 40 cm for the cylindrical chamber (resulting in an base area of 0.125 m^2) and a basal area of 0.161m^{-2} for rectangular chambers.

If the deployment time is short, let say less than 5 min as is the case of normal operations of dynamic chambers, the radial leaks is supposed to be of less extent in comparison with longer deployment time typical of closed static systems (20-60 minutes). Remediation for radial leaks are: increase insertion depth (see Hutchinson et al. 2001 table 2); reduce measurement time or increase $h_{chamber}$. Remember that high values of soil air filled porosity, like the presence of clods or stones, increases the risk of leaks markedly. In these cases, the risk can be reduced by adding some fine textured soil (2 mm sieved) to the base of the collar.

Errors can also originate from advective fluxes, and leakages from the chambers not explicitly considered in the model used to interpolate the time course of concentration. Advection (i.e. mass flow) introduces uncertainty in data check and quality assessment. The best approach is to use best practice to reduce leakages from chambers. In this case the NSS_FTS system is again more powerful, mainly because the deployment time is reduced and so the chamber disturbance on the diffusive gradient is less important (Healy et al. 1996). It is possible to check for sudden and unreasonable changes in the flux using NSS_FTS when employed for long term automatic flux measurement. Checking for instance the change of fitting parameters values of the $x_c(t)$ function (i.e. eq. 5 and A parameter in particular), one can relate sharp changes to sudden changes of environmental conditions (i.e. soil moisture, soil temperature, wind or atmospheric pressure). An approach to evaluate the advective and diffusive effects controlling the headspace concentration $x_c(t)$ is presented by Welles et al. (2001) and a modeled application (NDFE) in non-steady state chamber is presented also in Livingston et al. (2006) and Venterea (2010)[16].

Another aspect related to the closed chamber methodology is the precision of measurements depending on the analyzer precision (i.e. CV% = standard deviation of measurements/mean of a fixed air sample concentration). The analyzers use a correction for cell-temperature (cell is usually thermo stated at high temperature) and cell-pressure in order to output the mole fraction value (mole of trace gas · mole of bulk air^{-1}). The molar fraction is a conservative unit to changes in pressure and temperature. Moreover InfraRed Gas Analyzers (IRGA) used for CO_2 and Photo-Acoustic infrared Spectroscopy analyzers (PAS) used for all trace gas (CO_2, N_2O and CH_4) are calibrated to correct, in different ways, for overlapping absorption spectra (the so called band-broadening effect) of water vapour and/or other analytes. This effect, if not accounted, adds a positive error to the real molar fraction data.

16 Note: there is not an optimal computation method in terms of accuracy and precision of flux measurements. Venterea (2010) outlined that the best accuracy is achievable with non linear methods, and NDFE (Livingston et al. 2006) was the best even if it has simplifying assumptions about soil properties uniformity (see Venterea et al. 2008) and horizontal leaks (Pedersen et al. 2008).

For in-field measurements of N_2O and CH_4 with PAS, the corrections for band-broadening spectra of CO_2 and water vapor are very crucial. But recently (see Iqbal et al. 2012) the accuracy and precision (CV%) of PAS were tested vs lab GC analysis assuming as true value a NIST Certificate. The accuracy and precision of PAS were within the range of 0.5% to 8.8% and 1.2% to 2.5% respectively and these values are in the range of lab GC accuracy and precision values (Iqbal et al. 2012).

The water vapour entering the chamber headspace (due to soil evaporation) can create an underestimation of concentration due to its dilution effect. This is important when low GHG efflux and high soil evaporation are present at the same time. When a chamber is placed on a moist soil, the water vapour molar fraction increases due to soil evaporation, and it displaces some of the chamber air trough vent (assuming pressure and temperature remain constant); thus a dilution of the trace gas concentration occurs, causing the rate of trace gas concentration (*dx/dt*) into the chamber to appear less than it really is. For example, this dilution effect of water vapour on the measured flux is responsible of an error (underestimation) of the soil CO_2 efflux ranging from 2 to 4%, when the water vapour concentration in the headspace air is ranging from 20 to 40 mol·mol^{-1} of air and the soil respiration is between 1 and 8 μmol·m^{-2}·s^{-1}. In order to overcome such underestimation the molar fraction (i.e. mol·mol^{-1}) in the dry air is a preferable concentration unit. This is obtained by the following correction $x_{c\_dry} = x \cdot (1 - x_v)^{-1}$, in which x and x_v are molar fraction for analyte and water vapor (mol·mol^{-1} of wet air) respectively; so $(1 - x_v)$ is the mole fraction of dry air (mole of dry air · mol^{-1} of wet air).

In terms of precision, (low CV% in measured flux) linear (LR) models are more powerful. So if one is interested in relative comparisons among treatments LR is preferable; if interested in absolute value accuracy nonlinear models are giving best results (Venterea, 2009; Pedersen et al. 2008; Kutzbach et al. 2007; Parkin et al. 2012).

MEASURABLES

Flux estimation needs a number of trace gases (GHGs) molar fractions (mol·mol^{-1}) samples collected from a chamber headspace over a convenient time interval. In the static chambers (NSS-NFTS), a few samples are collected in the field and measured in the lab (Gas Chromatographic techniques GC or Photo-Acoustic infrared Spectroscopy analyzers PAS). In Through-Flow dynamic chambers (NSS-TFS) the air samples are analyzed in the field using portable analyzers.

The flux is calculated using the gas concentration vs. time relationship in a curve fitting procedure (linear or, preferably, non-linear function). Each flux of the GHG of interest is typically referred as μmol GHG·m^{-2}·s^{-1}. For N_2O and CH_4 a more convenient unit is nmol·m^{-2}·s^{-1}. The use of [μmol GHG·m^{-2}·s^{-1}] allows conversion to different mass units (e.g. g or kg of GHG), area units (m^2 or ha) or time units (second, hour, day or year).

During measurement time the following variables must be time-referenced (date and time; time must be always in Local Standard Time) and it is important to know and possibly to standardize the units of all variables entering in the common database:

- Plot and Chamber (both described in a separate data-tables describing plot treatments and chamber characteristics, including insertion depth, chamber Area (S) and chamber Volume (V) and having same dimension units (i.e. m).
- GHG mole fraction ($\mu mol \cdot mole^{-1}$) in dry air: the analyzer gives the mole fraction corrected for any band broadening and for cell temperature and pressure, convert this mole fraction in bulk air to dry air using the air water vapour molar fraction as seen in computation.
- Air temperature, air humidity and pressure of the chamber headspace at closure time in order to compute number density of dry air ($mol \cdot m^{-3}$) as required for the computation on mass basis GHG flux at pre-deployment time ($\mu mol\ GHG \cdot m^{-2} \cdot s^{-1}$) (see computation).
- Soil temperature (at 2.5 and/or 5 cm) and soil moisture (0-15 cm) representing the area covered by the chamber should be recorded preferably in duplicate at the time of measurement for each plot. These data are used to filling gaps between measurements interval of GHG fluxes ie using the sensitivity response of trace gases fluxes to soil temperature (e.g. Q_{10}). They are used also for the advanced analysis of quality assurance of flux data as suggested by Venterea (2010). Check if the temperature and soil water content data are the same outside and inside the chamber; if not there is some unwanted effect due to collar or to chamber installation. The assumption that the chamber deployment does not influence these parameters (i.e. soil temperature and soil water content) is related to a correct installation of chamber minimizing interferences with soil microclimate. Soil temperature measuring depth is strongly influencing the estimate of cumulative trace gases efflux based on Q_{10} (see Parkin et al. 2003). The best soil temperature depth used to estimate the daily and seasonal cumulative fluxes depends not only on spatial and temporal variable production of trace gases, but also on soil characteristics (ie texture, bulk density, soil water content), which influence diffusion and, in some occasion, mass transport of trace gases into the soil profile. In natural undisturbed ecosystem (like a forest floor) the trace gas fluxes vs. soil Temperature relationship are more precise as those eco-systems are more stable on daily and seasonal basis.
- In conjunction with N_2O efflux measurements, soil content of nitrate and ammonium (0-10, 10-20 cm) are measured. It is desirable that soil nitrate and ammonium concentration be determined throughout the year at appropriate defined time intervals depending mostly on management events (like fertilization in agricultural soils), and on rain or irrigation events, which strongly determine the denitrification process.

FREQUENCY OF MEASUREMENTS

Trace gas fluxes exhibit a high degree of temporal variability. The frequency of measurements has different consequences for different trace gases. Temporal variability using an automated SR system is managed easily. The adequate daily frequency for

automated chambers could be every 1 to 6 hours. Higher frequency is useful in the context of sudden changes in microclimate or episodic high disturbance events. The frequency question is more of an issue for manually operated, dynamic chambers or static chambers.

Temperature, rainfall and photosynthesis (e.g. for soil co2 respiration) are the main environmental factors controlling trace gas emissions in natural undisturbed ecosystems, whereas in managed agricultural system fertilization, tillage, harvest are additional drivers modulating trace gas emissions. Thus, the more frequently measurements are made, the more accurate the integrated seasonal/yearly cumulative flux estimate will be (Parkin, 2008).

Ideally, one should estimate the diurnal, weekly and monthly variability (e.g. coefficient of variation CV%) present in the ecosystem of interest and judge the effect of such variations on the magnitude of the effluxes. For temperature and photosynthesis the expected variability is higher on daily basis, but the change on weekly or monthly basis is more smoothed. So after having defined the best time of the day representing the average daily flux value, and after having tested a temperature sensitivity (Q_{10}) model for each trace gas, one can use such relationships to estimate daily average flux values and then compute the daily cumulative flux for long time intervals. Rainfall, thawing, litterfall and management activities, on the other hand, are events that influence in the short time the efflux of all trace gases so take care to monitor such flux peaks (positive or negative) expected the days following such events. The error of cumulative fluxes is higher when absolute high peaks are ignored.

In forest ecosystems Savage et al. (2008) estimated that the manual sampling of soil respiration, with portable NSS-TFS, at weekly or biweekly intervals (between 9:00 and 15:00 h) and in non-raining days, has the probability of 90% of cases to yield a value having +/- 10% of error compared to continuous automated system.

On N-fertilized cropland, Parkin (2008) reported that once every 3 day frequency is necessary to achieve +/- 10% uncertainty of the true seasonal cumulative N_2O efflux in more than 80% of the cases. Sampling every 6-8 days, the probability of obtaining estimates of true cumulative N_2O losses with a precision of ±10% were 70% for the between-band chamber's locations and only 20% for the over-bands fertilized locations. This is explained by the higher variability and higher amplitude of N_2O emission's peak values of over-bands N fertilized locations.

Recommendations

In natural ecosystems the manual system (NSS-NTFS static chamber) can be used with a frequency of 15 days to monitor CO_2 efflux, and for N_2O efflux, may be, lower frequency is also adequate. In any case, care must be reserved to undertake a preliminary intensive test to evaluate the best time of the day for manual sampling, as this impacts both the organization and the sample size (number of measured points). In disturbed systems, like in arable crops, the ideal frequency is much higher, especially after events

likely to influence trace gas emissions. In this case an automatic system should be considered, given the precision attainable and research costs.

SITE SELECTION

The location of sampling points is to be decided on the basis of research objectives. Consider that soil is highly variable, and so a randomized block (>= 3 blocks) with at least three replicates (automatic chambers or collars for manual chambers) per plot for each treatment could be a good experimental design in many factorial experiments. When the interest is on measuring a site, a stratified or grid sampling scheme is adequate. The use of GPS is encouraged in order to relocate the measurement points. Metal plates give a more precise localization of the points inside a large undisturbed site area, allowing them visited at very long times intervals (e.g. once per year).

Site selection problem depends on the choice of measuring the total soil respiration or only the heterotrophic soil respiration. In the latter case, techniques are available in order to avoid autotrophic fluxes like those coming from rhizosphere and root respiration. The technique often used to measure it is the 'root exclusion'; it is performed by a cylinder deeply inserted into the soil surrounding the collar. In this way you are sure that no growing roots are beneath the measurement point. The external cylinder insertion has to be done long time before the measurement in order to minimize the effect of the cylinder insertion. For more details on definition and methods to measure heterotrophic and autotrophic respiration see Kuzyakov et al. (2005), Kuzyakov (2006), Subke et al. (2006) and Chapin et al. (2006).

METHODOLOGY

As outlined in the methodology review the soil efflux of trace gases is made with closed chambers systems, dynamic or static. Both types of systems can be used for measure GHG soil effluxes, but in practice the dynamic systems are used to estimate soil respiration (i.e. CO_2 soil efflux) and static systems to compute soil N_2O or CH_4 net efflux.

Dynamic Chambers (NSS-TFS)

In dynamic chambers the basic equipment is: a chamber with (in case of unattended automatic systems) or without a motor to accommodate the chamber over the collar, or base, inserted into the soil at a convenient insertion depth; a vent to maintain equilibrium with external pressure variation, an analyzer suitable to operate in field conditions, a pump to circulate in a close pneumatic circuit the air from the chamber to the analyzer, and a control unit (a datalogger) to operate all devices (chamber closure motor, pump, valves and analyser). The control unit records analyzer's and environmental sensors' outputs during and, for automatic chambers system, between chamber's measurements.

For automatic unattended systems, it is strongly suggested to add a communication device (like a GSM modem) and software able to monitor the in-field system from the office desk. This allows a frequent downloading of the huge amount of data logged by the control, unit and remotely check or control the system operating in the field. Batter-

ies, Photovoltaic solar panels, or any other Direct Current supply is required to power the system (12V and 0.5-2A are normally required with one IRGA-CO_2 system).

The dynamic chamber system requires some expertise in choosing all the system parts listed above, though there are commercially available solutions for portable and/or automatic unattended multiple chambers systems. These are generally equipped with portable IRGA systems for CO_2 measurements (e.g. try a Web Search: "soil respiration system"), but also manufacturers of other kind of trace gas analyzers will give suggestions for applications in trace gas in-field monitoring systems. These systems are basically similar in how they function, and are supported by instructions for installation and maintenance, and by dedicated software for data collection and management (note, the software for flux computations can be different). The automatic chambers' cost is more than that of portable systems, so there is often a trade-off between cost and benefit that depends on the precise research objectives.

NSS-TFS Chamber Design and construction

As an example we present some details of a dynamic chamber system (NSS-TFS) made by University of Udine (see Delle Vedove et al. 2007 for more details) and used for automatic multi-chamber soil respiration measurements in twelve chambers.

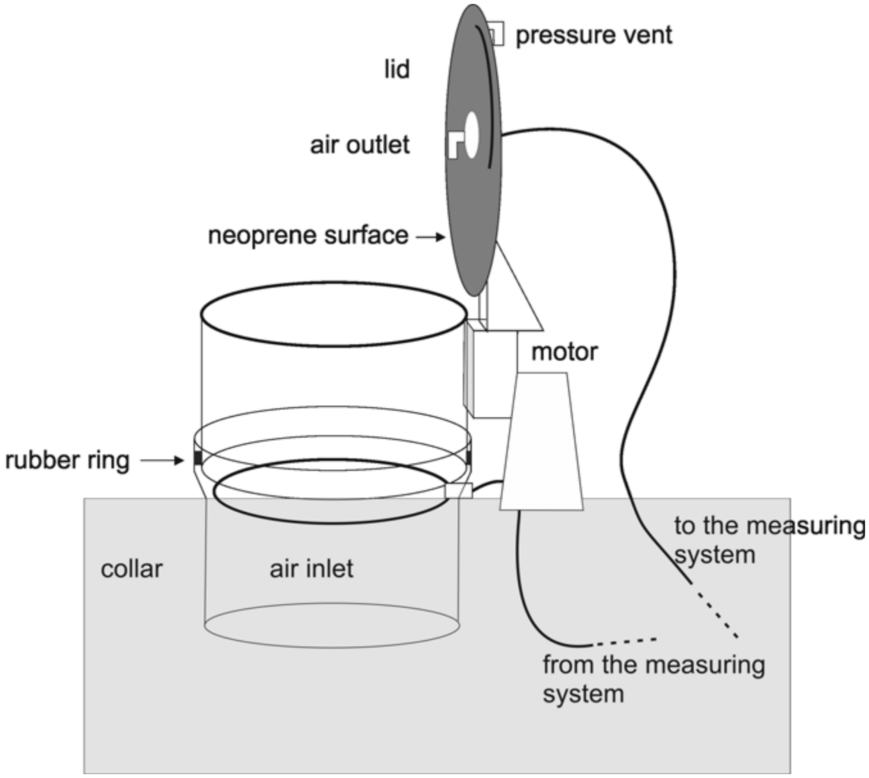

Figure 4. Schematic of chamber's parts with collar inserted into the soil

Each chamber consists of a steel collar (20-30 cm of diameter and 8-12 cm height) and a DC motor (IP 56) for opening and closing the steel lid. The closing must be slow in order to minimise pressure perturbations at closure; in this example the lid takes 140 s to completely close. The chamber is placed on a steel collar inserted into the soil (at 2-5 cm) and the lid, when open, is in vertical position on North side of the collar to avoid shadowing. The seal of the chamber is ensured by a neoprene closed cells sheet on the inner surface of the lid, which is sticking a rubber ring placed on the top perimeter of the chamber when the chamber is closed. The bottom chamber's perimeter is inserted into the collar and another rubber ring prevents leaks from the collar/chamber perimeter junction. The air is sampled from the centre of the lid and is returned by a high density PE circular tube placed inside the chamber above the soil. This pipe is perforated with holes having a diameter of 0.5 mm and spaced 1 cm each other. The horizontal air flow (0.6-0.8 L/minute) generated by the pump and exiting from the perforated circular pipe, creates a gentle mixing of the air inside the chamber. The pneumatic circuit between the chamber and the measuring system is made of high density PE tubing (up to 10-15 m long, 4/6 mm inner/outer diameter). To avoid any pressure change induced by advection phenomena inside the chamber or any wind induced pressure difference between inside and outside the chamber, a pressure vent is placed aside the chamber. The vent is connected to the chamber with the same PE tubing long 15-20 cm. The vent is made of two plates according the indication of Xu et al. (2006) and Hutchinson and Livingston (2001). The adopted vent design allows static pressure changes inside the chamber to follow whatever static pressure changes occur in the surrounding air outside the chamber, both in calm and windy conditions, while remaining insensitive to wind direction.

The pneumatic circuit requires solenoid valves which are operated by a Control Unit (in this case a CR1000 data logger Campbell Sci.) to circulate the air sample from the Analyzer to the chamber. One chamber and corresponding 2 valves (inflow and outflow) are operated in sequence by the CU.

The analyzer is a SBA4 (from PP-Systems, USA), non dispersive IRGA-CO_2 (0-2000 ppm range and < 1% accuracy and precision). It is equipped with water, temperature and pressure sensors to make a correct dry air molar fraction measurement. The calibration is made once a year for the span, and before every chamber measurement the CU operate an 'auto Zero' feature using a CO_2 sodalime column scrubber. The IRGA is connected with a serial port to the CU and the data output interval is every 1.6 sec. This analyzer is adapt to operate in field conditions with minor maintenance needs and low power requirements (0.6 A at 12V).

Dynamic chambers used for in-field CO_2 efflux can also be used to measure flux data of N_2O and CH_4. Samples of air can be collected from the chamber closed lid or from the pneumatic circuit leaving the pump switched on. Three or more air samples can be collected in vials using needle (22AWG) and syringe of 20-30 ml. The needle is inserted in a butyl rubber stoppers or red rubber stoppers inserted in online T-fitting in the case of sampling from tubing. The glass vials (10-30 ml) used to store air samples have the

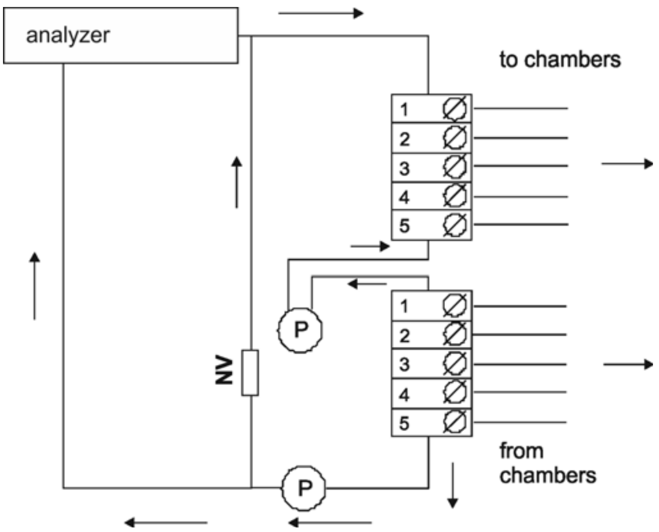

Figure 5. Scheme of pneumatic circuit of a multiplexed soil respiration dynamic chamber system. P = Pump, NV one way regulated valve, and manifold mounted 12 or 24 VDC operated valves

same crimped stoppers. For vials and sampling operations follow protocol recommendations for NSS-NFT systems. Care is required: the first air sample has to be taken as quickly as possible (less than 20 s) after chamber closure. CH_4 and N_2O require a longer deployment time than for CO_2, due to their lower flux rate. This could be in the range of 20 to 30 min. As noted above, this time influences the accuracy of the measurement, and the accuracy is related in a complex way to chamber height, deployment time, soil properties (e.g. soil air-filled water porosity), calculation method, the flux magnitude itself and to the analyzer detection limits (Venterea, 2010; Parkin et al. 2012). After taking care of these facts related to non-CO_2 fluxes (e.g. increasing insertion depth and chamber height), a dynamic system can be used as a static system. The sampling done on tubing T fitting one has a more small advantage: the first sampling measurement (at chamber automatic closure) is not disturbed by the breathed air or the presence of the operator close to the chamber.

Static Chambers (NSS-NTFS)

Static chambers, used to measure trace gases N_2O and CH_4 soil effluxes, are normally custom made and manually operated. This chamber is preferred when the planned measurement frequency is occasional, and or when multisite comparison is of major interest. It doesn't require electrical power also because the time referred trace gas analysis is done in the lab on in-field collected air samples. Due to the time required to obtain a suitable range of gas concentration (compatible with N_2O and CH_4 analyzers' detection limits), the deployment time is normally in the range of 20 to 60 minutes. This time allows a collection of 3-5 time referred gas samples from the chamber head-space.

Here we give an example of static chamber design and methodology. The chamber was designed by the University of Torino – Italy, and is used in cropland ecosystem (mainly maize).

The chamber has a rectangular base and dimensions of 78.6 cm by 39.3 cm by 20 cm high. The lid surface is protected with cork foil to prevent heating of the headspace; it is covered with adhesive aluminium foil to reflect the light, with a sampling port. It is placed in a water channel welded onto a collar that is inserted 10 cm into the soil. One chamber can be moved on different collars placed on different sites or treatments. Collars can be placed perpendicular to the crop row (e.g. in maize crop having row width of 75 cm) so that both crop row and inter-row areas are contained within each chamber. Anchors are installed each year 1 to 3 before beginning measurements and are removed only for tillage, sowing and harvest operations, and immediately replaced after each operation. Two collars are installed within each replicate of each treatment plot. Inside the collar no plants are present. The sampling protocol is organized in order to sample 3 times during the measurement time: the first as soon as the chamber is closed (time 0), the second after 15 minutes (time 1), and the last after 30 minutes (time 2). The time elapsing between the 0 and 1 sampling time, and between the 1 and 2 sampling time, has to be the same as required by the (Hutchinson and Mosier, 1981) model used to estimate the gas fluxes from the soil. Care must be used to respect the time schedule during the sampling section: if something happens, it is recommended to record the sampling time and then check after flux interpretation.Thirty-millilitres air samples are injected into 12-mL evacuated vials that were sealed with Teflon/silicon septa (Exetainer vial from Labco Limited, High Wycombe, UK) and transported to the laboratory for analysis by gas chromatography.

The gas chromatograph (Agilent 7890A) is equipped with an electron capture detector (ECD) for N_2O determination, with a thermal conductivity detector (TCD) for CO_2 determination, and with a flame ionization detector (FID) for CH_4 determination.

Chamber deployment

It is best to avoid any soil disturbance and compaction around the position chosen for measurements, e.g. by walking on wooden boards placed apart from the chamber.

The collar should be inserted into the soil for 1-5 cm some hours before starting the measurement. Be sure that no leaks are possible around the perimeter of the collar. In stony/gravelled soils or with clods, it is suggested to increase the insertion depth and/or distribute the same soil sieved at 2 mm, around the exterior perimeter of the collar wall, in order to reduce unwanted lateral leaks beneath the collar.

Sealing the chamber/collar junction is required using rubber O-ring or analogous inert material. In the case of deploying the chamber over the collar only for survey measurements (i.e. portable system), a neoprene closed cells sheet is adequate and clamps can be used to fix the chamber to the collar. In unattended systems pay attention that the lid, when open, is in vertical position on North side of the collar to avoid shading soil inside the collar.

When using manual chambers, deploy the chamber over the collar very gently in order to avoid disturbing the CO_2 air concentration with breath. Start collecting data (soil temperature and water content, air temperature, water vapour and pressure) just before chamber is deployed over the collar.

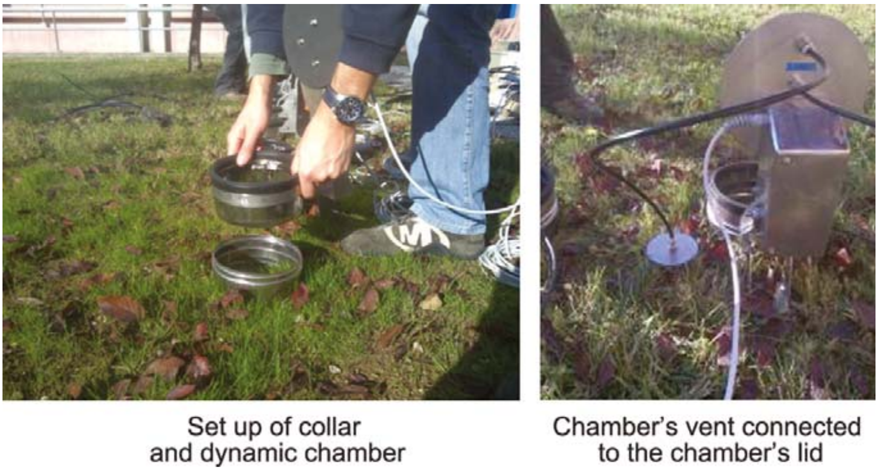

Figure 6. Setting up the dynamic chamber system

Check that the chamber's vent is clean and connect it to the chamber's wall or lid. In case of dynamic chambers, check tubing integrity and restrictions.

DATA CAPTURE

In dynamic chamber systems, equipped with an in-situ analyzer, all measurement data of trace gases are automatically recorded and time referred in digital format by the data-logger.

If using static chambers, the laboratory values must be recorded in a sampling time-referenced record, similar to the record coming from in-situ measurement. This allows any successive control, manipulation and uploading of original data in the data base. Ensure, again, that measurement data (molar fractions of the trace gases time-series) and all environmental parameters are time referred, and unique for each flux measurement, chamber, plot and site in hierarchical order (see below, and also metadata and land use and management protocols). This implies that in case of using vials (as required by NSS-NTFS), one has to assign to each vial the same record's fields listed above. Local Standard Time should be used, not Daylight Saving Time: the best time unit for each data point is seconds from a reference date: e.g. in Microsoft Excel the time “2013-Dec-03 22:13:21” is 41611.92594; this is the number of days from 1900 Jan 01, and the decimal part represent the 80 001 seconds of 86 400 seconds of the day.

The quality check of original data, and the fluxes' computation are done by a well-trained site manager on his desk. All the computed data fluxes F_c will be stored in a

database assigning a quality check flag (QCF) to each flux data. We suggest to use the following QCFs

- 0 if flux data is fitted using NonLinear regression;
- 1 if Linear regression is satisfactory (R2 > 0.99 it is a rough estimate of goodness of fit), this QCF normally applies to very small GHG fluxes (i.e. < 0.5 µmol $CO_2 \cdot m^{-2} \cdot s^{-1}$);
- 2 otherwise.

After data flux are classified for quality, they can be sent to the common database assigning an ID record (see metadata protocol).

METADATA

To compute the GHG fluxes, and to relate them to environmental and ecosystem conditions, all the following data and metadata should be recorded, to be associated to each flux measurement:

- **Ecosystem data** related to the site where chamber's measurement (CO_2, CH_4 or N_2O soil effluxes) are made:
  - soil physical (texture, bulk density and soil classification for each horizon) and chemical parameters (soil organic C and N, pH, CEC);
  - weather climatic data (air temperature and humidity, wind velocity and direction, solar radiation and rainfall/irrigation); these data should be collected year round at a frequency of at least once per day; if possible at half hour intervals. NOTE time, from here thereafter, is always be set to Local Standard Time, don't record daylight saving time);
  - land use (ecosystem) classification; and land use history (forest age, or plant/tree density;
  - above and below Biomass, litter quantity (g dry weight $\cdot m^{-2}$) and type; standing biomass accumulation and litter fall data are required by some methods to compute, subtracting heterotrophic soil CO_2 efflux, Net Biome Production (or C stock change) of large temporal and spatial scales (see Chapin et al. 2012, for more details);
  - date of main phonological stages and Leaf Area Index are important proxy of below ground processes (e.g. autotrophic and total soil respiration);
  - date, time, type and quantity of different management activities or disturbances (e.g. soil tillages, fertilization, sowing, harvest, fire...).
- **Chamber design and deployment data**:
  - Type of chamber measurement: NSS_NFT or NSS_TFS;
  - The analyzer used and its analytical precision for each analyte;
  - Pump flow (In the case of NSS_TFS only); normally it is 0.5-1 $L \cdot min^{-1}$;
  - Include a figure/drawing of the chamber and system components in which the following information could be found:
    - Chamber shape (circular or rectangular) and dimensions;
    - Lid closure type: motorized or manual;
    - Chamber material and radiation insulation;

  - Presence and type of the pressure vent;
  - Methods of preventing leaks (e.g. Lid closure tightness could be ensured with inert material like closed-cells neoprene foil; sealing base and chamber with neoprene or rubber);
  - Air Mixing;
- Total volume of the system (cm^3): volume of the chamber above the soil and volume of tubing and analyzer);
- Area covered by the chamber (cm^2);
- Perimeter of the chamber (cm); area/perimeter is a proxy of possible horizontal leaks beneath the chamber. Negligible radial leaks occur in a short deployment time, i.e. less than 1 min, but increase rapidly during 30 min deployment (Healy et al. 1996).
- Total volume/chamber area ratio (cm) (computed from above).
- Type of measurement (for Soil Respiration only): Total soil respiration or Heterotrophic soil respiration. In the latter indicate the technique used to measure it (e.g. root exclusion). For more details on definition and methods to measure heterotrophic and autotrophic respiration see Kuzyakov et al. (2005), Kuzyakov (2006), Subke et al. (2006) and Chapin et al. (2006).
- Base or collar Installation time (date and time of the day expressed as Local Standard Time not as daylight saving time DST).
- Insertion depth (cm) of the collar, and above ground height of the collar, those data are either used for quality test of flux data, either to compute the volume to be added to the total volume to the system (see above).
- Distance of the center of the chamber from the tree trunks or from the row crop; georeferenced position are preferred to reach quickly the same position in successive measurements. This data are important also to find-out relationships between soil effluxes and plant derived effluxes or processes (e.g. root respiration, water evapo –transpiration, nutrient uptake).

QUALITY ASSURANCE

The soil trace gases effluxes have normally an high variance (CV from 20% to 100%) due either to soil local conditions either to errors in the measurements. The first could be reduced with an adequate number of observations, and the second errors could be avoided following the right chamber design and deployment and by frequent quality checks of data outputs. In this way it should be possible to attain CV in the acceptable range of 10-30%.

Training is necessary before starting measurements. Training is targeted to PhD students or high-professional technicians and lasts 3 days, half dedicated to field and lab instruction, and half to data entry and calculations.

Data and equipment checks

Daily checks of the measurement data are valuable to check for automatic system malfunctioning.

For automatic stand-alone systems, it is recommended to download data on the office desktop. In this case a GSM-modem is very helpful to download and check data daily. With the modem communication one can also manage from the office the remote unattended system. The remote connection may require specific hardware and software provided or suggested by the Control Unit (data logger) manufacturer.

If computed flux data are out of acceptable range (considering also the average of recent flux data), first check the original data points of each measurement to see if the data show a reasonable trend (a convex one) or the difference between initial and final concentrations is too high or too low (below detectable limits of the system, ie trend is too flat). There is commonly some leak in the pneumatic circuit, in the sealing of chamber's components, in the chamber/collar-soil joint; or there may be some sudden change in environmental conditions (e.g. windy conditions, an heavy rain, a rodent perforating the tubing, dew formation in the tubing, insufficient chamber radiation shielding, a micrometeorological condition of strong air stability ie at sunrise and sunset, etc). The analysis of environmental conditions gives often the reason of sudden changes in flux data during the day. Also changes in soil porosity and in the uniformity of soil profile is influencing fluxes and their accuracy (Venterea et al. 2008) and addresses the best deployment and computation choices.

The chambers need regular checks: some insects (*Ostrinia nubilalis* European corn borer larvae) can perforate the PE tubing left above the soil. Also undertake checks of the pneumatic circuit integrity, i.e. pay attention to the pump leakeage or flow. Analyzers also require maintenance checks and or calibration; follow the manufacturer's instructions.

Specific quality checks for NSS-NTFS

Specific solutions to be realized for quality assurance for static chambers are the following:

- Avoid excessive temperature rise in the measurement chamber by using shading or reflecting cover.
- Use only vials for specific gas measurements to limit diffusion across septa. Butyl rubber sets are generally accepted, but new double septa Teflon silicon are being tested. Conservation must be limited to 6 hours using butyl rubber septa.
- Vials are evacuated before use, then injected with sample gas to reach at least 2 atm pressure (e.g. 30 ml sample in a 12 ml vial), to avoid mass flow contamination from the atmosphere. The extra volume makes it possible to repeat GC analysis, if needed.
- GC analysis should be accomplished as near as possible to sampling moment. If GC is present, at the same day of sampling. If you need to end your vials for GC analysis, keep them at low temperature (4-10°C).
- The GC standard calibration curve is not used; a specific calibration curve is used instead at each measurement session. To this purpose, it is necessary to include a proper number of gas standards (at known concentration) within every set of samples.

If users intend to buy a new GC, it is advisable to contact research group leaded by Carlo Grignani, since instrument need a proper customization. Otherwise users must send their samples to the lab of research group leaded by Carlo Grignani.

REFERENCES

Chapin, F.S., Woodwell, G.M., Randerson, J.T., Rastetter, E.B., Lovett, G.M., Baldocchi, D.D., Clark, D.A., Harmon, M.E., Schimel, D.S., Valentini, R., Wirth, C., Aber, J.D., Cole, J.J., Goulden, M.L., Harden, J.W., Heimann, M., Howarth, R.W., Matson, P.A., McGuire, A.D., Melillo, J.M., Mooney, H.A., Neff, J.C., Houghton, R.A., Pace, M.L., Ryan, M.G., Running, S.W., Sala, O.E., Schlesinger, W.H., Schulze, E.D. 2006. Reconciling carbon-cycle concepts, terminology, and methods. *Ecosystems* 9: 1041-1050.

Conant, R.T. et al. 2011. Temperature and soil organic matter decomposition rates – synthesis of current knowledge and a way forward. *Global Change Biology* 17: 3392-3404. doy: 10.1111/j.1365-2486.2011.02496.x.

Davidson, E.A., Savage, K., Verchot, L.V., Navarro, R. 2002. Minimizing artifacts and biases in chamber-based measurements of soil respiration. *Agricultural and Forest Meteorology* 113: 21-37.

Delle Vedove, G., Alberti, G., Zuliani, M., Peressotti, A., Inglima, I., Zerbi, G. 2007. Automated Monitoring of Soil Respiration: an Improved Automatic Chamber System. *Italian J. of Agronomy* 4: 377-382.

Healy, R.W., Striegl, R.G., Russell, T.F., Hutchinson, G.L., Livingston, G.P. 1996. Numerical evaluation of static-chamber measurements of soil-atmosphere gas exchange: Identification of physical processes. *Soil Science Society of America Journal* 60: 740-747.

Heinemeyer, A., McNamara, N.P. 2011. Comparing the closed static versus the closed dynamic chamber flux methodology: Implications for soil respiration studies. *Plant and Soil* 346: 145-151.

Hutchinson, G.L., Livingston, G.P. 2001. Vents and seals in non-steady-state chambers used for measuring gas exchange between soil and the atmosphere. *European Journal of Soil Science* 52: 675-682.

Iqbal, J., Castellano, M.J., Parkin, T.B. 2012. Evaluation of photoacoustic infrared spectroscopy for simultaneous measurement of N2O and CO_2 gas concentrations and fluxes at the soil surface. *Global Change Biology*. DOI: 10.1111/gcb.12021.

Jarecki, M.K., Parkin, T.B., Chan, A.S.K., Hatfield, J.L., Jones, R. 2008. Comparison of DAYCENT-simulated and measured nitrous oxide emissions from a corn field. *Journal of Environmental Quality* 37: 1685-1690.

Kutzbach, L., Schneider, J., Sachs, T., Giebels, M., Nykänen, H., Shurpali, N.J., Martikainen, P.J., Alm, J., Wilmking, M. 2007. CO_2 flux determination by closed-chamber methods can be seriously biased by inappropriate application of linear regression. *Biogeosciences* 4: 1005-1025, doi:10.5194/bg-4-1005-2007.

Kuzyakov, Y., Larionova, A.A. 2005. Root and rhizomicrobial respiration: A review of approaches to estimate respiration by autotrophic and heterotrophic organisms in soil. *Journal of Plant Nutrition and Soil Science* 168: 503-520.

Kuzyakov, Y. 2006. Sources of CO_2 efflux from soil and review of partitioning methods. *Soil Biology and Biochemistry* 38: 425-448.

Livingston, G.P., Hutchinson, G.L. 1995. Enclosure-based measurement of trace gas exchange: applications and sources of error. In: P.A. Matson, R.C. Harriss (eds.), *Biogenic Trace Gases: Measuring Emissions from Soil and Water*. Blackwell Science, Cambridge, pp. 14-50.

Livingston, G.P., Hutchinson, G.L., Spartalian, K. 2006. Trace Gas Emission in Chambers: A Non-Steady-State Diffusion Model. *Soil Sci. Soc. Am. J.* 70: 1459-1469.

Parkin, T.B., Kaspar, T.C. 2003.Temperature Controls on Diurnal Carbon Dioxide Flux: Implications for Estimating Soil Carbon Loss. *Soil Science Society of America Journal* 67: 1763-1772.

Parkin, T.B. 2008. Effect of sampling frequency on estimates of cumulative nitrous oxide emissions. *Journal of Environmental Quality* 37: 1390-1395.

Parkin, T.B., Venterea, R.T. 2010. Sampling Protocols. Chapter 3. Chamber-Based Trace Gas Flux Measurements. In: R.F. Follett (ed.), *Sampling Protocols*. p. 3-1 to 3-39. Available at: www.ars.usda.gov/research/GRACEnet.

Parkin, T.B., Venterea, R.T., Hargreaves, S.K. 2012. Calculating the detection limits of chamber-based soil greenhouse gas flux measurements. *Journal of Environmental Quality* 41: 705-715. DOY: 10.2134/jeq2011.0394.

Pedersen et al. 2010. A comprehensive approach to soil-atmosphere trace-gas flux estimation with static chambers. *Europ. J. Soil Sc.* 61(6): 888-902.

Predotova, M., Kretschmann, R., Gebauer, J., Buerkert, A. 2011. Effects of cuvette surface material on ammonia-, nitrous oxide-, carbon dioxide-, and methane-concentration measurements. *J. Plant Nutr. Soil Sci.* 174: 347-349.

Pumpanen, J. et al. 2004. Comparison of different chamber techniques for measuring soil CO_2 efflux. *Agricultural and Forest Meteorology* 123(3-4): 159-176.

Rochette, P., Eriksen-Hamel, N.S. 2008. Chamber Measurements of Soil Nitrous Oxide Flux: Are Absolute Values Reliable? *Soil Sci. Soc. Am. J.* 72: 331-342.

Rochette, P. 2011. Towards a standard non-steady-state chamber methodology for measuring soil N2O emissions. *Animal Feed Science and Technology* 166-167: 141-146.

Savage, K.E., Davidson, E.A. 2003. A comparison of manual and automated systems for soil CO_2 flux measurements: trade-offs between spatial and temporal resolution. *Journal of Experimental Botany* 54: 891-899.

Smith, P., Davies, C.A., Ogle, S., Zanchi, G., Bellarby, J., Bird, N., Boddey, R.M., McNamara, N.P., Powlson, D., Cowie, A., van Noordwijk, M., Davis, S.C., Richter, D.D.B., Kryzanowski, L., van Wijk, M.T., Stuart, J., Kirton, A., Eggar, D., Newton-Cross, G., Adhya, T.K., Braimoh, A.K. 2012. Towards an integrated global framework to assess the impacts of land use and management change on soil carbon: Current capability and future vision. *Global Change Biology* 18: 2089-2101.

Subke, J.A., Inglima, I., Cotrufo, M.F. 2006. Trends and methodological impacts in soil CO_2 efflux partitioning: A meta-analytical review. *Global Change Biology* 12: 921-943.

Venterea, R.T., Baker, J.M. 2008. Effects of soil physical nonuniformity on chamber-based gas flux estimates. *Soil Science Society of America Journal* 72: 1410-1417.

Venterea, R.T., Spokas, K.A., Baker, J.M. 2009. Accuracy and Precision Analysis of Chamber-Based Nitrous Oxide Gas Flux Estimates. *Soil Science Society of America Journal* 73: 1087-1093.

Venterea, R.T. 2010. Simplified Method for Quantifying Theoretical Underestimation of Chamber-Based Trace Gas Fluxes. *J. Environ. Qual.* 39: 126-135.

Xu, L. et al. 2006. On maintaining pressure equilibrium between a soil CO_2 flux chamber and the ambient air. *Journal of Geophysical Research* 111, D08S10.

Welles, J.M., Demetriades-Shah, T.H., McDermitt, D.K. 2001. Considerations for measuring ground CO_2 effluxes with chambers. *Chemical Geology* 177: 3-13.

Welles, J.M., McDermitt, D.K. 2005. Measuring Carbon Dioxide in the Atmosphere. In Micrometeorology in Agricultural Systems. *Agronomy Monograph* 47: 287-320.

ExpeER course on standarised ecosystem protocols,
The Netherlands 2013

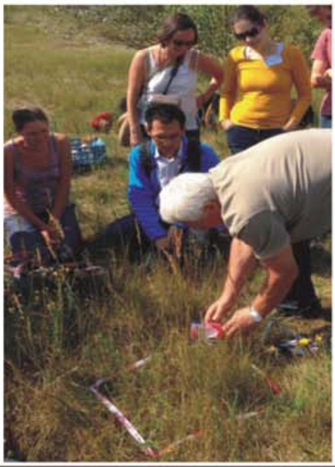

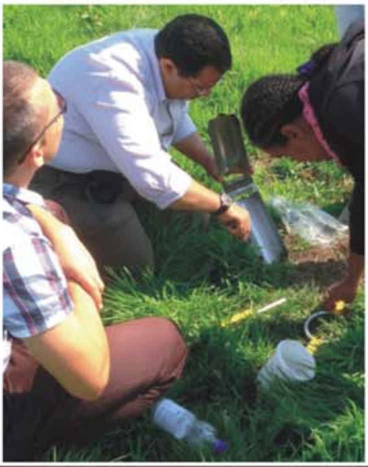

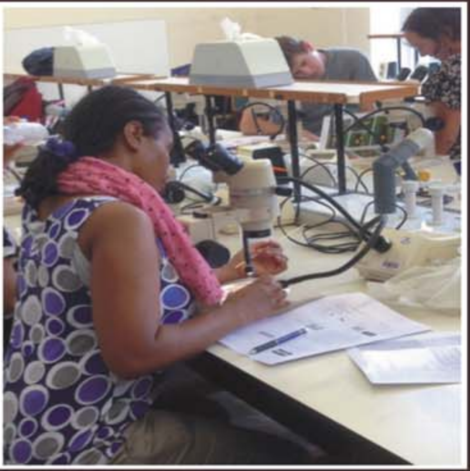

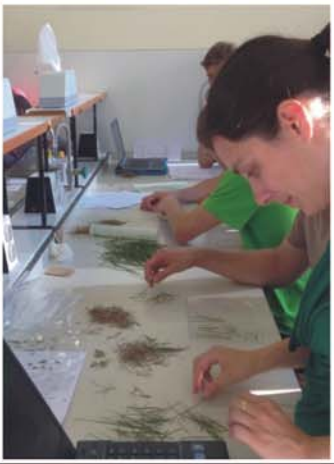

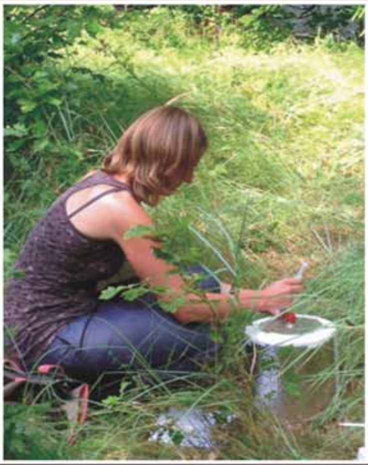

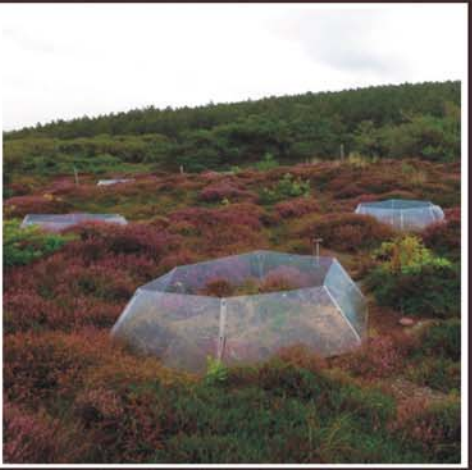

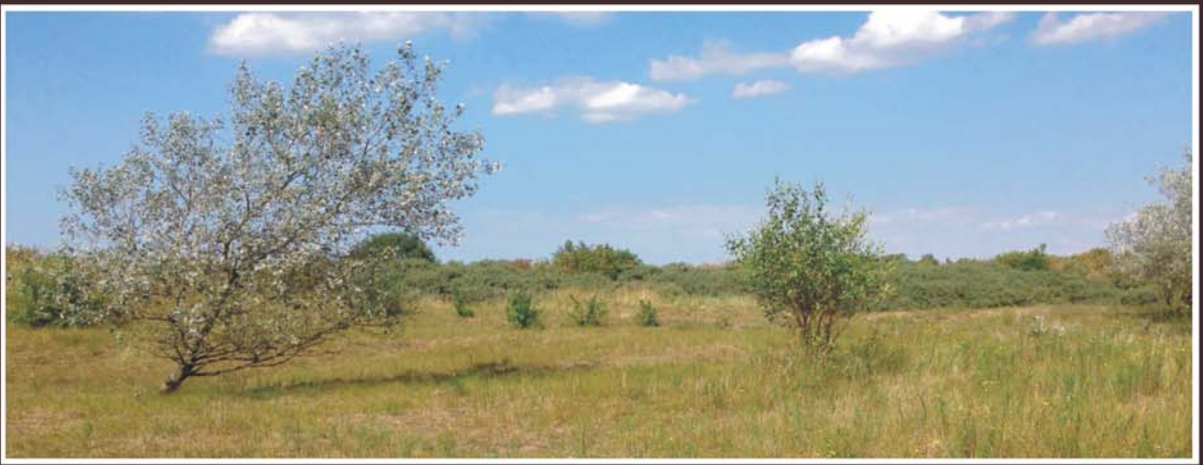

ExpeER

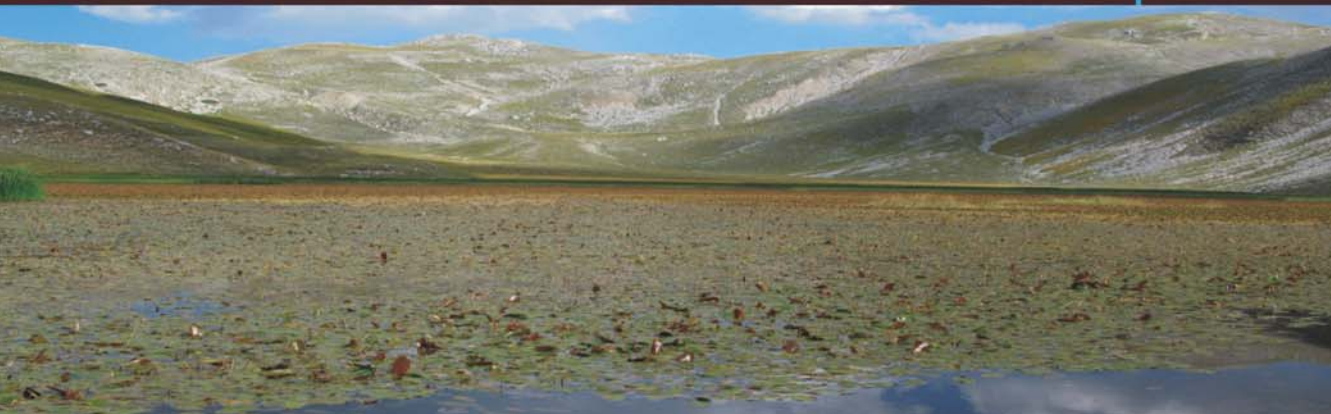

ExpeER (Experimentation in Ecosytem Research) brings together existing highly instrumented European infrastructures in the field of ecosystem research to improve their research capacity and to encourage their wider use, in particular through transnational access to various types of facilities.

www.expeeronline.eu ExpeER

This project has received funding from the European Union's Seventh Programme for research, technological development and demonstration under grant agreement No 262060.
